# Supplementary material for: Single-Nanoparticle Tracking with Angstrom Localization Precision and Microsecond Time Resolution
Source: Biophys J. 2018 Nov 17;115(12):2413–27. doi: 10.1016/j.bpj.2018.11.016 (PMC6302141; doi:10.1016/j.bpj.2018.11.016)
Supplement: Document S1. Figs. S1–S17 [file mmc1.pdf]

Biophysical Journal, Volume *115*

Supplemental Information

Single-Nanoparticle Tracking with Angstrom Localization Precision and Microsecond Time Resolution

Jun Ando, Akihiko Nakamura, Akasit Visootsat, Mayuko Yamamoto, Chihong Song, Kazuyoshi Murata, and Ryota Iino

Supplemental Information

Single-nanoparticle tracking with angstrom localization precision and microsecond time resolution

Jun Ando,[1,2] Akihiko Nakamura,[1,2] Akasit Visootsat,[1,2] Mayuko Yamamoto,[1] Chihong Song,[3] Kazuyoshi Murata,[3] and Ryota Iino[1,2]

[1]Institute for Molecular Science, National Institutes of Natural Sciences, Okazaki, Aichi, Japan; [2]The Graduate University for Advanced Studies (SOKENDAI), Hayama, Kanagawa, Japan; [3]National Institute for Physiological Sciences, National Institutes of Natural Sciences, Okazaki, Aichi, Japan

Contents

Fig. S1-S17

Video S1, S2

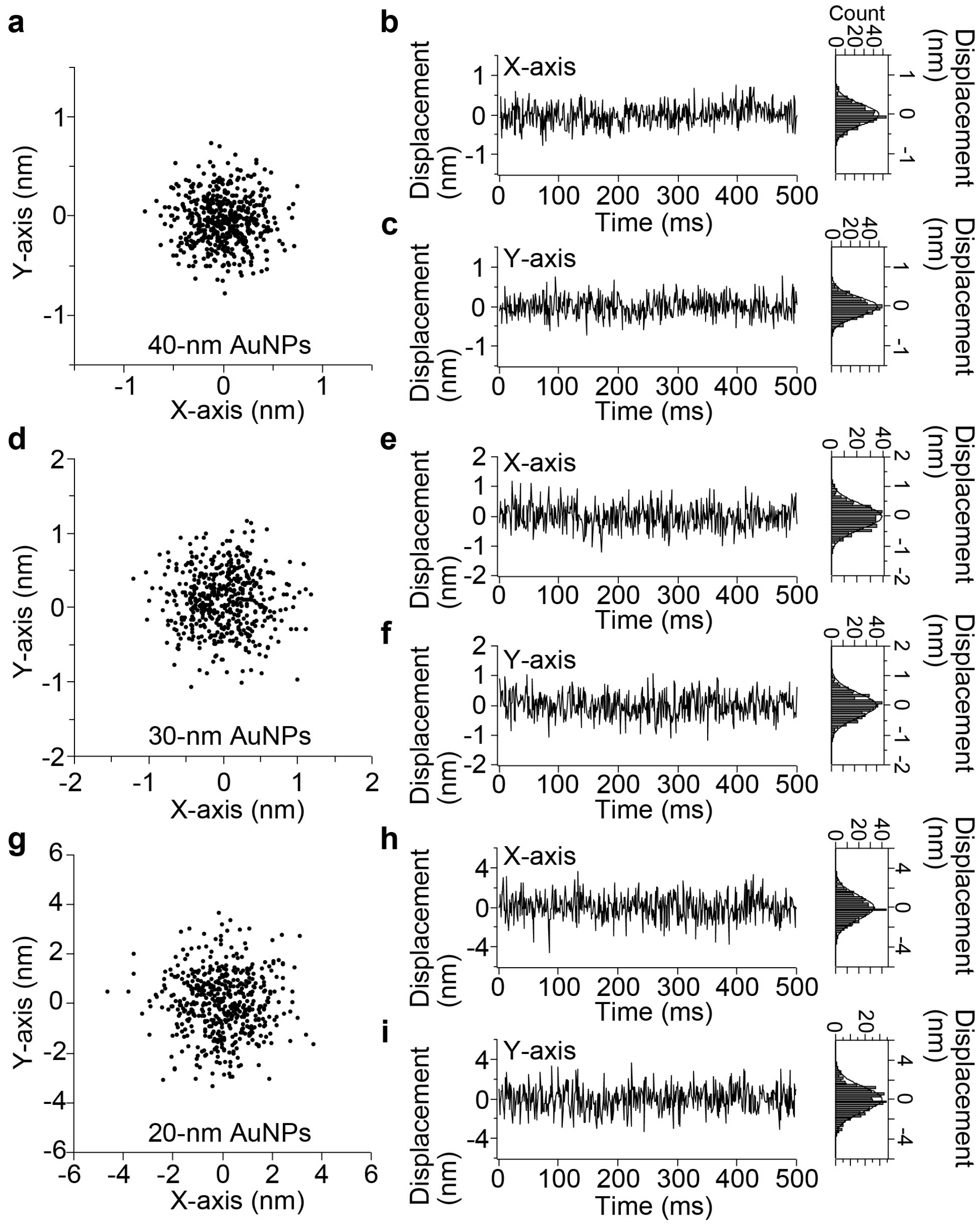

Fig. S1. Examples of two-dimensional plots, time courses, and histograms with Gaussian fits at the center for the dark-field images of (*a-c*) 40 nm, (*d-f*) 30 nm and (*g-i*) 20 nm AuNPs. The image was taken at 1 ms time resolution for 0.5 s with 10 μW/μm^2 laser intensity.

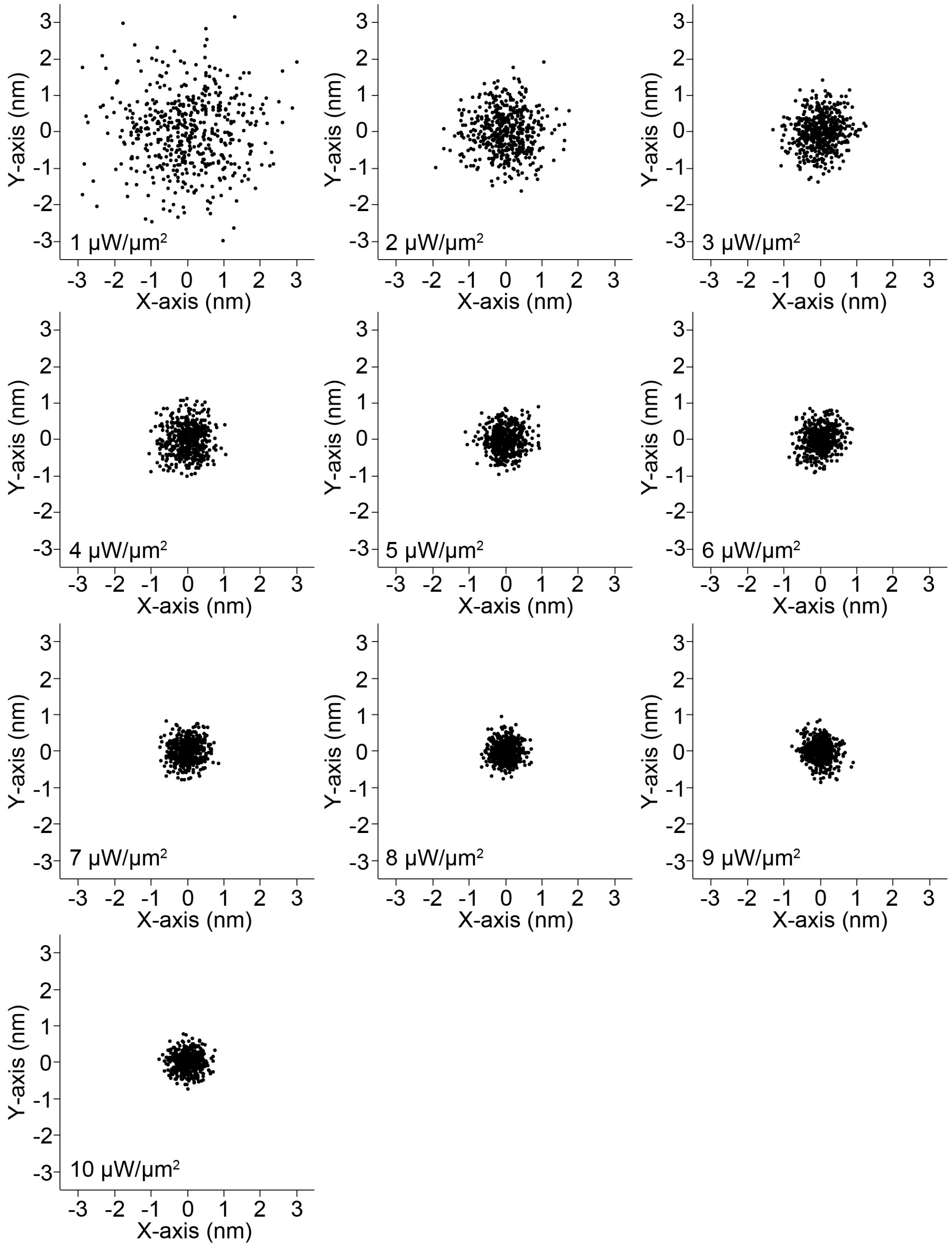

Fig. S2. Examples of two-dimensional plots in the center of the dark-field image of 40 nm AuNPs with laser intensities ranging from 1 to 10 μW/μm^2.

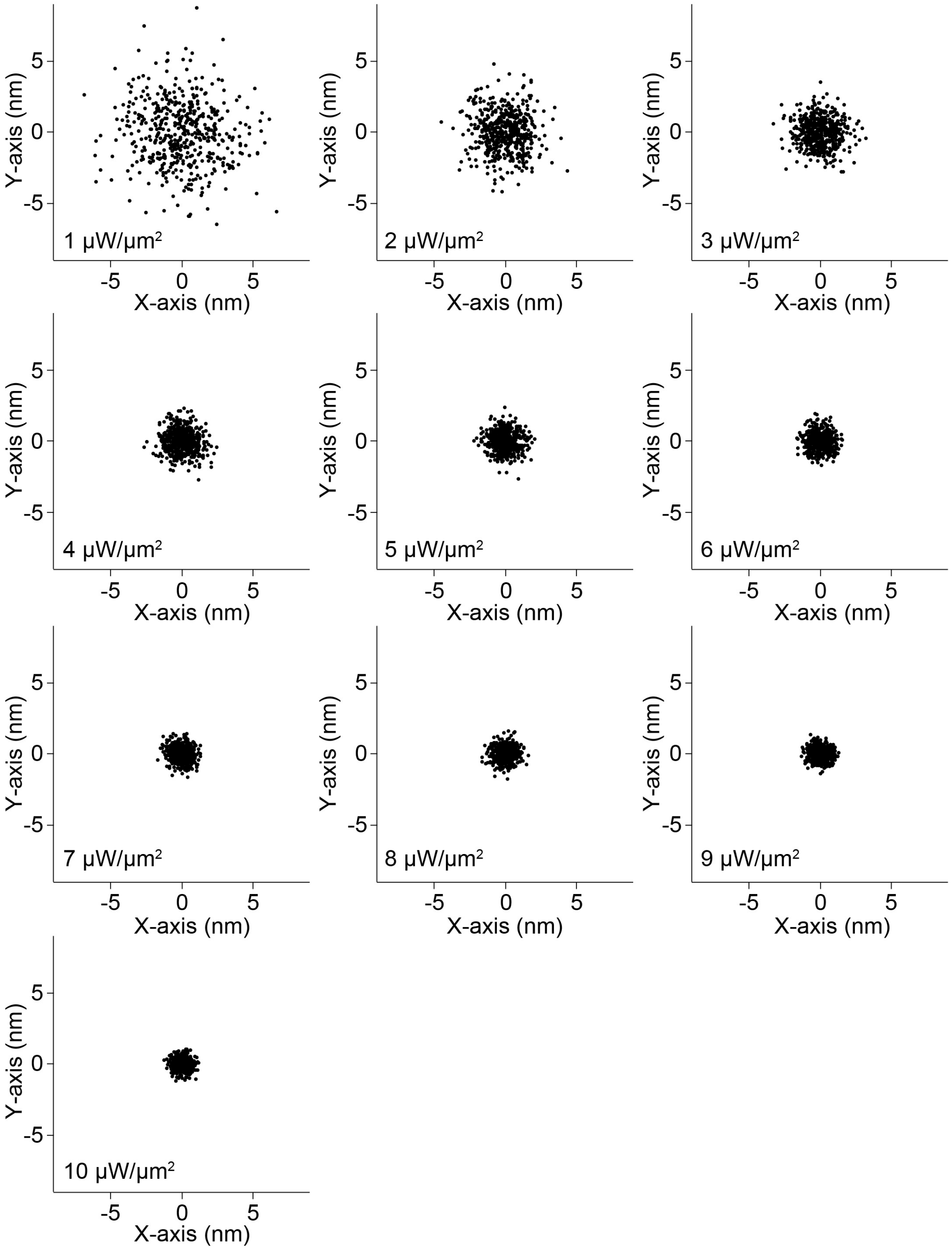

Fig. S3. Examples of two-dimensional plots in the center of the dark-field image of 30 nm AuNPs with laser intensities ranging from 1 to 10 μW/μm^2.

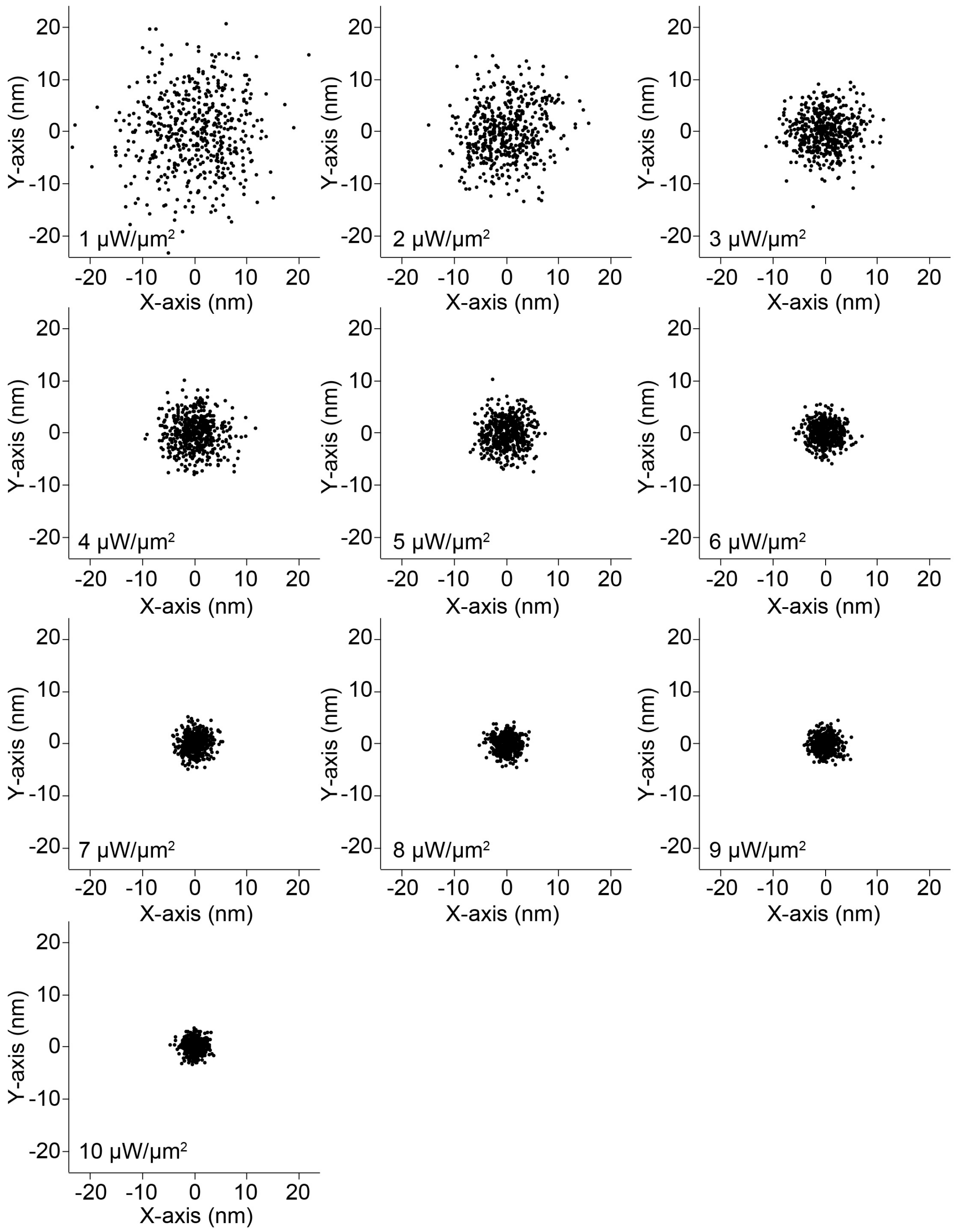

Fig. S4. Examples of two-dimensional plots in the center of the dark-field image of 20 nm AuNPs with laser intensities ranging from 1 to 10 μW/μm^2.

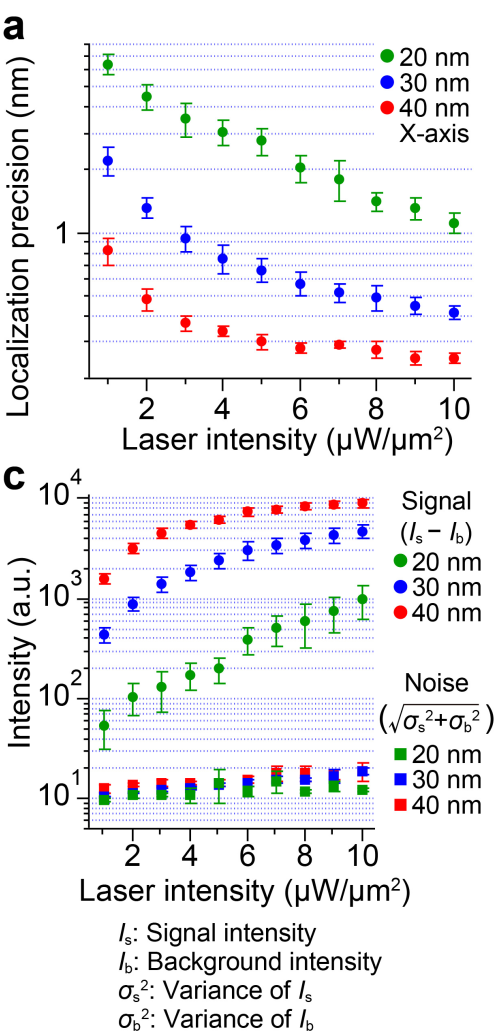

Fig. S5. Laser intensity dependence of the localization precision. (*a,b*) Localization precision of the dark-field image of 20 nm, 30 nm, and 40 nm AuNPs with different laser intensities along the (a) X- and (b) Y-axis ($n = 5$). The image was captured at 1 ms time resolution. Green, blue, and red circles represent 20 nm, 30 nm, and 40 nm AuNPs, respectively. (*c*) Signal and noise of the dark-field images of 20 nm, 30 nm, and 40 nm AuNPs with different laser intensities. Circles and rectangles with green, blue, and red colors represent signal and noise of 20 nm, 30 nm, and 40 nm AuNPs, respectively. (*d*) Signal-to-noise ratio of 20 nm, 30 nm, and 40 nm AuNPs at different laser intensities. Green, blue, and red circles represent 20 nm, 30 nm, and 40 nm AuNPs, respectively.

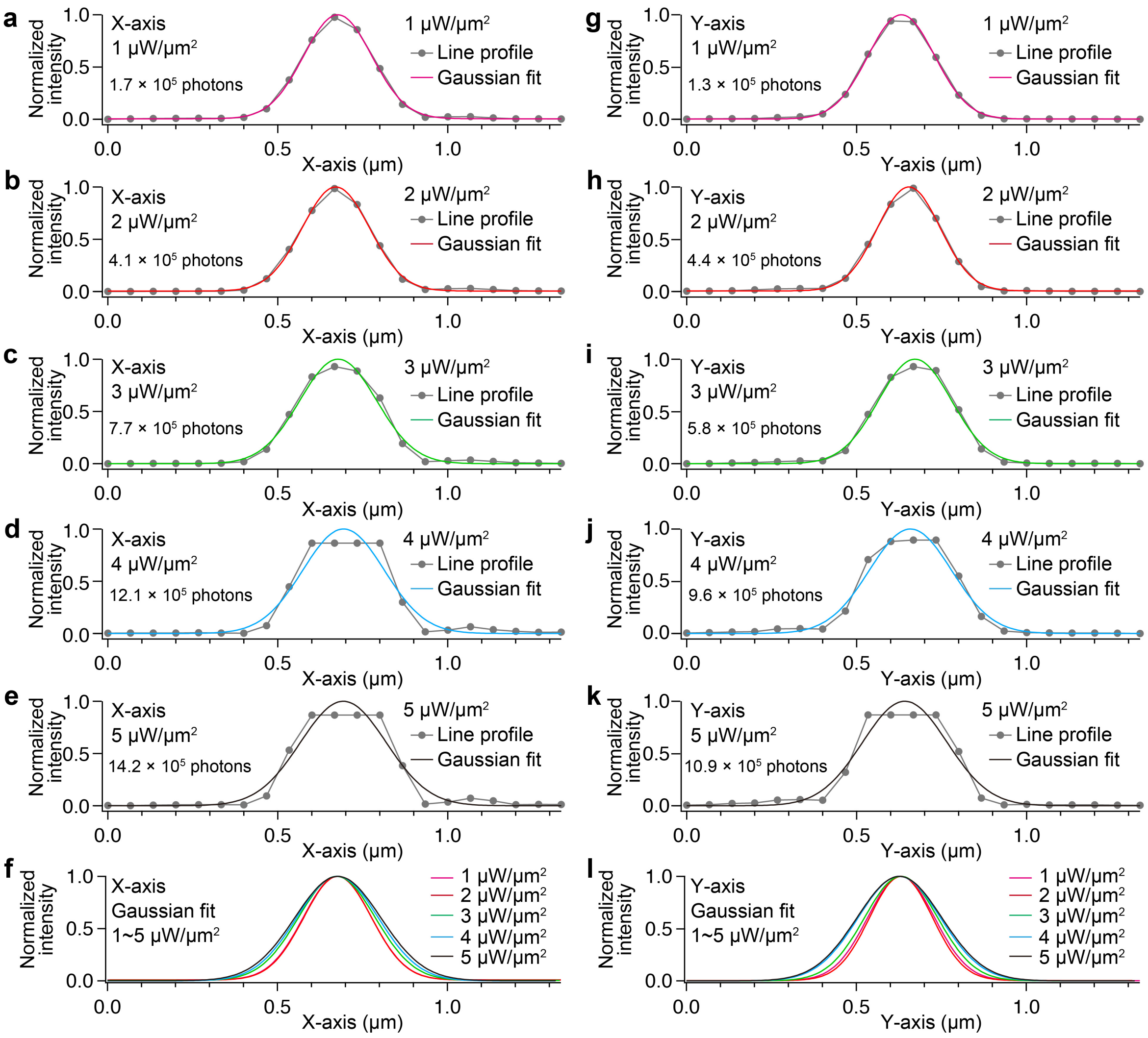

Fig. S6. Examples of line profile and Gaussian fit along X- (*a*-*e*) and Y-axis (*g*-*k*) for the dark-field image of 40 nm AuNPs. Images were taken at 1 ms time resolution with laser intensities of (*a*, *g*) 1 μW/μm^2, (*b*, *h*) 2 μW/μm^2, (*c*, *i*) 3 μW/μm^2, (*d*, *j*) 4 μW/μm^2, and (*e*, *k*) 5 μW/μm^2, respectively. (*f* and *l*) Overlay of the Gaussian fits in *a*-*e* (*f*) and *g*-*k* (*l*). Estimated photon numbers from AuNPs are also shown.

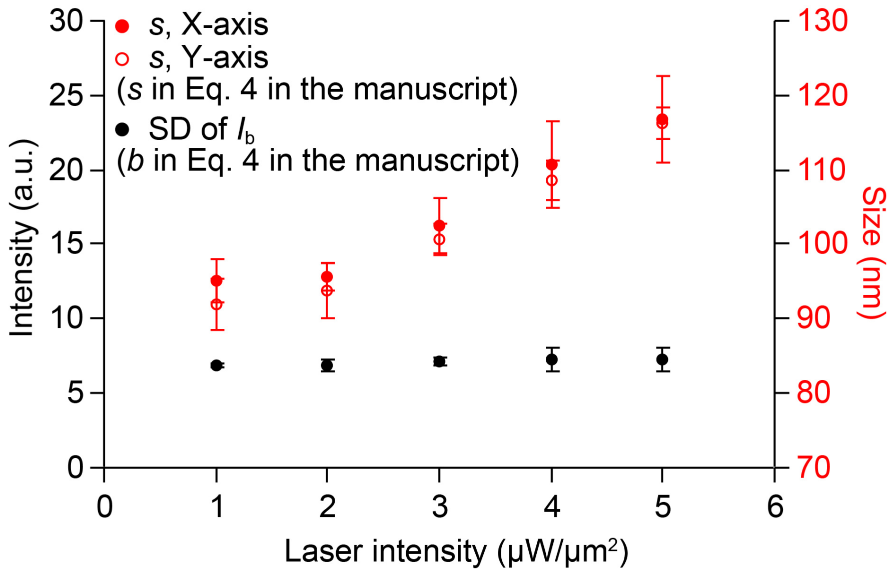

Fig. S7. Laser intensity dependences of the width of dark-field images of 40 nm AuNPs along X- and Y-axis (*s* in Eq. 4 in the manuscript) and standard deviation of background intensity (SD of I_b, *b* in Eq. 4 in the manuscript). Images were taken at 1 ms time resolution with different laser intensities of 1–5 μW/μm^2. Background intensity was measured at neighboring area of the AuNP.

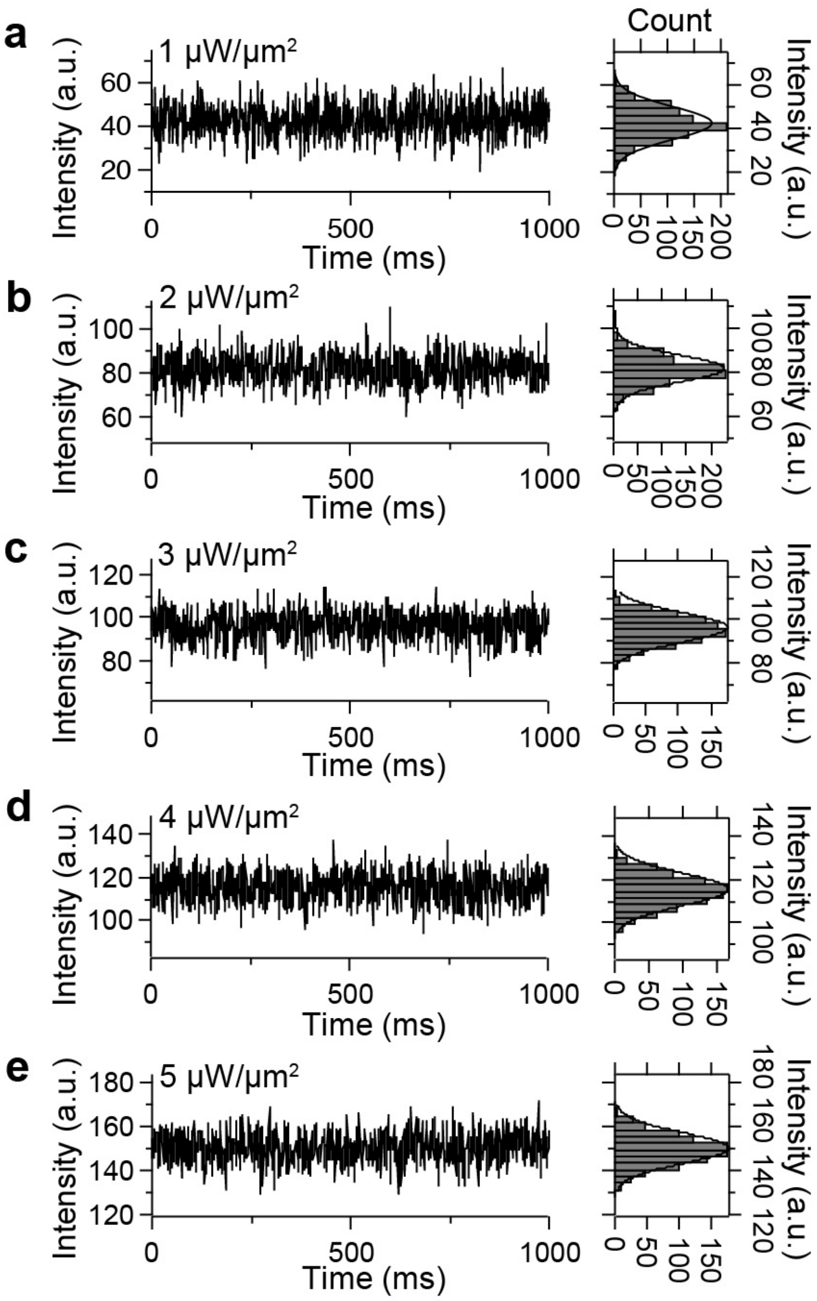

Fig. S8. Examples of the time courses and histograms with Gaussian fits of the background intensity in dark-field images of 40 nm AuNPs with laser intensities at (*a*) 1 μW/μm^2, (*b*) 2 μW/μm^2, (*c*) 3 μW/μm^2, (*d*) 4 μW/μm^2, and (*e*) 5 μW/μm^2. Background intensity was measured at neighboring area of the AuNP. Images were taken at 1 ms time resolution for 1.0 s.

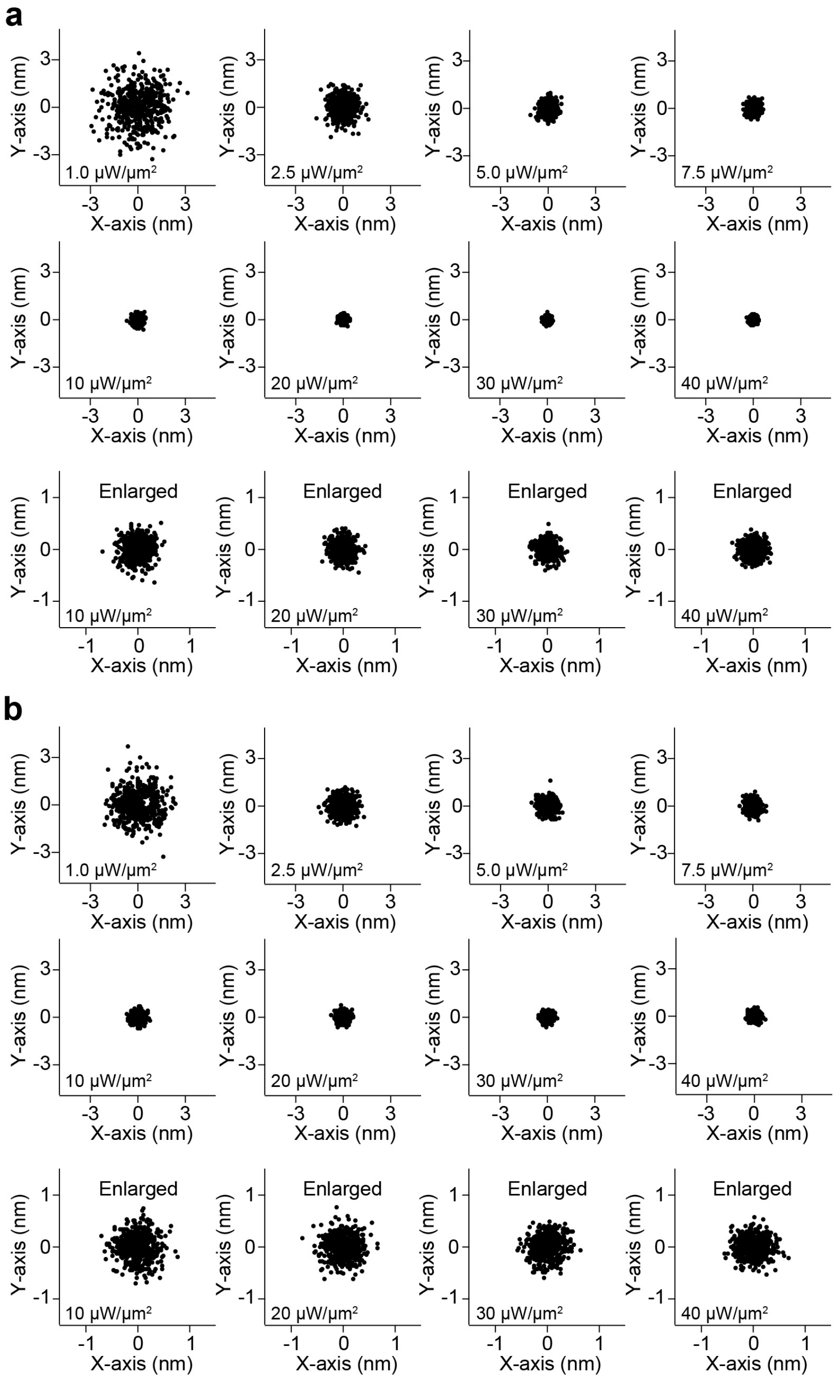

Fig. S9. Examples of two-dimensional plots at the center of the dark-field image of 40 nm AuNPs with laser intensities ranging from 1 to 40 μW/μm^2. The images were taken at 1 ms time resolution for 0.5 s and at image pixel sizes of (*a*) 31.6 nm/pixel and (*b*) 67.6 nm/pixel, respectively.

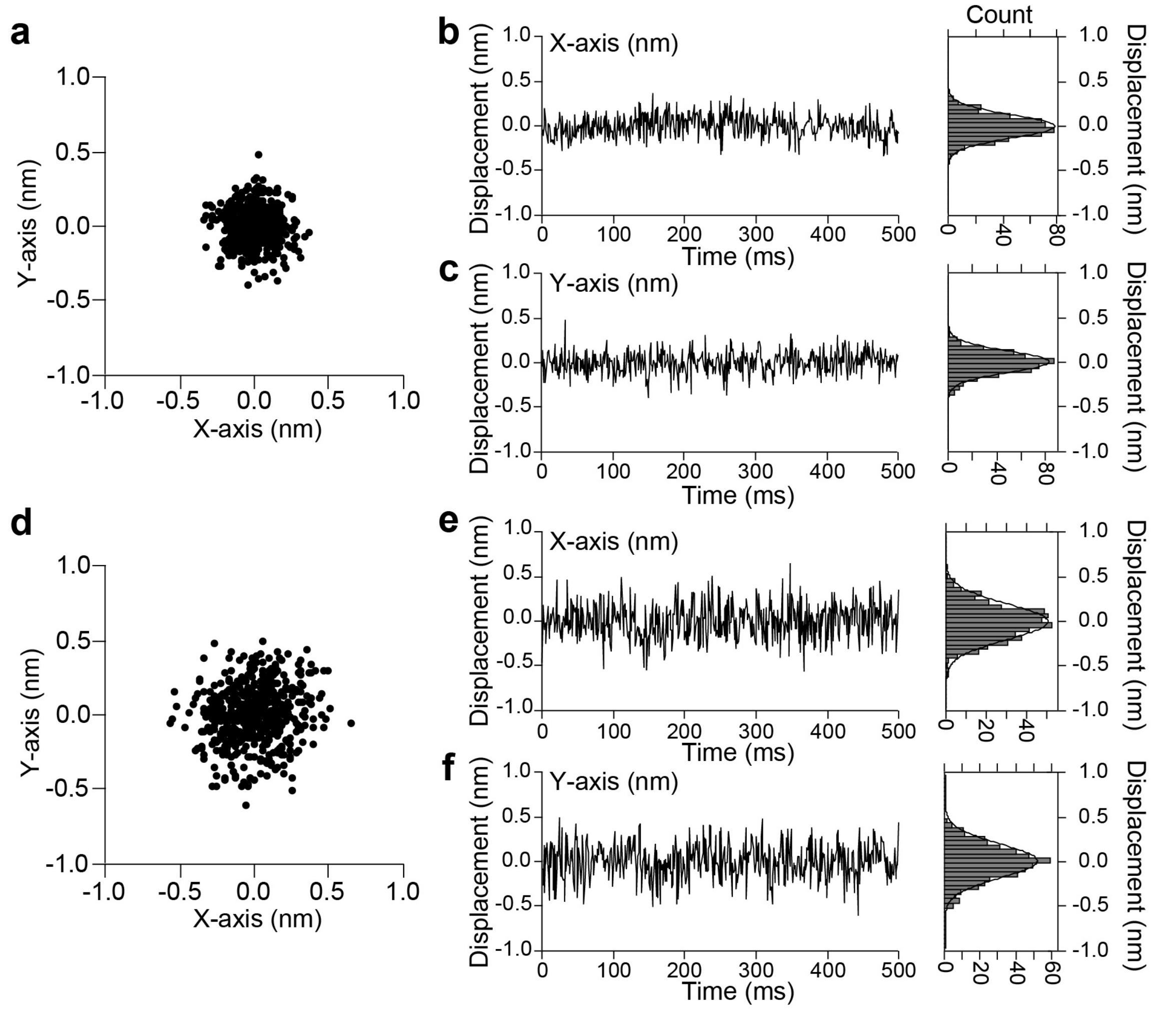

Fig. S10. Examples of two-dimensional plots, time courses, and histograms with Gaussian fits at the center of the dark-field image of 40 nm AuNPs, taken with image pixel sizes of (*a-c*) 31.6 nm/pixel and (*d-f*) 67.6 nm/pixel. The images were taken at 1 ms time resolution for 0.5 s and 30 μW/μm^2 laser intensity.

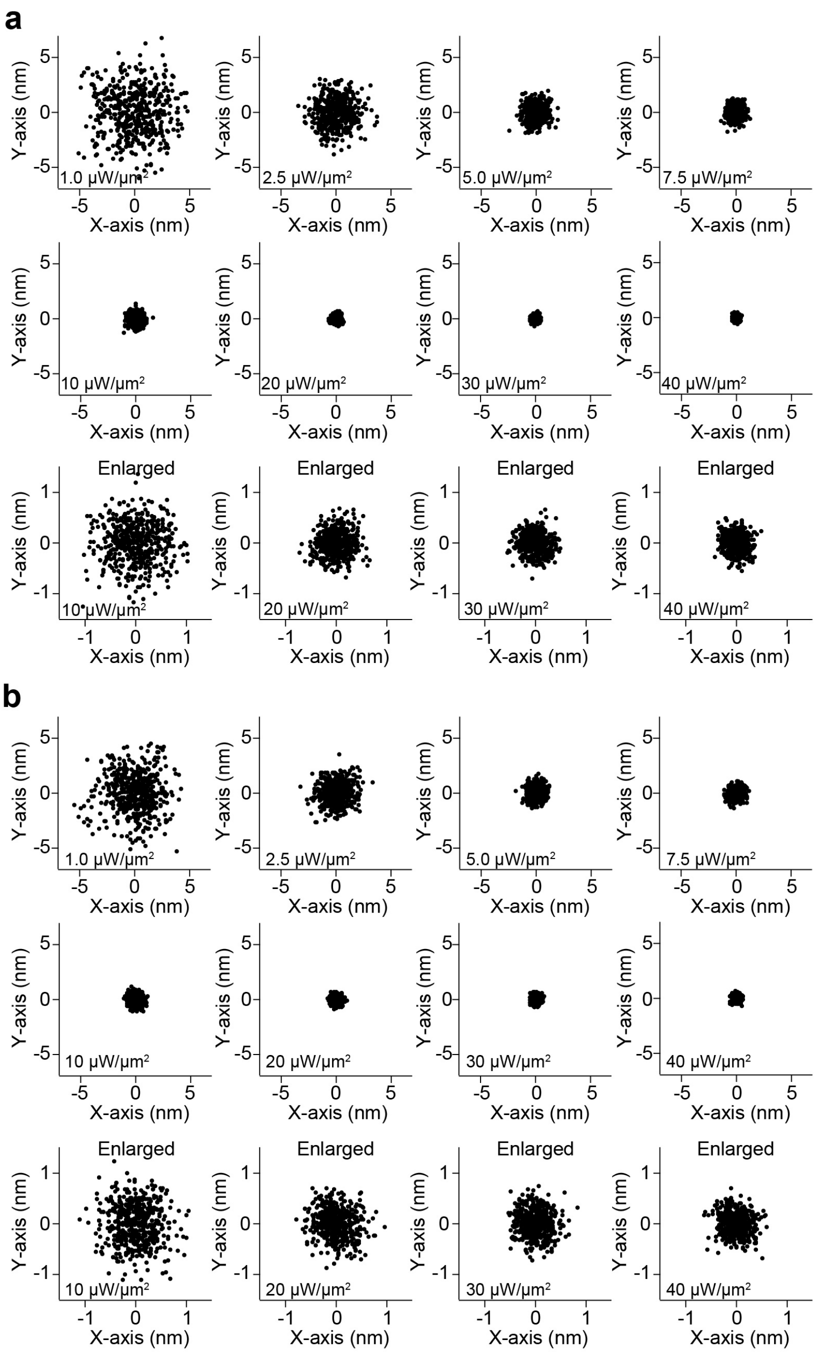

Fig. S11. Examples of two-dimensional plots in the center of the dark-field image of 30 nm AuNPs with laser intensities ranging from 1 to 40 μW/μm^2. The images were taken at 1 ms time resolution for 0.5 s. The images were taken at image pixel sizes of (*a*) 31.6 nm/pixel and (*b*) 67.6 nm/pixel.

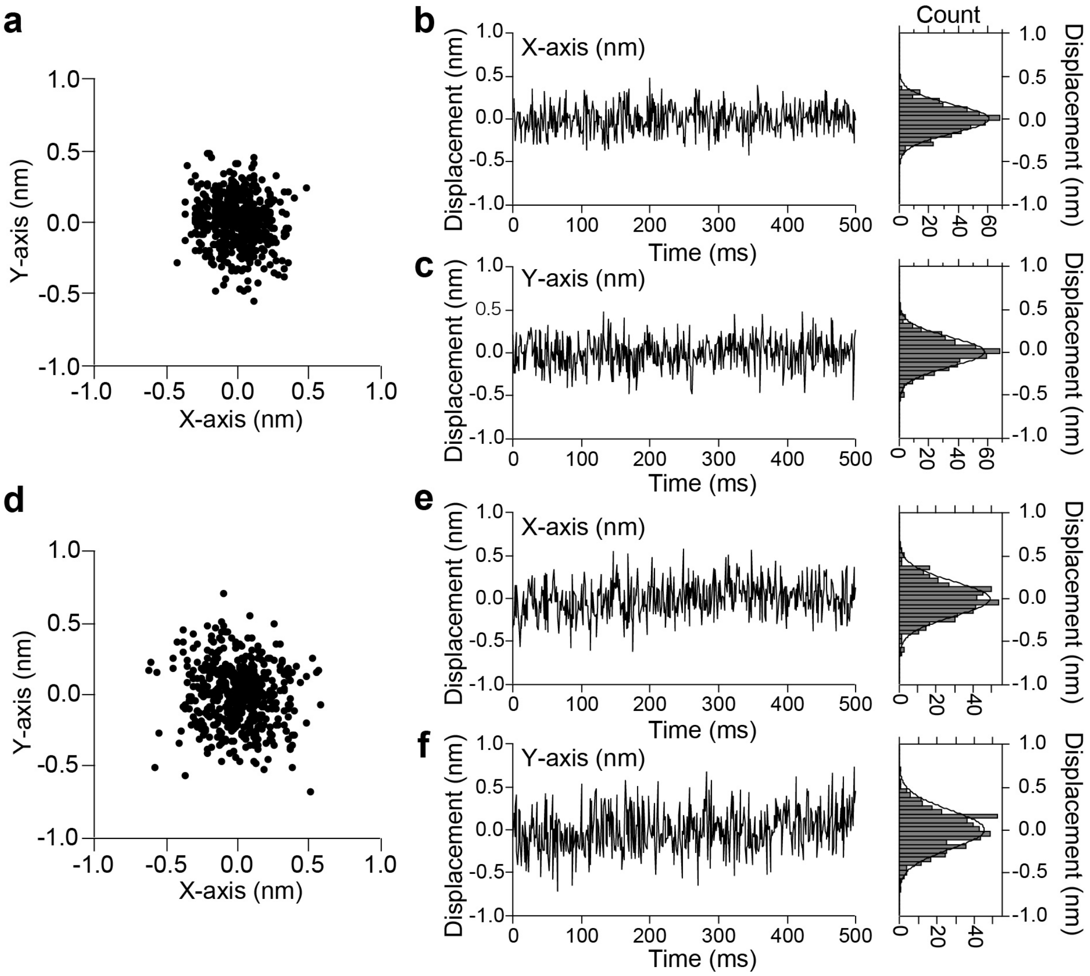

Fig. S12. Examples of two-dimensional plots, time courses, and histograms with Gaussian fits at the center of the dark-field image of 30 nm AuNPs, taken with image pixel sizes of (*a-c*) 31.6 nm/pixel and (*d-f*) 67.6 nm/pixel. The images were taken at 1 ms time resolution for 0.5 s and 40 μW/μm^2 laser intensity.

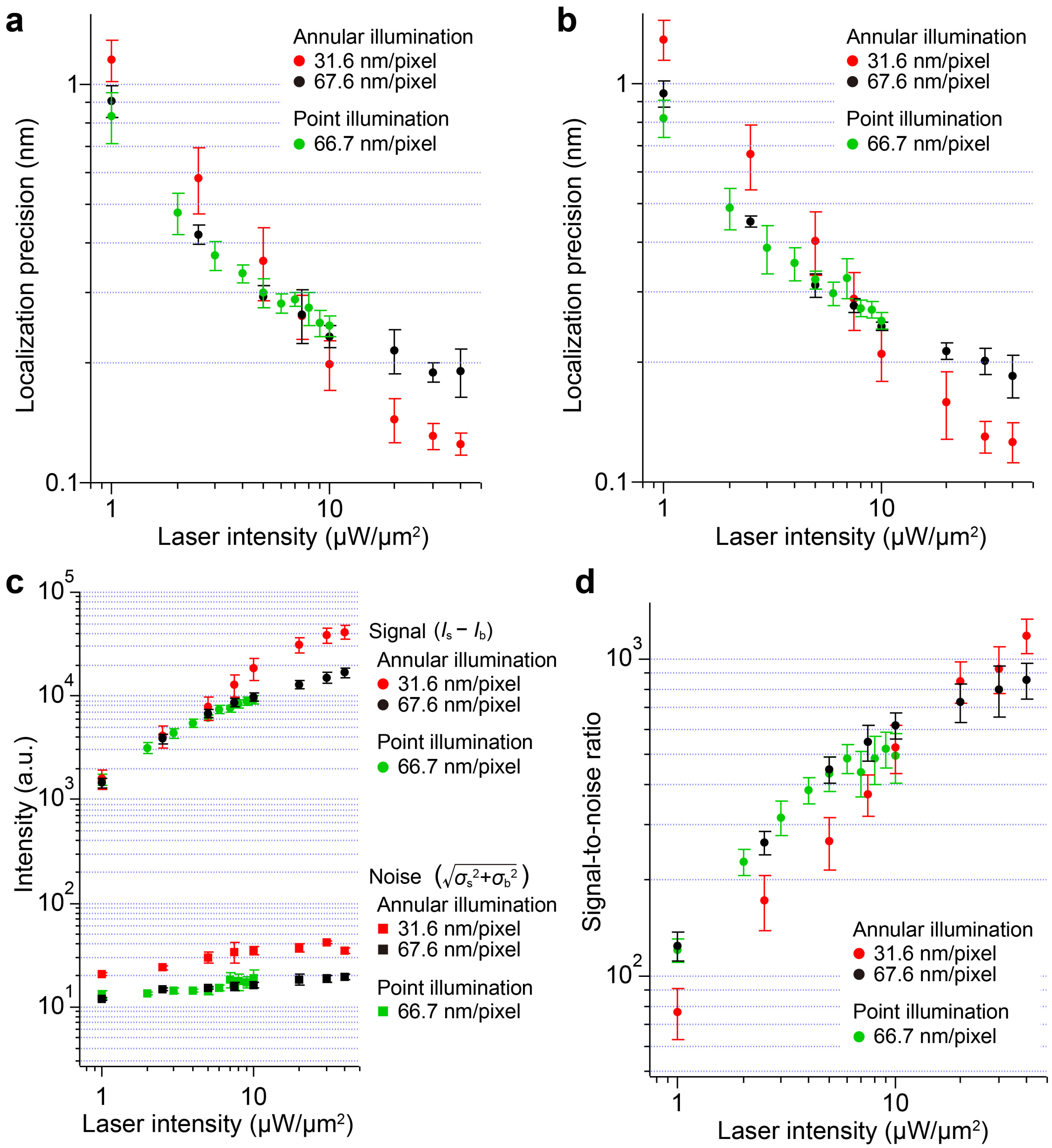

Fig. S13. Comparison of localization precision, signal, noise, and signal-to-noise ratio for 40 nm AuNP images between annular and point illumination total internal reflection dark-field microscopy. Images were taken at 1 ms time resolution. (*a, b*) Log-log plots between laser intensity and localization precision along the (*a*) X- and (*b*) Y-axis (n = 5). Red and black circles represent annular illumination with 31.6 nm/pixel and 67.6 nm/pixel, respectively. Green circles

represent point illumination with 66.7 nm/pixel. (*c*) Log-log plots between laser intensity and signal (circles) or noise (squares) (n = 5). Red and black symbols represent annular illumination with 31.6 nm/pixel and 67.6 nm/pixel, respectively. Green symbols represent point illumination with 66.7 nm/pixel. (*d*) Log-log plots between laser intensity and signal-to-noise ratio (n = 5). Red and black circles represent annular illumination with 31.6 nm/pixel and 67.6 nm/pixel, respectively. Green circles represent point illumination with 66.7 nm/pixel.

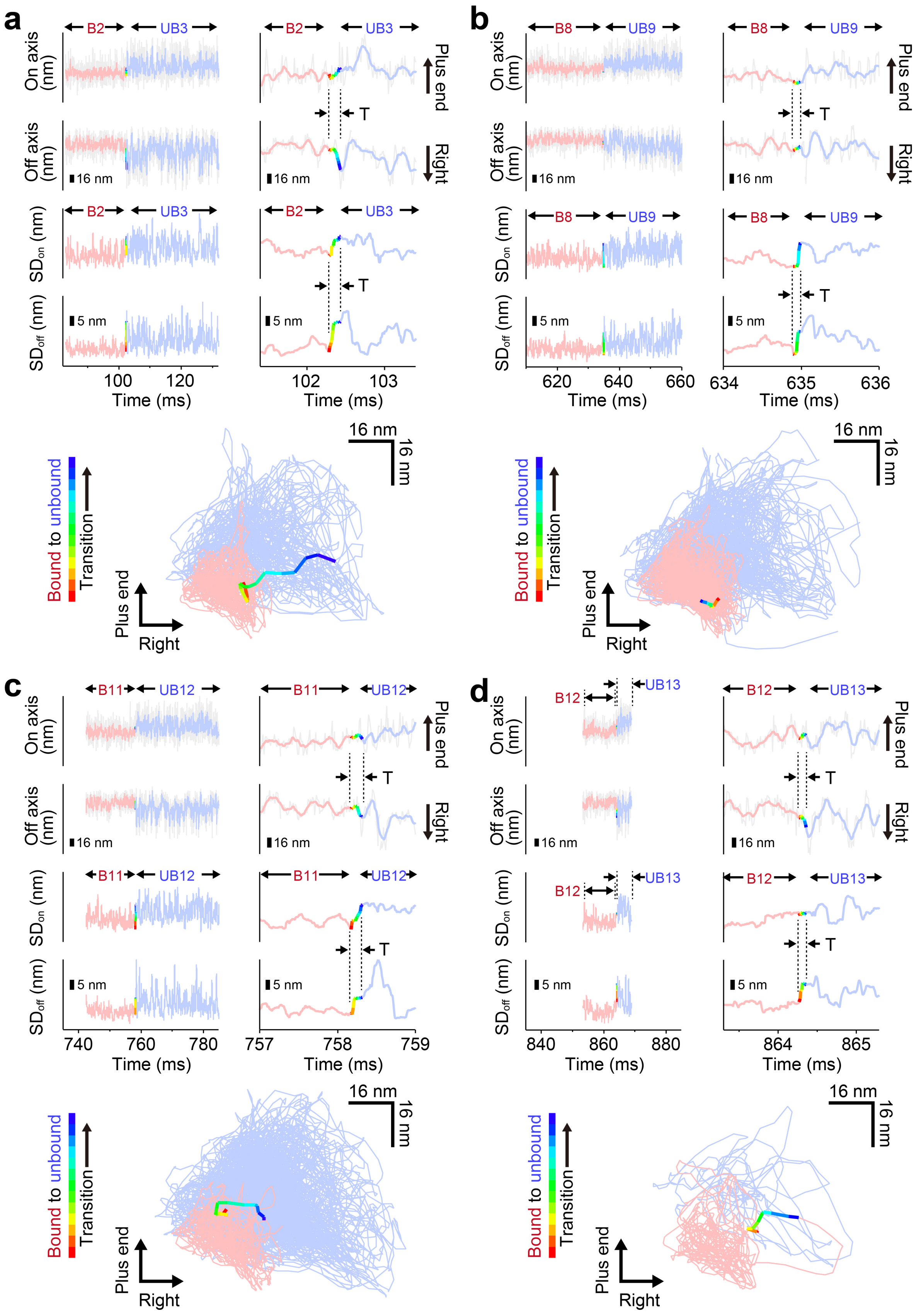

a
B2
UB3
On axis (nm)
Off axis (nm)
16 nm
Plus end
Right
T
SDon (nm)
SDoff (nm)
5 nm
100
120
102
103
Time (ms)
16 nm
Bound to unbound
Transition
Plus end
Right
b
B8
UB9
620
640
660
634
635
636
c
B11
UB12
740
760
780
757
758
759
d
B12
UB13
840
860
880
864
865

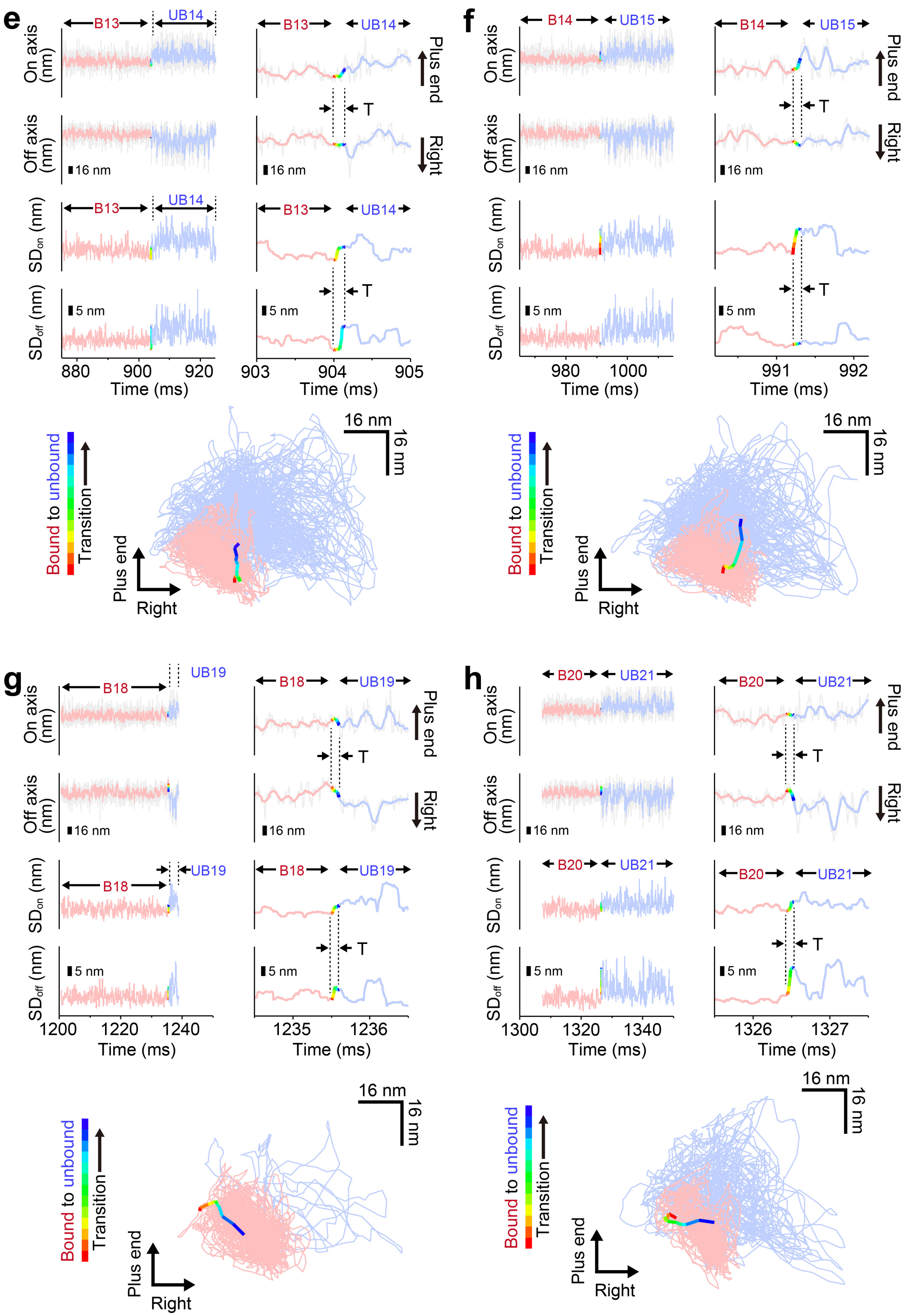

e
f
g
h
B13
UB14
B14
UB15
B18
UB19
B20
UB21
On axis (nm)
Off axis (nm)
SDon (nm)
SDoff (nm)
16 nm
5 nm
T
Plus end
Right
Time (ms)
880
900
920
903
904
905
980
1000
991
992
1200
1220
1240
1235
1236
1300
1320
1340
1326
1327
Bound to unbound
Transition

Fig. S14. Examples of the transition from bound to unbound state of kinesin-1 head labeled with 40 nm AuNP at 10 μs time resolution. (*a-f*) Upper panels show enlarged traces of Fig. 7*a*, showing the center position of AuNP (gray lines) in on- and off-axis, and SD of the position of AuNP for each time (calculated as [t - 10, t + 10]) in on- and off-axis. Numbers correspond to the location of bound and unbound state, shown in Fig. 7*a*. Light red, light blue and rainbow-colored lines represent the filtered trace with moving average (window size of 10 frames) for bound state, unbound state and transition from bound to unbound state, respectively. Lower panel shows two-dimensional plot of the center position of AuNP attached to kinesin-1 head during transition, shown in upper panel. Light red, light blue and rainbow lines represent filtered trace with moving average (window size of 10 frames) for bound state, unbound state and transition from bound to unbound state, respectively.

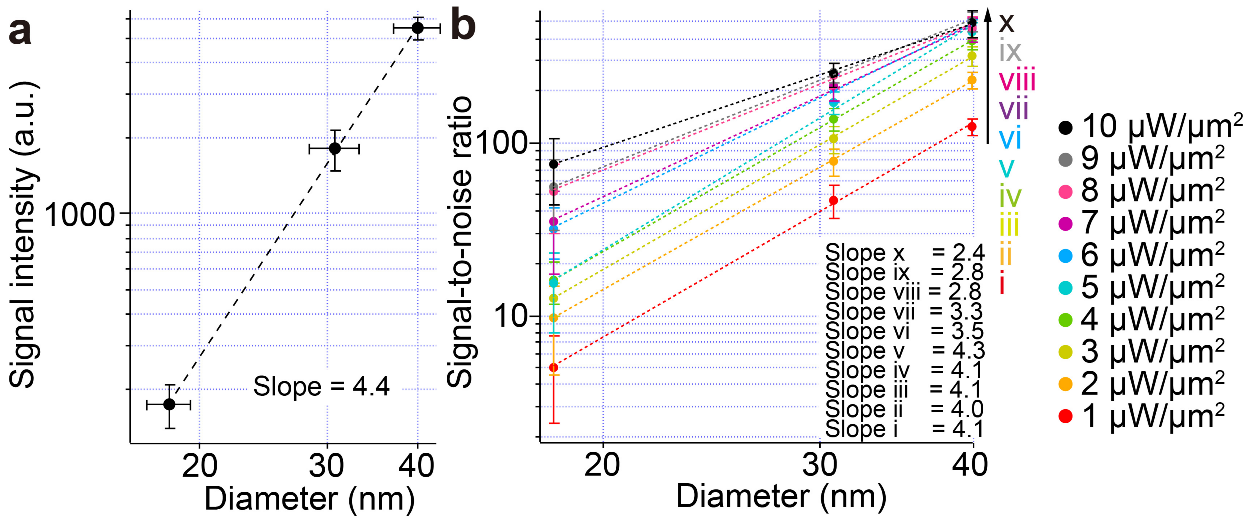

Fig. S15. Dependence of signal intensity and signal-to-noise ratio on particle size. (*a*) A log-log plot between AuNP diameter and scattering signal intensity in the dark-field image (n = 5). Dark-field images were taken at 1 ms time resolution and 4 μW/μm^2 laser intensity. The dotted line shows a linear fit. The slope of the plot was 4.4. (*b*) Log-log plots between AuNP diameter and signal-to-noise-ratio of the dark-field image (n = 5). Dark-field images were taken at 1 ms time resolution and 1–10 μW/μm^2 laser intensity. Dotted lines show linear fits for different laser intensities. The slopes of the dotted lines were 4.1 (i), 4.0 (ii), 4.1 (iii), 4.1 (iv), 4.3 (v), 3.5 (vi), 3.3 (vii), 2.8 (viii), 2.8 (ix), and 2.4 (x), respectively. Note that the error bars for particle diameters were omitted as they are the same size as in (*a*).

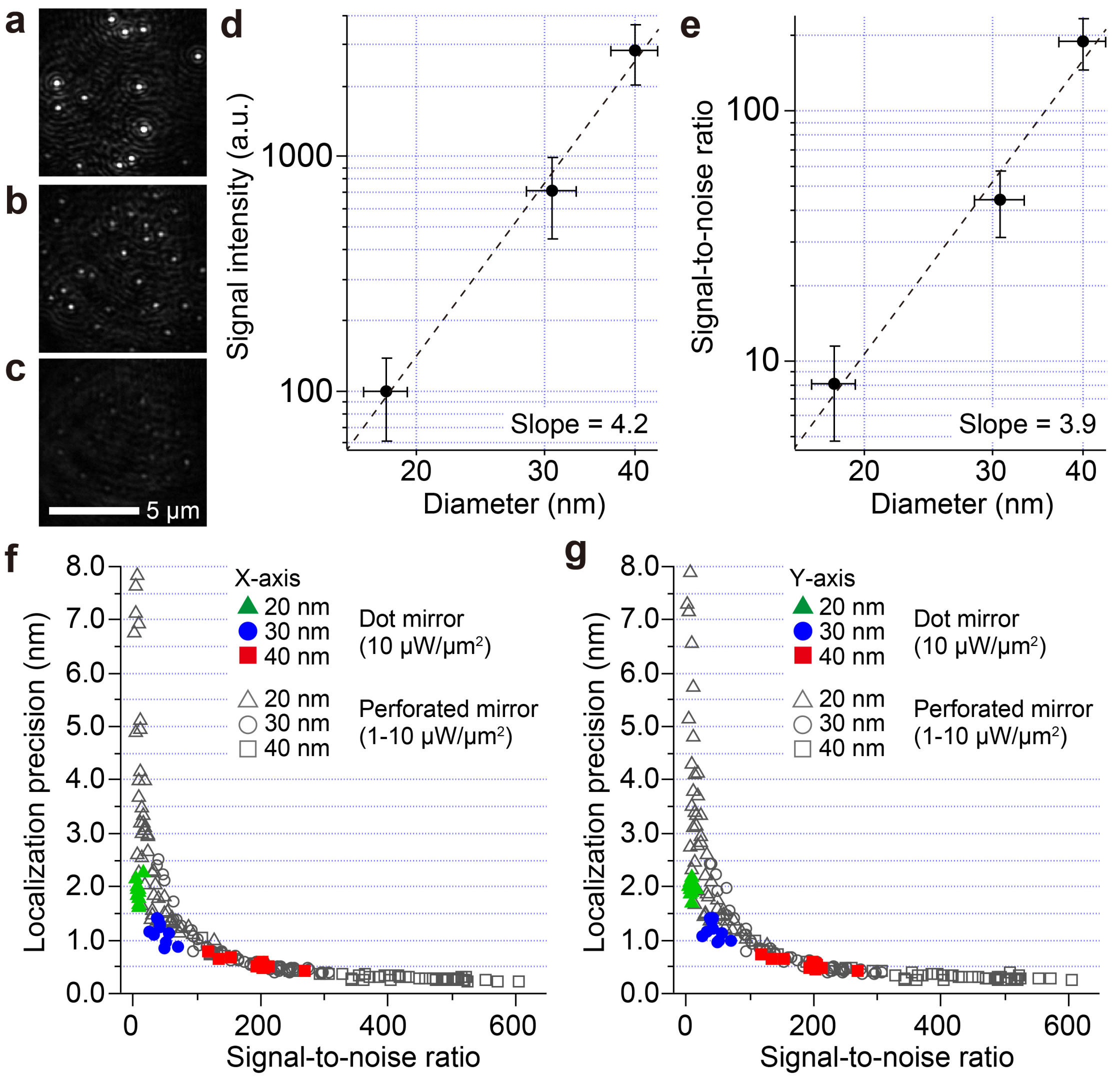

Fig. S16. Dark-field images of AuNPs with vertical illumination dark-field imaging system using dot-mirror, dependence of signal intensity and signal-to-noise ratio on particle size, and relationship between localization precision and signal-to-noise ratio. (*a-c*) Dark-field images of (*a*) 40 nm, (*b*) 30 nm, and (*c*) 20 nm AuNPs, taken at 1 ms time resolution with 10 μW/μm^2 laser intensity. Scale bar is 5 μm. (*d* and *e*) Log-log plots between AuNP diameter and scattering signal intensity (*d*) or signal-to-noise-ratio (*e*) (n = 10). The dotted lines show a linear fit. The slopes of the fit were 4.2 (*d*) and 3.9 (*e*). (*f* and *g*) Relationship between signal-to-noise ratio and localization precision along the (*f*) X- and (*g*) Y-axis. Green triangles, blue circles and red squares represent 20 nm, 30 nm, and 40 nm AuNPs, respectively, observed by vertical

illumination dark-field imaging system using dot-mirror with 10 $\mu W/\mu m^2$ laser intensity. Gray-colored triangles, circles, and squares represent 20 nm, 30 nm, and 40 nm AuNPs observed by total internal reflection dark-field imaging system using perforated mirror with 1- 10 $\mu W/\mu m^2$ laser intensity.

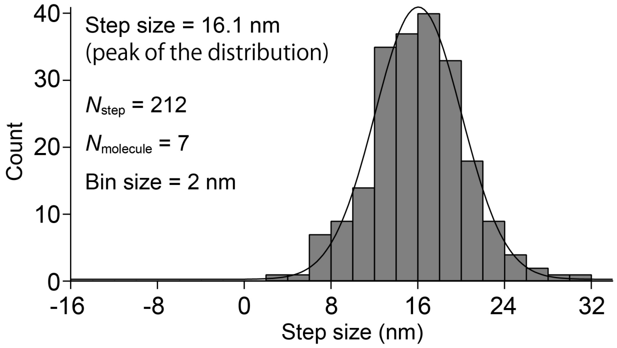

Fig. S17. Distribution of step size of kinesin-1 head labeled with 30 nm AuNP at 50 μs time resolution and at 10 μM ATP. Peak position of the distribution was 16.1 nm, obtained by the Gaussian fit shown as solid line (7 molecules, 212 steps).

Video S1. Video of the Dark-Field Image of 40 nm AuNP Attached to a Kinesin-1 Head at 10 μs Time Resolution and at 10 μM ATP. The movie was replayed at 100 frames per second. Upper side of the movie directs to the plus end of the microtubule. Image size is 0.81 x 0.95 μm.

Video S2. Video of the Dark-Field Image of 30 nm AuNP Attached to a Kinesin-1 Head at 50 μs Time Resolution and at 10 μM ATP. The movie was replayed at 100 frames per second. Upper side of the movie directs to the plus end of the microtubule. Image size is 0.81 x 0.95 μm.
