# Supplementary material for: Single-Nanoparticle Tracking with Angstrom Localization Precision and Microsecond Time Resolution
Source: Biophys J. 2018 Nov 17;115(12):2413–27. doi: 10.1016/j.bpj.2018.11.016 (PMC6302141; doi:10.1016/j.bpj.2018.11.016)
Supplement: Document S2. Article plus Supporting Material [file mmc4.pdf]

Biophysical Journal
Article

Biophysical Society

# Single-Nanoparticle Tracking with Angstrom Localization Precision and Microsecond Time Resolution

Jun Ando,[1,2] Akihiko Nakamura,[1,2] Akasit Visootsat,[1,2] Mayuko Yamamoto,[1] Chihong Song,[3] Kazuyoshi Murata,[3] and Ryota Iino[1,2,*]

[1]Institute for Molecular Science, National Institutes of Natural Sciences, Okazaki, Aichi, Japan; [2]The Graduate University for Advanced Studies, Hayama, Kanagawa, Japan; and [3]National Institute for Physiological Sciences, National Institutes of Natural Sciences, Okazaki, Aichi, Japan

ABSTRACT Gold nanoparticles (AuNPs) have been used as a contrast agent for optical imaging of various single biomolecules. Because AuNPs have high scattering efficiency without photobleaching, biomolecular dynamics have been observed with nanometer localization precision and sub-millisecond time resolution. To understand the working principle of biomolecular motors in greater detail, further improvement of the localization precision and time resolution is necessary. Here, we investigated the lower limit of localization precision achievable with AuNPs and the fundamental law, which determines the localization precision. We first used objective-lens-type total internal reflection dark-field microscopy to obtain a scattering signal from an isolated AuNP. The localization precision was inversely proportional to the square root of the photon number, which is consistent with theoretical estimation. The lower limit of precision for a 40 nm AuNP was ~0.3 nm with 1 ms time resolution and was restricted by detector saturation. To achieve higher localization precision, we designed and constructed an annular illumination total internal reflection dark-field microscopy system with an axicon lens, which can illuminate the AuNPs at high laser intensity without damaging the objective lens. In addition, we used high image magnification to avoid detector saturation. Consequently, we achieved 1.3 Å localization precision for 40 nm AuNPs and 1.9 Å localization precision for 30 nm AuNPs at 1 ms time resolution. Furthermore, even at 33 $\mu$s time resolution, localization precisions at 5.4 Å for 40 nm AuNPs and at 1.7 nm for 30 nm AuNPs were achieved. We then observed motion of head of kinesin-1 labeled with AuNP at microsecond time resolution. Transition cycles of bound/unbound states and tethered diffusion of unbound head during stepping motion on microtubule were clearly captured with higher time resolution or smaller AuNP than those used in previous studies, indicating applicability to single-molecule imaging of biomolecular motors.

## INTRODUCTION

Optical imaging and tracking of single biomolecules have revealed their dynamics in physiological environments. It paves the way for understanding a wide variety of biological phenomena and functions, such as the motion of biomolecular motors for intracellular transportation and the diffusion and assembly of membrane lipids/proteins for signal transduction (1–6). To specifically observe the biomolecules of interest, various optical probes have been developed, such as latex beads (7), organic fluorescent dyes (1,2), fluorescent proteins (3,4), quantum dots (8,9), and gold nanoparticles (AuNPs) (10,11). These materials act as contrast agents for a variety of imaging modalities, such as fluorescence, scattering, differential interference contrast, and absorption (photothermal) microscopy. Above all, single-molecule fluorescence imaging has evolved dramatically in the fields of biophysics and biophotonics ever since the first visualization of a single fluorophore-labeled biomolecule in an aqueous environment (1,2). The motions of single biomolecules are observed by tracking each bright spot in a time course of fluorescence images. In particular, highly sensitive fluorescence detection of single molecules was achieved by evanescent illumination with a total internal reflection configuration, which spatially confines excitation light beneath the surface of the glass substrate with less than 100 nm in penetration depth. The background from the molecules in aqueous solution, which are distant from the glass surface, is effectively suppressed.

*Submitted May 14, 2018, and accepted for publication November 13, 2018.*

*Correspondence: iino@ims.ac.jp

Jun Ando and Akihiko Nakamura contributed equally to this work.

Editor: Julie Biteen.

https://doi.org/10.1016/j.bpj.2018.11.016

© 2018 Biophysical Society.

This is an open access article under the CC BY license (http://creativecommons.org/licenses/by/4.0/).

For single-molecule imaging, the density of the probe-labeled molecules has to be low enough such that they are separated, with a distance larger than a few hundred nanometers. This is because the spatial resolution of optical microscopy is limited to around half the wavelength of light because of diffraction. Although the image of a single probe is spatially spread to a few hundred nanometers in diameter, the central coordinate (position) of the image can be determined at a precision that is better than the diffraction limit (12). Thompson et al. reported that the photon number collected in a single image frame primarily determines the localization precision of the central coordinate, which is inversely proportional to the square root of the number of collected photons (13). Later, localization precision at 1.3 nm was achieved when the photon number was around 14,000 per frame with 0.5 s time resolution; this is so-called fluorescence imaging with one nanometer accuracy (14). By tracking the center of the probe image, stepwise motion of fluorescent-labeled biomolecular motors has been observed with nanometer-scale step size (14–18).

Further improvement in the localization precision and time resolution will directly lead to a deeper understanding of the working principles of biomolecular motors. However, a limited number of photons from fluorescent probes and photobleaching have restricted improvement. Single-molecule analyses of biomolecular motors with angstrom localization precision and sub-millisecond time resolution have been achieved by using a latex-bead probe trapped with optical tweezers to suppress fluctuations of the probe and biomolecules of interest (19–22). In recent years, AuNPs have also been used as probes for free and fast movement of biomolecular motors. An AuNP shows much higher signal intensity than fluorescent dyes without suffering from photobleaching and is much smaller than a latex bead. Dark-field scattering imaging of the AuNP has revealed the motion of biomolecular motors at nanometer localization precision and sub-millisecond time resolution (11,23–27).

Here, we report a detailed analysis of the lower limit of localization precision achievable with AuNPs. The localization precision of the scattering image of AuNP obtained with objective-lens-type total internal reflection dark-field microscopy was investigated by varying the experimental parameters, such as laser intensity, particle size, time resolution, and image pixel size. Based on quantitative analysis, we determined the fundamental laws that determine the localization precision and the factors that restrict its lower limit in this setup. We then designed and developed an annular illumination dark-field microscopy system, which improved the lower limit of localization precision to the angstrom level at microsecond time resolution.

Furthermore, as a proof-of-concept experiment, we applied our imaging system to the observation of stepping motions of kinesin-1. Kinesin-1 is a dimeric motor protein that moves linearly along microtubules for intracellular transport of vesicles and organelles. The two motor domains (heads) of kinesin-1 hydrolyze ATP alternately and move forward in a highly coordinated hand-over-hand manner (15). During linear motion, the trailing head dissociates from the microtubule, passes by the leading head—which is bound to the microtubule—and binds to the tubulin-binding site just ahead of the leading head. The periodicity of dimeric tubulin subunits of the microtubule is 8 nm, and therefore the trailing head moves 16 nm ahead from the original binding site at a time and the net displacement of kinesin-1 relative to the microtubule during a step is 8 nm (15). Recently, high-speed dark-field imaging of kinesin-1 labeled with 40 nm AuNP further revealed the intermediate states during the stepping motion (24). At 55 $\mu$s time resolution and 1.3 nm localization precision, the bound and unbound states of kinesin-1 head on the microtubule were distinguished, in which the kinesin-1 head showed highly diffusive motion during the unbound state. In this study, we have successfully observed movement of AuNP-labeled kinesin-1 with higher time resolution or smaller AuNP than those used in previous studies, indicating applicability to single-molecule imaging of biomolecular motors.

## MATERIALS AND METHODS

### AuNPs

AuNPs with nominal diameters of 20, 30, and 40 nm (EMGC 20, 30, and 40, respectively) were purchased from British Biocell International Solutions (BBI, Crumlin, UK) and were used for experiments. The actual diameters measured with an electron microscope were slightly different from the quoted diameters (Fig. 1).

### Total internal reflection dark-field microscopy with point illumination

A continuous-wave semiconductor laser at 520 nm wavelength (OBIS 520LX; Coherent, Santa Clara, CA) was used as an illumination source. The wavelength matched the plasmon resonance wavelength of AuNPs. The laser beam was introduced into the back port of the inverted microscope body (IX70; Olympus, Tokyo, Japan) and reflected by the peripheral region of the perforated mirror (28). The laser beam was focused with a lens at the peripheral area of the back-focal plane of the oil-immersion objective lens (numerical aperture, NA 1.49; APON 60XOTIRF; Olympus). AuNPs fixed on glass in an aqueous suspension were placed on the sample stage. Total internal reflection occurs at the interface between glass and water and produces an evanescent field to illuminate the AuNPs on glass. The reflected laser beam from the glass-water interface was directed to the opposite side of the peripheral area of the perforated mirror and was reflected to the outside of the microscope body. Scattered light from the AuNPs was collected by same objective lens and was passed through the center portion of the perforated mirror (an elliptical hole with minor and major axes of 7.0 and 9.9 mm, respectively, with circular shape as viewed from the optical axis), to be introduced into the imaging optics. The dark-field scattering image of the AuNPs was recorded with a high-speed complementary metal-oxide-semiconductor (CMOS) camera (Fastcam AX100; Photron, Tokyo, Japan) equipped at the side port of the microscope body through a 5× magnification lens. The image pixel size was 66.7 nm/pixel. The laser power was measured before objective lens, and laser intensity at the sample

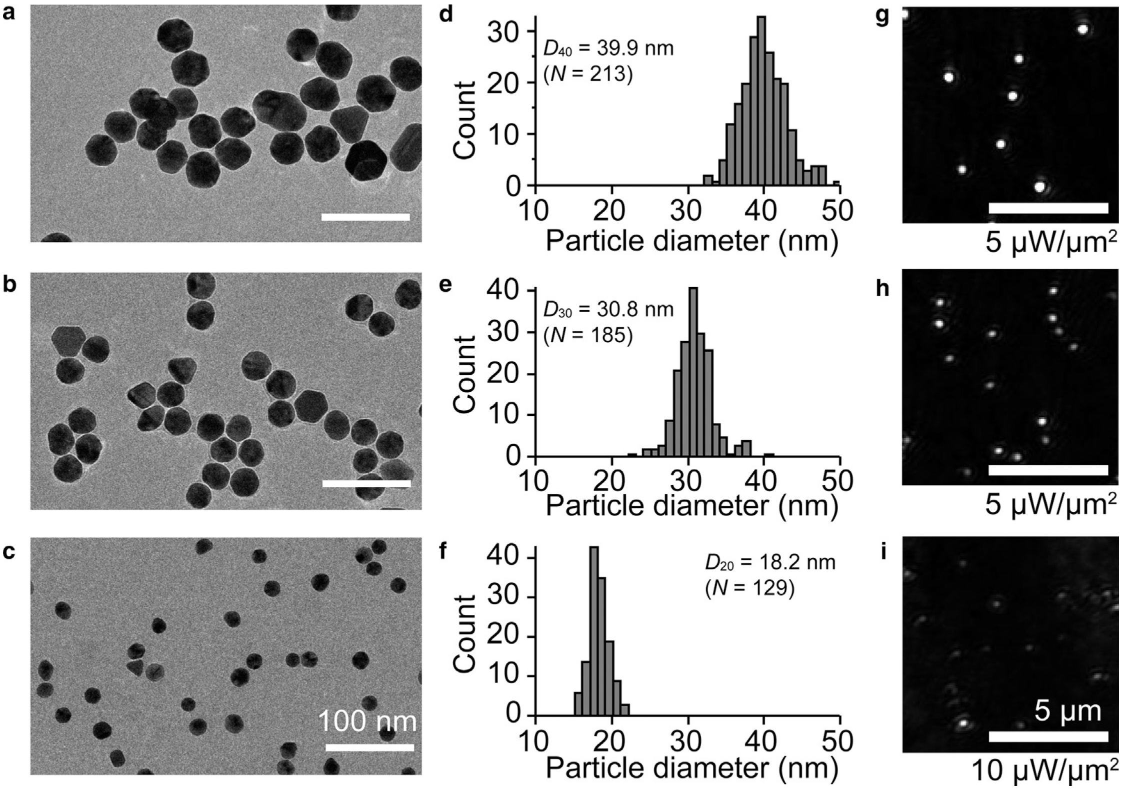


FIGURE 1 Actual diameter of AuNPs used in this study analyzed by electron microscopy, and dark-field images of AuNPs. (*a–c*) Electron microscopic images of (*a*) 40 nm, (*b*) 30 nm, and (*c*) 20 nm AuNPs. Scale bars, 100 nm. (*d–f*) Actual diameter distribution for (*d*) 40 nm, (*e*) 30 nm, and (*f*) 20 nm AuNPs. The actual diameters ($D$) of AuNPs were determined by measuring the cross-sectional area ($A$) in the electron microscopic images and using the equation $A = \pi(D/2)^2$. Note that we estimated the diameter by assuming a spherical shape, although some particles showed triangular, rectangular, and hexagonal shapes. The average particle diameters with SDs, as calculated from the images, were 39.9 $\pm$ 3.0 nm for 40 nm ($n = 213$, $D_{40}$), 30.8 $\pm$ 2.4 nm for 30 nm ($n = 185$, $D_{30}$), and 18.2 $\pm$ 1.3 nm for 20 nm ($n = 129$, $D_{20}$) AuNPs. (*g–i*) Dark-field images of (*g*) 40 nm, (*h*) 30 nm, and (*i*) 20 nm AuNPs. 40 and 30 nm AuNPs were taken with 5 $\mu$W/$\mu$m$^2$ laser intensity. 20 nm AuNPs were taken with 10 $\mu$W/$\mu$m$^2$ laser intensity. The images were taken at 1 ms time resolution. Scale bars, 5 $\mu$m.

plane was calculated as power density ($\mu$W/$\mu$m$^2$), assuming that the objective lens transmittance was 100%.

Sequential dark-field images were obtained to analyze the localization precision of the AuNPs. At each frame, the X-Y coordinates at the center of the AuNP image were calculated using custom-made software (29). The size of the image area for analysis of the center position was 10 $\times$ 10 pixels (667 $\times$ 667 nm). The SD of the distribution of the X or Y coordinate at the center position was defined as the localization precision. For analysis of the AuNP scattering signal intensity, the background intensity was subtracted from the signal intensity. The signal intensity was obtained from the image area that included a single AuNP. Background intensity was obtained from the neighboring area without the AuNP. The size of the image area was 10 $\times$ 10 pixels (667 $\times$ 667 nm) for both signal and background analyses. The stability of the optical system was important to achieve high localization precision. Because the flow of air affected the localization precision, we covered the illumination optics with a box, which blocks air flow around the optical path. This effectively suppressed fluctuations of the central coordinate in the AuNP images.

## Total internal reflection dark-field microscopy with axicon-based annular illumination

The 532 nm laser beam (Excel; Laser Quantum, Stockport, UK) was expanded with a 2.5$\times$ magnification beam expander. The center portion of the expanded beam passed through an iris with $\sim$0.8 mm diameter. The beam was introduced to the center of the axicon lens ($\alpha = 10^\circ$) to form a ring-shaped laser beam. The ring-shaped beam was focused by a lens (f = 100 mm) at the slit portion of the annular mask to eliminate stray light. The inner and outer diameters of the annular mask were 15 and 16 mm, respectively. The beam passed through a quarter-wave plate, iris, and another lens (f = 150 mm) and was then introduced into the side port of the inverted microscope body (IX70; Olympus). The beam was reflected by the peripheral area of the perforated mirror (elliptical hole with minor and major axes of 7.0 and 9.9 mm, respectively, with circular shape as viewed from the optical axis) and focused at the back focal plane of the objective lens with a ring shape. Total internal reflection occurs from all directions at the interface between glass and water and produced an evanescent field to illuminate AuNPs. Scattered light from the AuNPs passed through the center portion of the perforated mirror and was captured with a high-speed CMOS camera (Fastcam AX100; Photron) placed at the side port of the microscope body through a 5$\times$ magnification lens (image pixel size of 67.6 nm/pixel) or through a 10.5$\times$ magnification lens using a pair of lenses (f = 19 mm and f = 200 mm, image pixel size of 31.6 nm/pixel). The laser power was measured before objective lens, and laser intensity at the sample plane was calculated as power density ($\mu$W/$\mu$m$^2$), assuming that the objective lens transmittance was 100%. The distances between the axicon lens and the first lens (f = 100 mm), first lens to annular mask, annular mask to second lens (f = 150 mm), and second lens to perforated mirror were $\sim$140, 100, 440, and 160 mm, respectively. Sequential dark-field images were obtained to analyze the localization precision of the AuNPs. At each frame, the X-Y coordinates at the center of the

AuNP image were calculated using custom-made software (29). The size of the image area for analysis of the center position was 10 × 10 pixels (676 × 676 nm) for 67.6 nm/pixel image pixel size and 20 × 20 pixels (632 × 632 nm) for 31.6 nm/pixel image pixel size. The SD of the distribution of the X or Y coordinate at the center position was defined as the localization precision.

## Dark-field microscopy with vertical illumination

A continuous-wave semiconductor laser at 520 nm wavelength (OBIS 520LX; Coherent) was used as an illumination source. The laser beam was introduced into the back port of the inverted microscope body (IX70; Olympus) and reflected by the central region of a dot mirror (an elliptical dot with minor and major axes of 4.0 and 5.7 mm, respectively, with circular shape as viewed from the optical axis) (28). The laser beam was focused with a lens at the center portion of the back-focal plane of the oil-immersion objective lens (NA 1.40, PLAPON 60XOSC2; Olympus). AuNPs fixed on glass in an aqueous suspension were placed on the sample stage. Scattered light from the AuNPs was collected by the same objective lens and was passed through the peripheral portion of the dot mirror to be introduced into the imaging optics. The dark-field scattering image of the AuNPs was recorded with a high-speed CMOS camera (Fastcam AX100; Photron) equipped at the side port of the microscope body through 5× magnification lens. The image pixel size was 66.4 nm/pixel. The laser power was measured before the objective lens, and laser intensity at the sample plane was calculated as power density ($\mu W/\mu m^2$), assuming that the objective lens transmittance was 100%.

Sequential dark-field images were obtained to analyze the localization precision of the AuNPs. At each frame, the X-Y coordinates at the center of the AuNP image were calculated using custom-made software (29). The size of the image area for analysis of the center position was 10 × 10 pixels (664 × 664 nm). The SD of the distribution of the X or Y coordinate at the center position was defined as the localization precision. For analysis of the AuNP scattering signal intensity, the background intensity was subtracted from the signal intensity. The signal intensity was obtained from the image area that included a single AuNP. Background intensity was obtained from the dark-field image of the control sample without AuNP. The size of the image area was 10 × 10 pixels (664 × 664 nm) for both signal and background analyses.

## Electron microscopic observation

AuNPs were observed with an electron microscope to analyze their actual size distributions, as shown in Fig. 1, *a–f*. A drop of the AuNP suspension was placed on a glow-discharged carbon-coated copper grid and dried in air. The AuNPs on the grid were examined using an electron microscope (JEM 2200FS; JEOL, Tokyo, Japan) with a field-emission electron source operating at 200 kV and an in-column ($\Omega$-type) energy filter operating in zero-energy-loss mode and with ~15 eV slit width. The images were recorded with a direct-detector CMOS camera (DE20; Direct Electron LP, San Diego, CA) with 32,715× detector magnification, which corresponds to 2.0 Å per pixel. The actual diameter ($D$) of the AuNPs in the electron microscope images was calculated using the cross-sectional area ($A$) of the isolated particles in the image. We used the equation $A = \pi(D/2)^2$ assuming spherical particles, although some nanoparticles showed triangular, rectangular, and hexagonal shapes. The average particle diameter and the SD calculated from the images were 39.9 ± 3.0 nm for 40 nm ($n = 213$), 30.8 ± 2.4 nm for 30 nm ($n = 185$), and 18.2 ± 1.3 nm for 20 nm ($n = 129$) AuNPs.

## Total internal reflection fluorescence microscopy

A continuous-wave semiconductor laser with 532 nm wavelength (DPGL-2100F; Photop, Shanghai, China) was used as the excitation source. The laser beam was introduced to the rotating diffractive diffuser (30,31) for annular illumination. Zero-order diffraction was blocked by a mask, and the remaining ring-shaped beam was focused with a lens at the slit portion of the annular mask to eliminate stray light. The beam passing through the mask was introduced into another lens and then into the side port of the inverted microscope body (IX71; Olympus), reflected by a dichroic mirror, and focused at the back-focal plane of the oil-immersion objective lens (NA 1.45, PlanAPO TIRFM; Olympus) with a ring shape. The fluorescence signal, which was collected by the same objective lens, passed through the dichroic mirror and a barrier filter and was detected with an EMCCD (electron multiplying charge coupled device) camera (iXonUltra888; Andor, Belfast, UK) placed at the side port of the microscope.

To determine localization precision of a fluorescent dye, Cy3, we observed Cy3-labeled chitinase A (D415C mutant for Cy3-labeling) with $His_6$-tag (50 pM in final) (31). The Cy3-labeled chitinase A molecules were fixed on the glass surface in 50 mM sodium phosphate buffer at pH 6.0 and were observed at 0.28 $\mu W/\mu m^2$ laser intensity. Sequential images were obtained at 1 s time resolution for 30 s (30 frames in total). The electron multiplying-gain was 300, and the temperature of the sensor was −80°C. The pixel size of the image was 67.7 nm/pixel. The center coordinate of the fluorescence image of single Cy3-labeled chitinase A was calculated by two-dimensional Gaussian fitting. The localization precision of the image was obtained from the SD of the distribution of the X or Y coordinate at the center of the image. The photon number for each image was calculated according to the detector count and gain setting.

## Purification and biotinylation of kinesin-1

Cysteine residue (S55C) was introduced into the cysteine-light human kinesin-1 heterodimer, as reported previously (24). One of the polypeptide chains in the heterodimer has a Strep-tag at the C-terminus. Another polypeptide chain contains a $His_6$-tag at the C-terminus and cysteine residue (S55C) at motor domain. Heterodimers were expressed in *Escherichia coli* Tuner (DE3) and purified by nickel nitrilotriacetic acid affinity chromatography, and subsequently purified by Strep-Tactin affinity chromatography. The obtained sample was further purified by gel filtration chromatography. The cysteine residue of purified kinesin-1 was reduced by 1 mM dithiothreitol for 30 min on ice. After incubation, the dithiothreitol was removed by NAP column (NAP-5; GE Healthcare, Chicago, IL). The reduced sample was immediately mixed with biotin-$PEAC_5$-maleimide (B299; Dojindo, Kumamoto, Japan) at a molar ratio of 1:6 (kinesin-1 dimer/biotin-$PEAC_5$-maleimide) for 60 min on ice. After incubation, unreacted biotin-$PEAC_5$-maleimide was removed by NAP column (NAP-10; GE Healthcare).

## Surface modification of AuNP with streptavidin and labeling of biotinylated kinesin-1

Colloidal suspension of 40 nm AuNPs (EMGC40; BBI Solutions) or 30 nm AuNPs (EMGC30; BBI Solutions) was modified with streptavidin, as described previously with small modification (24). They were suspended in 10 mM phosphate buffer (pH 8.0) with 0.2% Tween 20. To form biotin-functionalized surface on AuNP, alkanethiol mixture solution was added at a final concentration of 0.1 mg/mL biotinylated alkanePEG thiol (SPT-0012D; SensoPath Technologies, Bozeman, MT), 0.2% carboxy-EG6-undecanethiol (C445; Dojindo), and 0.2% hydroxy-EG6-undecanethiol (H355; Dojindo). After incubation for 6 h at 70°C, unreacted alkanethiols were removed by centrifugation (10,000 × $g$, 3 min for 40 nm AuNP and 5 min for 30 nm AuNP) and mixed with streptavidin (PRO-791-b; Prospec, Fullerton, CA) at a final concentration of 0.67 mg/mL, dissolved in 10 mM phosphate buffer (pH 8.0) with 0.2% Tween 20. The solution was gently mixed using a rotator for 3 h at room temperature, and unreacted streptavidin was removed by centrifugation (10,000 × $g$, 3 min for 40 nm AuNP and 5 min for 30 nm AuNP). The pellet was suspended in 10 mM phosphate

buffer (pH 8.0) and stored in a refrigerator at 4°C. Just before starting measurement, biotinylated kinesin-1 and streptavidin-coated AuNP were mixed at a ratio of 2 (kinesin-1 molecules/AuNP particles).

## Preparation of microtubule and construction of flow cell

Tubulin was purified from pig brain and stored at −80°C at 6 mg/mL. Microtubules were obtained by polymerizing tubulin at 37°C for 30 min in the presence of 0.75 mM GTP. After polymerization, the solution was centrifuged (72,000 rotations per minute, 30 min), and the supernatant was removed. The pellet was dissolved in the BRB80 buffer (80 mM PIPES, 1 mM $MgCl_2$, 1 mM EGTA (pH 6.8)) with 1 mM GTP and 20 $\mu$M taxol. Before observation, the microtubule solution was ~5 times diluted by BRB80 buffer containing 20 $\mu$M taxol. For microscopic observation, the microtubules in solution were fixed on the glass surface of the flow cell. The flow cell was constructed with two cover glasses (18 × 18 mm for upper layer and 24 × 32 mm for bottom layer; Matsunami Glass, Osaka, Japan). The cover glass for the bottom layer was washed with ethanol and 10 M KOH. To modify the surface of the cleaned cover glass, it was rinsed with acetone, reacted with 0.2% (3-aminopropyl)triethoxysilane (LS-3150; Shin-Etsu Silicone, Tokyo, Japan) in acetone for 60 min, and washed with acetone, ethanol, and water.

After surface coating of the cover glass for the bottom layer of the flow cell, another cover glass for the upper layer was attached to it by grease with thin spacers to form three flow cells in parallel. The volume of each flow cell was around 10 $\mu$L. In each flow cell, 10 $\mu$L of 8% glutaraldehyde (072-02262; Wako, Osaka, Japan) in water was immersed for 20 min and washed with 60 $\mu$L of water. 10 $\mu$L of microtubule solution was immersed in the flow cell for 2 min to fix them on the glass surface. The flow cell was then washed with 20 $\mu$L of BRB80 buffer containing 20 $\mu$M taxol. To prevent nonspecific binding of kinesin-1 on the glass surface, the flow cell was infused with 20 $\mu$L of 1 mg/mL bovine serum albumin (010-15153; Wako) in BRB80 buffer containing 20 $\mu$M taxol for 2 min. The remaining bovine serum albumin was removed by 20 $\mu$L of the observation buffer (BRB80 buffer containing 1 mg/mL casein, 2.5 mM phosphoenolpyruvate, 0.1 mg/mL pyruvate kinase, and 10 $\mu$M ATP). The flow cell was immersed with 10 $\mu$L of AuNP-labeled kinesin-1 in observation buffer, and the observation was performed at 25 ± 1°C.

## Dark-field imaging of AuNP-labeled kinesin-1 with axicon-based annular illumination system

Annular illumination dark-field imaging system, mentioned above, was used for observation of kinesin-1. The actual image pixel size of the detector (Fastcam AX100; Photron) was 67.6 nm/pixel with a 5× magnification lens. The center coordinate of the dark-field image of single-AuNP-labeled kinesin-1 was calculated by two-dimensional Gaussian fitting. For Figs. 7 and S14, time resolution was 10 $\mu$s with a laser intensity of 40 $\mu W/\mu m^2$. For Figs. 8 and S17, time resolution was 50 $\mu$s with a laser intensity of 40 $\mu W/\mu m^2$.

## Data analysis of kinesin-1

At each frame of the image sequence, the center coordinate of the AuNP attached to kinesin-1 was obtained by two-dimensional Gaussian fit to the intensity profile of the AuNP image using same algorithm reported previously (24,28). The obtained X-Y coordinates ($X_{raw}$ and $Y_{raw}$) were adjusted to the orientation parallel to the microtubule long axis (on axis, $X_{on}$) and the orientation perpendicular to the microtubule long axis (off axis, $Y_{off}$) (24). The coordinates of raw data, $X_{raw}$ and $Y_{raw}$, were rotated to $X_{on}$ and $Y_{off}$ by the following:

$$X_{on} = \cos\theta \times X_{raw} + \sin\theta \times Y_{raw} \quad (1)$$

and

$$Y_{off} = -\sin\theta \times X_{raw} + \cos\theta \times Y_{raw}, \quad (2)$$

where $\theta$ is the angle between $X_{on}$ axis and $X_{raw}$ axis. Velocity of the kinesin-1 motion was obtained by the slope of the linear fit of $X_{on}$ plot with time. The SD of on- and off-axis position was calculated for each time frame t. The window size was t ± 10 frames for Figs. 7 and S14 and was t ± 20 frames for Fig. 8. For the analysis of step size shown in Fig. S17, the trajectory ($X_{on}$, $Y_{off}$) was treated with median filtering (window size of 30 frames) to reduce the noise. The steps in the median-filtered on-axis trajectory were identified by the algorithm developed by Kerssemakers et al. (32). The unbound state, the bound state, and their transition of AuNP-labeled kinesin-1 head on the microtubule—shown in Figs. 7, 8, and S14—were distinguished by eye based on the SD in the off-axis position, where high SD represents unbound state, low SD represents bound state, and the transition from low SD to high SD represents the bound-to-unbound state transition. For the plot of $X_{on}$, $Y_{off}$, $SD_{on}$ and $SD_{off}$ with time, and the two-dimensional plot of $X_{on}$ and $Y_{off}$, moving average with the window size of 10 frames were used to reduce the noise.

## RESULTS AND DISCUSSION

Imaging of single AuNPs was performed with a custom-made, objective-lens-type total internal reflection dark-field microscopy system using a perforated mirror, as described in the Materials and Methods (28). First, the localization precision of the AuNPs was analyzed at different laser intensities. AuNPs with diameters of 20, 30, and 40 nm were used for observation. Note that the diameters described above are nominal values provided by the manufacturer, and actual values are slightly different (Fig. 1, *a–f*). AuNPs were fixed on cover glass with a high ionic strength aqueous solution and observed (Fig. 1, *g–i*). Sequential images were obtained with 1 ms time resolution (1000 frames per second) for a duration of 0.5 s (500 frames in total). The two-dimensional coordinates of the center position of the AuNP image at each frame were calculated using custom-made software (Figs. S1–S4) (29). The SD of the distribution of the X or Y coordinate of the center position was defined as the localization precision. Fig. S5, *a* and *b* show the localization precision at different laser intensities along the X and Y axes. The value of localization precision decreased with increased laser intensity and with increased particle size. For the 40 nm AuNP, the localization precision saturated when the laser intensity was more than 5 $\mu W/\mu m^2$. The lower limit of localization precision achieved in this measurement was around 0.3 nm.

Then, we calculated the signal, noise, and signal/noise ratio (S/N) of the images using Eq. 3:

$$S/N = \frac{I_s - I_b}{\sqrt{\sigma_s^2 + \sigma_b^2}}. \quad (3)$$

$I_s$ and $I_b$ are the signal and background intensities, respectively, and ${\sigma_s}^2$ and ${\sigma_b}^2$ are their variances (33,34). The signal

intensity was obtained from the image area that includes a single AuNP, whereas the background intensity was obtained from the neighboring area without an AuNP. The size of the image area was 10 × 10 pixels (667 × 667 nm) for both signal and background analyses. Fig. S5 *c* shows the signal and noise at different laser intensities. As the laser intensity increased, the noise did not change in a large way, whereas the signal increased. Fig. S5 *d* shows the S/N at different laser intensities. The S/N increased with increased laser intensity and with increased particle size. For the 40 nm AuNP, the S/N was saturated at around 500 when the laser intensity was higher than 5 $\mu$W/$\mu$m$^2$. The saturation occurred at nearly the same laser intensity for both localization precision and S/N. Those results indicate a strong correlation between localization precision and S/N. Fig. 2, *a* and *b* show the relationship between the S/N and localization precision of the AuNP. The value of localization precision decreased exponentially with increased S/N. Regardless of particle size, the localization precision of the AuNP image was basically governed by its S/N. Thompson et al. described the localization precision ($\sigma$) by

$$\sigma = \sqrt{\left(\frac{s^2}{N} + \frac{a^2/12}{N} + \frac{8\pi s^4 b^2}{a^2 N^2}\right)}, \tag{4}$$

where $N$, $s$, $a$, and $b$ are the number of photons collected in a single image frame, the width (SD) of the image, the image pixel size, and the SD of the background, respectively (13). When the photon number is high enough, the first and second terms in Eq. 4 become dominant (14). The localization precision is therefore expected to be inversely proportional to the square root of the photon number. Based on Eq. 4, photon numbers were back calculated from the localization precision and the obtained experimental parameters. The width of each image was obtained by the two-dimensional Gaussian fitting. Fig. 2, *c* and *d* show a log-log plot of the dependence of the photon number on the S/N, in which the slopes below 50,000 photons were 1.1 along both the X and Y axes, indicating linear relationship. Fig. 2, *e* and *f* show the relationship between the estimated photon number and the localization precision. The localization precision decreased nonlinearly with increased photon number, regardless of particle size. When the photon number was smaller than 50,000, the slopes of the linear fit in the log-log plot were −0.56 and −0.55 along the X and Y axes, respectively. As is expected from Eq. 4, localization precision was inversely proportional to the square root of the photon number. On the other hand, when the photon number was larger than 50,000, the slopes became less steep, for which the linear fits in the log-log plot were −0.35 and −0.23 along the X and Y axes, respectively. In this high photon number region, the localization precision deviated from Eq. 4. The lower limit of precision was restricted to ~0.3 nm owing to the change in slope. We suppose that the slope change was caused by detector saturation. This is because the point-spread function of the particle image was deformed when the photon number was roughly higher than 50,000, as shown in Fig. S6. Line profile of the image could not be fitted with Gaussian at high photon number with high laser intensity because of the saturation of the image. The value of the $s$ therefore increased with increase of the laser intensity (Fig. S7). On the other hand, the value of the $b$ did not change by a large amount (Fig. S7). The distribution of $b$, generated from the time course of the image, was well fitted by a Gaussian (Fig. S8), indicating the background fluctuation is described by the Poisson noise. These results suggest that not background fluctuations, but incorrect values of the $s$ affect the deviation from Eq. 4. Indeed, when the fixed value of the $s$, obtained at the laser intensity of 1 $\mu$W/$\mu$m$^2$, was used for the back calculation of the photon number, the slope followed Eq. 4 without saturation (Fig. 2, *e* and *f*). Thus, in this setup, we concluded that further improvement of the localization precision is restricted by detector saturation, owing to the limited well depth of the image sensor. Development of the image sensor with a high well depth will contribute to an improved localization precision. Furthermore, a combination of higher image magnification (smaller image pixel size) and higher laser intensity than this setup will improve the localization precision, although a very large laser intensity would damage the objective lens. This point will be examined in detail later. The experimental value of the localization precision of a fluorescent dye, Cy3 ($n$ = 5, *black filled circle*) and the theoretical localization precision of Cy3 based on Eq. 4 and the obtained experimental parameters ($n$ = 5, *black open circle*) are also shown for comparison. As shown in Figs. S5 c and S7, noise and SD of the background ($b$ in Eq. 4) did not change largely with increased laser intensity, whereas the signal (and the photon number $N$ in Eq. 4) increased and resulted in better localization precision. These results indicate that the noise of the system, including the electronic noise of the detector and the variations among the pixels, will not significantly affect the measurement at sub-nm localization precision. As a biological application of sub-nm localization precision, we have recently used 40 nm AuNP to probe linear motion of *Serratia marcescens* chitinase A, which moves on the crystalline chitin (35). One nm forward and backward stepping motions have been successfully observed with 0.3 nm localization precision at 0.5 ms time resolution, at which the 1 nm step size corresponds to the length of hydrolysis reaction product, chitobiose.

With the experimental setup described above, the lower limit of localization precision was around 0.3 nm for 40 nm AuNPs, and the combination of higher laser intensity and higher image magnification (smaller image pixel size) than this setup will improve the localization precision. However, the optical system described above has a limitation on

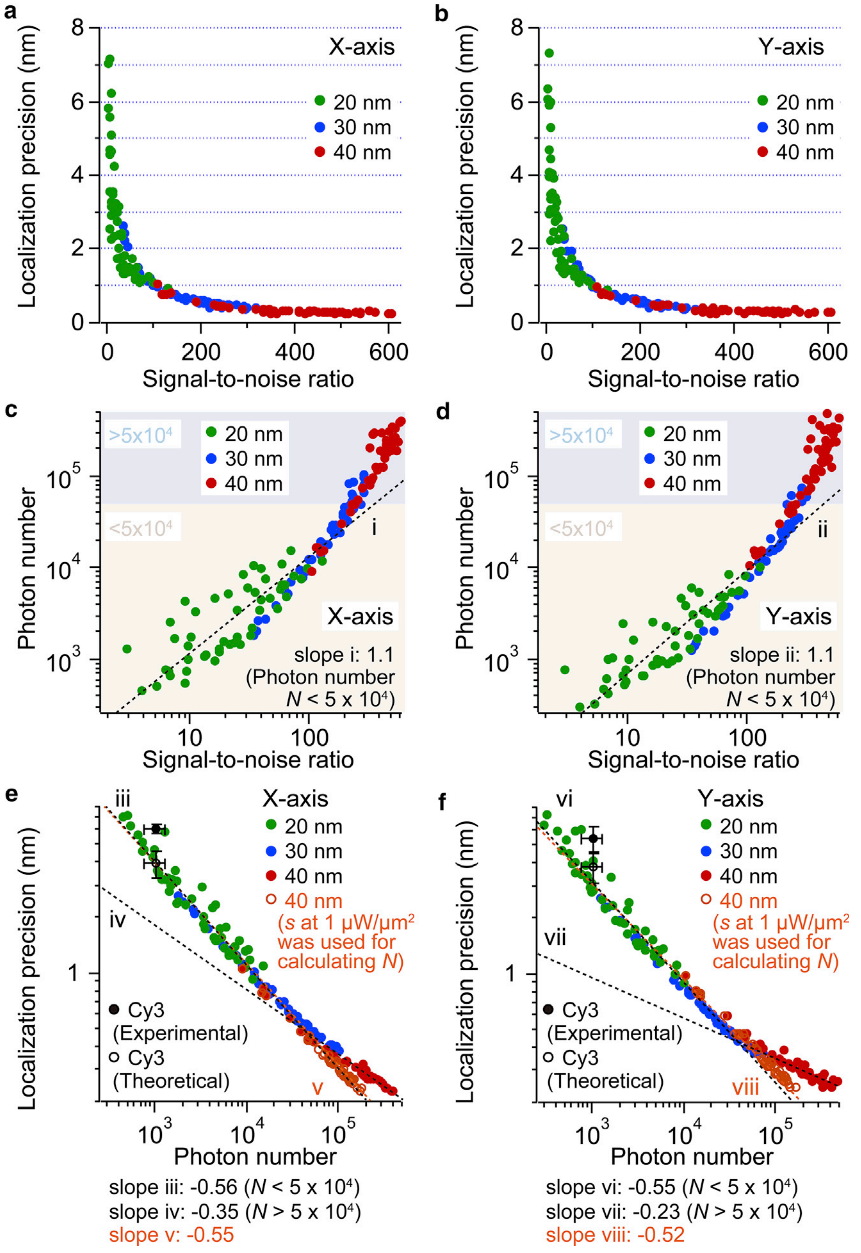


FIGURE 2 Relationships among localization precision, S/N, and photon number of the dark-field image of 20 nm (*green*), 30 nm (*blue*), and 40 nm (*red*) AuNPs. (*a* and *b*) Relationships between S/N and localization precision along the (*a*) X and (*b*) Y axes. (*c* and *d*) Log-log plots between S/N and photon number along the (*c*) X and (*d*) Y axes ($n = 5$). Dotted lines represent linear fits applied to data with less than 50,000 photons. Slopes of the dotted lines are 1.1 (*i*) and 1.1 (*ii*), respectively. (*e* and *f*) Log-log plots between estimated photon number and localization precision along the (*e*) X and (*f*) Y axes. Orange symbols represent 40 nm AuNPs for which the photon number was calculated with the $s$ at 1 $\mu W/\mu m^2$ laser intensity. Dotted black lines represent linear fits applied to data below and above 50,000 photons. The dotted orange line represents a linear fit applied to data with the photon number calculation with the $s$ at 1 $\mu W/\mu m^2$ laser intensity. The slopes of the dotted lines are −0.56 (*iii*), −0.35 (*iv*), −0.55 (*v*), −0.55 (*vi*), −0.23 (*vii*), and −0.52 (*viii*), respectively. Filled and open black circles represent experimental and theoretical values for single fluorophore (Cy3), respectively ($n = 5$). All error bars represent SD. To see this figure in color, go online.

the incident laser power because the laser beam is tightly focused on one point at the back-focal plane of the objective lens. High power density at the focal point damages the objective lens. Therefore, we designed and constructed an annular illumination total internal reflection dark-field microscopy system with an axicon lens. Fig. 3 *a* shows a schematic illustration of the optical system. Details are summarized in the Materials and Methods. In this configuration, a ring-shaped laser beam is focused at the back-focal plane of the objective lens. Therefore, we can effectively disperse the laser power at the back-focal plane, resulting in an improved damage threshold for the incident laser power.

Then, we observed 40 and 30 nm AuNPs with the high laser intensity achieved by the annular illumination with small image pixel size (Fig. 3, *b–e*). We compared the results obtained with the image pixel size of 31.6 nm/pixel and 67.6 nm/pixel (Figs. S9–S12). The laser intensity dependence of the localization precision and S/N of 40 nm AuNPs at 1 ms time resolution is shown in Fig. 4, *a–c*. For both image pixel sizes, localization precision and S/N were improved with increased laser intensity. With an image pixel size of 67.6 nm/pixel, the S/N showed saturation when the laser intensity was more than 5 $\mu W/\mu m^2$, and the lower limit of localization precision was restricted to ~0.2 nm. On the other hand, with the image pixel size

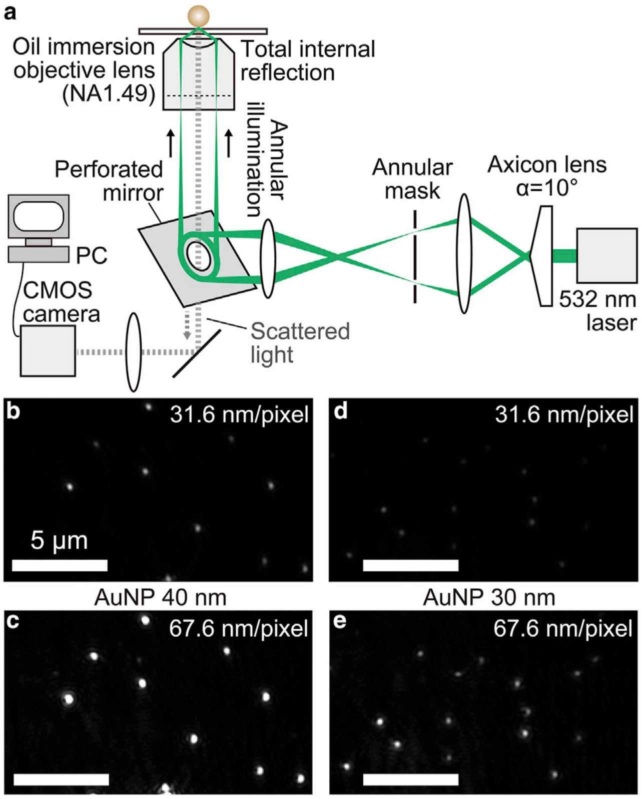


FIGURE 3 Axicon-based annular illumination system for dark-field imaging of AuNPs. (*a*) An optical schematic for annular illumination total internal reflection dark-field microscopy using an axicon lens. The details are described in the Materials and Methods. (*b–e*) Dark-field images of 40 nm AuNPs with image pixel sizes of (*b*) 31.6 nm/pixel and (*c*) 67.6 nm/pixel and those of 30 nm AuNPs with image pixel sizes of (*d*) 31.6 nm/pixel and (*e*) 67.6 nm/pixel. The same image fields were observed for (*b*) and (*c*) and (*d*) and (*e*), respectively. The images were taken at 1 ms time resolution. Scale bars, 5 $\mu$m. To see this figure in color, go online.

of 31.6 nm/pixel, the value of the localization precision decreased with a slight change in slope up to a laser intensity of ~20 $\mu$W/$\mu$m$^2$. As a result, we achieved 1.3 Å localization precision at 30 $\mu$W/$\mu$m$^2$ laser intensity. In Fig. 4, *a–c*, the dependence of the localization precision and the S/N on the laser intensity showed a crossover around 10 $\mu$W/$\mu$m$^2$ between two image pixel sizes. With a smaller pixel size at low laser intensity, the S/N and localization precision degraded. On the other hand, when the laser intensity increased, the signal intensity of the image with larger pixel size shows saturation earlier than that with smaller pixel size. Therefore, at more than 20 $\mu$W/$\mu$m$^2$ laser intensity, the S/N and localization precision of the image with smaller pixel size improved and reached 1000 and 1.3 Å, respectively. Furthermore, we also compared the localization precision, signal, noise, and S/N between the annular illumination and point-illumination systems (Fig. S13). Under the conditions with similar pixel sizes, 66.7 and 67.6 nm/pixel for point-illumination- and axicon-based systems, respectively, there were no significant differences. Our results clearly show that high localization precision can be achieved with high laser intensity and small image pixel size. However, the image pixel size must be carefully chosen based on the localization precision required for each measurement because field of view will be limited at high image magnification. We also performed the same measurement with 30 nm AuNPs. Fig. 4, *d–f* show the dependence of the localization precision and S/N on the laser intensity. The lower limits of localization precision for 30 nm AuNPs were 1.9 and 2.1 Å with 31.6 and 67.6 nm/pixel image pixel sizes, respectively, at the laser intensity of 40 $\mu$W/$\mu$m$^2$.

Fig. 5 shows the relationship between the estimated photon numbers and localization precision as calculated from dark-field images of the 40 and 30 nm AuNPs with image pixel sizes of 67.6 and 31.6 nm/pixel. With larger pixel size, the slopes of the linear fit in the log-log plot for the X and Y axes were −0.51 and −0.50, respectively, until the photon number reached 60,000, whereas they were −0.20 and −0.23, respectively, when the photon number was larger than 60,000. On the other hand, with smaller pixel size, the slopes of the linear fit in the log-log plot in the X and Y axes were −0.55 and −0.56, respectively, until the photon number was around 200,000. The slopes become −0.23 and −0.29, respectively, when the photon number was larger than 200,000. The inflection points of the slope in the log-log plot correspond to the onset of detector saturation, as discussed in the previous section. Detector saturation was effectively avoided with smaller pixel size. Furthermore, when the fixed value of the *s* obtained at the laser intensity of 1 $\mu$W/$\mu$m$^2$ was used for the back calculation of the photon number as in Fig. 2, *e* and *f*, the slope followed Eq. 4 without saturation. When the image pixel size reduces by a factor of 2, pixel numbers in the same image area increase by a factor of 4, resulting in an increased photon number by a factor of 4 collected before detector saturation. By using small pixel size and high laser intensity, detector saturation was effectively suppressed while large numbers of photons (~10$^6$) were collected. As a result, the localization precision improved to the angstrom level.

Annular illumination total internal reflection fluorescence microscopy with a rotating mirror or a diffractive diffuser has been reported previously (30,31,36). Our axicon-lens-based system is similar to the diffractive-diffuser-based system but has an advantage in terms of illumination laser intensity. In a diffractive-diffuser-based system, first-order diffraction of the laser is used for illumination, and the zero-order light is blocked with a beam damper, resulting in significant loss of laser power due to low diffraction efficiency. On the other hand, in the axicon-based system, all portions of the beam that entered the axicon lens can be used for illumination. In addition to the availability of high laser intensity, annular illumination has an advantage over point illumination because of polarization of the evanescent field (30,31,36). The evanescent field formed by total internal reflection microscopy with point

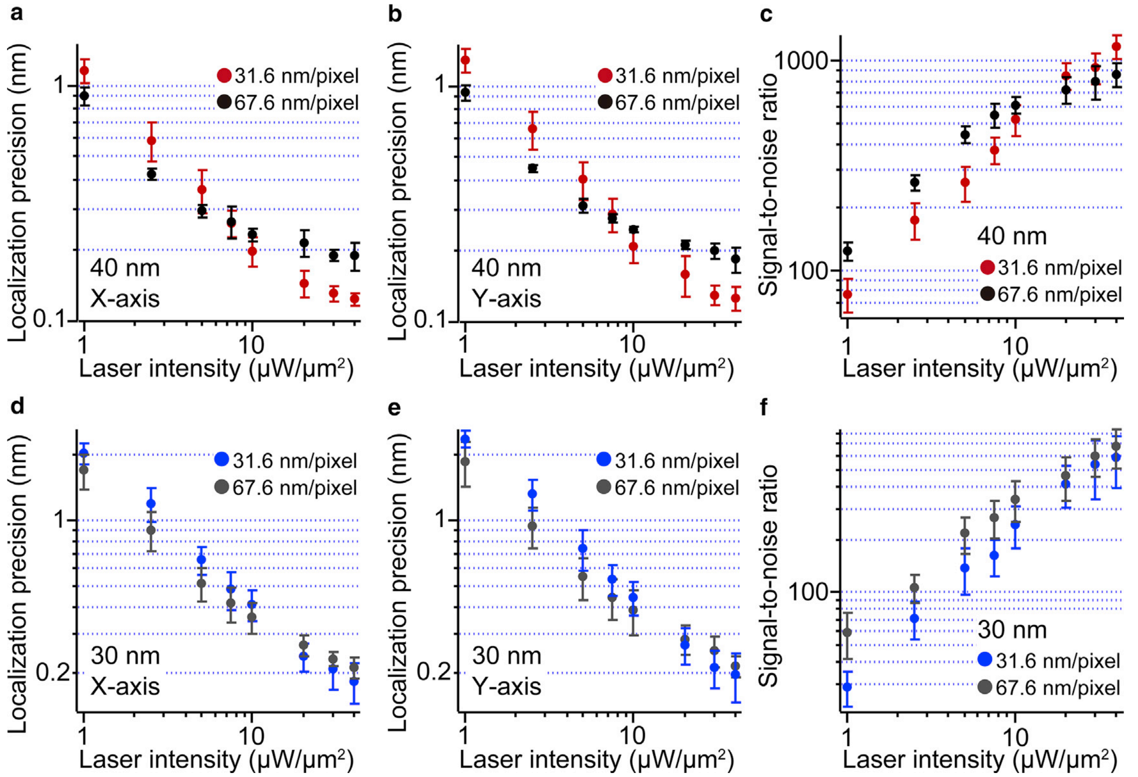


FIGURE 4 Localization precision of 40 and 30 nm AuNPs with annular illumination dark-field microscopy at high laser intensity and small image pixel size. (*a* and *b*) Log-log plots between laser intensity and localization precision for the 40 nm AuNPs along the (*a*) X and (*b*) Y axes ($n = 5$). The image pixel sizes were 31.6 nm/pixel (*red dots*) and 67.6 nm/pixel (*black dots*), and the image was taken at 1 ms time resolution. (*c*) The S/N of 40 nm AuNPs at different laser intensities ($n = 5$). The image pixel sizes were 31.6 nm/pixel (*red dots*) and 67.6 nm/pixel (*black dots*), respectively. (*d* and *e*) Log-log plots between laser intensity and localization precision for the 30 nm AuNPs along the (*d*) X and (*e*) Y axes ($n = 5$). The image pixel sizes were 31.6 nm/pixel (*blue dots*) and 67.6 nm/pixel (*gray dots*). The image was taken at 1 ms time resolution. (*f*) The S/N for the 30 nm AuNPs at different laser intensities ($n = 5$). The image pixel sizes were 31.6 nm/pixel (*blue dots*) and 67.6 nm/pixel (*gray dots*). All error bars represent SD. To see this figure in color, go online.

illumination shows strong polarization in the direction parallel to the substrate, and the direction parallel to the incident s-polarized light appears strongly (37). In an annular illumination system, total internal reflection occurs from all directions at the interface of glass and water. Polarization of the evanescent field is effectively reduced, which allows quantitative fluorescence or scattering measurements of polarization-sensitive molecules or metal nanostructures regardless of the sample orientation parallel to the glass substrate.

Elementary steps in the chemical reactions of biomolecular motors generally show time constants with millisecond timescales, and their mechanical motions (steps) triggered by chemical reaction generally occur on sub-millisecond timescales (6). Therefore, to understand the working principles of biomolecular motors in detail, time resolution must be improved to the microsecond level and must be accompanied by high localization precision. We next observed 40 and 30 nm AuNPs at high time resolution with high laser intensity. Fig. 6, *a–f* show the time-resolution dependence of the localization precision and S/N for 40 and 30 nm AuNPs at 40 $\mu$W/$\mu$m$^2$ laser intensity, respectively. For 40 nm AuNPs at time resolutions between 1 ms and 33 $\mu$s, localization precisions with 31.6 nm/pixel image pixel size were slightly better than those with 67.6 nm/pixel image pixel size, whereas at 10 $\mu$s time resolution, localization precision with 67.6 nm/pixel image pixel size was slightly better than that with 31.6 nm/pixel image pixel size. As results, localization precisions of 1.7 Å, 3.2 Å, 5.4 Å, and 1.3 nm were achieved at 333, 100, 33, and 10 $\mu$s time resolutions, respectively. On the other hand, for 30 nm AuNP, at time resolutions between 333 and 10 $\mu$s, localization precisions were slightly better with 67.6 nm/pixel image pixel size than those with 31.6 nm/pixel image pixel size, whereas at 1 ms time resolution, localization precision with 31.6 nm/pixel image pixel size was slightly better than that with 67.6 nm/pixel image pixel size. As results, localization precisions of 3.3 Å, 7.5 Å, 1.7 nm, and 4.4 nm were achieved at 333, 100, 33, and 10 $\mu$s time resolutions, respectively. Because the laser intensity was 40 $\mu$W/$\mu$m$^2$ for these measurements, detector saturation occurs with a large pixel size of 67.6 nm/pixel at a low time resolution such as 1 ms. This is the reason why worse localization precision was obtained with a large pixel size compared with the small image pixel size of 31.6 nm/pixel. On the other hand, at a high time resolution such as 10 $\mu$s,

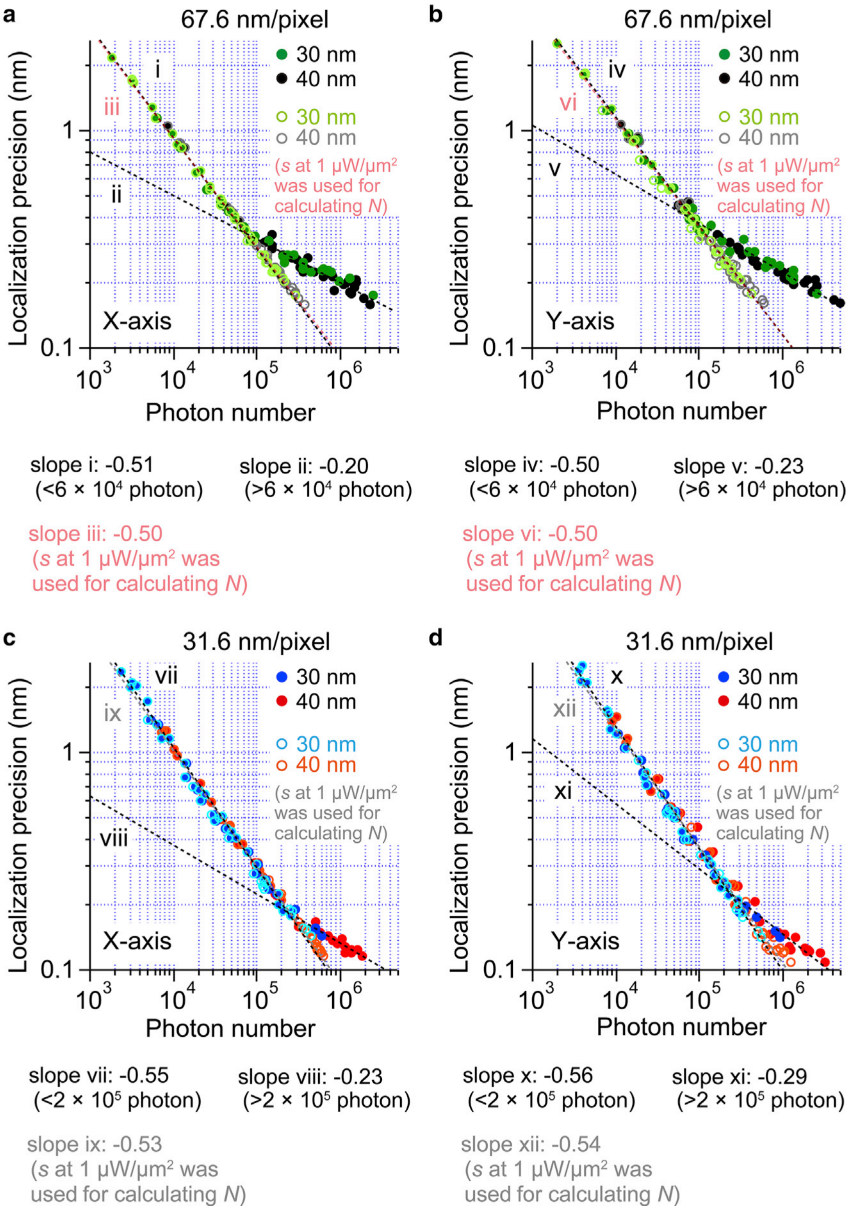


FIGURE 5 Photon number dependence of the localization precision for the 40 and 30 nm AuNPs at different image pixel sizes. (*a–d*) Log-log plots between estimated photon number and localization precision along the (*a*) X and (*b*) Y axes with the image pixel size of 67.6 nm/pixel and (*c*) X and (*d*) Y axes with the image pixel size of 31.6 nm/pixel. Black and red symbols represent 40 nm AuNPs, and green and blue symbols represent 30 nm AuNPs, respectively. Gray and orange (*light green* and *light blue*) symbols represent 40 nm (30 nm) AuNPs, for which the photon number was calculated with the *s* at 1 $\mu$W/$\mu$m$^2$ laser intensity. Black dotted lines show linear fits applied to the region below and above 60,000 photons at 67.6 nm/pixel image pixel size and to the region below and above 200,000 photons at 31.6 nm/pixel image pixel size. Light red and gray dotted lines show linear fits applied to the whole photon number region at 67.6 and 31.6 nm/pixel image pixel sizes, respectively, for which the photon number was calculated with the *s* at 1 $\mu$W/$\mu$m$^2$ laser intensity. The slopes of the dotted lines are −0.51 (*i*), −0.20 (*ii*), −0.50 (*iii*), −0.50 (*iv*), −0.23 (*v*), −0.50 (*vi*), −0.55 (*vii*), −0.23 (*viii*), −0.53 (*ix*), −0.56 (*x*), −0.29 (*xi*), and −0.54 (*xii*), respectively. To see this figure in color, go online.

detector saturation did not occur for both image pixel sizes, corresponding to the same numbers of collected photons. Under this condition, localization precision will be determined by the balance of the image pixel size *a* and the SD of the background *b* in Eq. 4. With a fixed value of 10,000 photons (*N*) and image width (*s*) of 100 nm and the experimentally determined values of *a* and *b*, the localization precisions were estimated to be 1.1 nm for 67.6 nm/pixel and 2.2 nm for 31.6 nm/pixel, respectively. This estimation is consistent with better localization precision with 67.6 nm/-pixel at 10 $\mu$s time resolution.

To verify the capability of the developed system for single-molecule imaging of biomolecular motors, we observed linear motion of kinesin-1 labeled with 40 nm AuNP at 10 $\mu$s time resolution (Fig. 7; Video S1). We analyzed the transition from the microtubule-bound to the microtubule-unbound state of the kinesin-1 head in detail. This transition is a transient event that occurs on a microsecond timescale. Because the time resolution of our current system (10 $\mu$s) is 5.5 times higher than that of a previous study with similar nanometer localization precision (24), it should provide better insights on the pathway of transition. As reported previously (24), we used heterodimeric cysteine-light human kinesin-1, whose one head has cysteine residue (S55C). The cysteine residue was biotinylated and bound with a streptavidin-coated 40 nm AuNP, and transitions of the head were observed by annular illumination dark-field imaging system at 10 $\mu$M ATP. Fig. 7 *a* shows the typical trace of the center position of a 40 nm AuNP attached to the kinesin-1 head along the microtubule long axis (on axis)

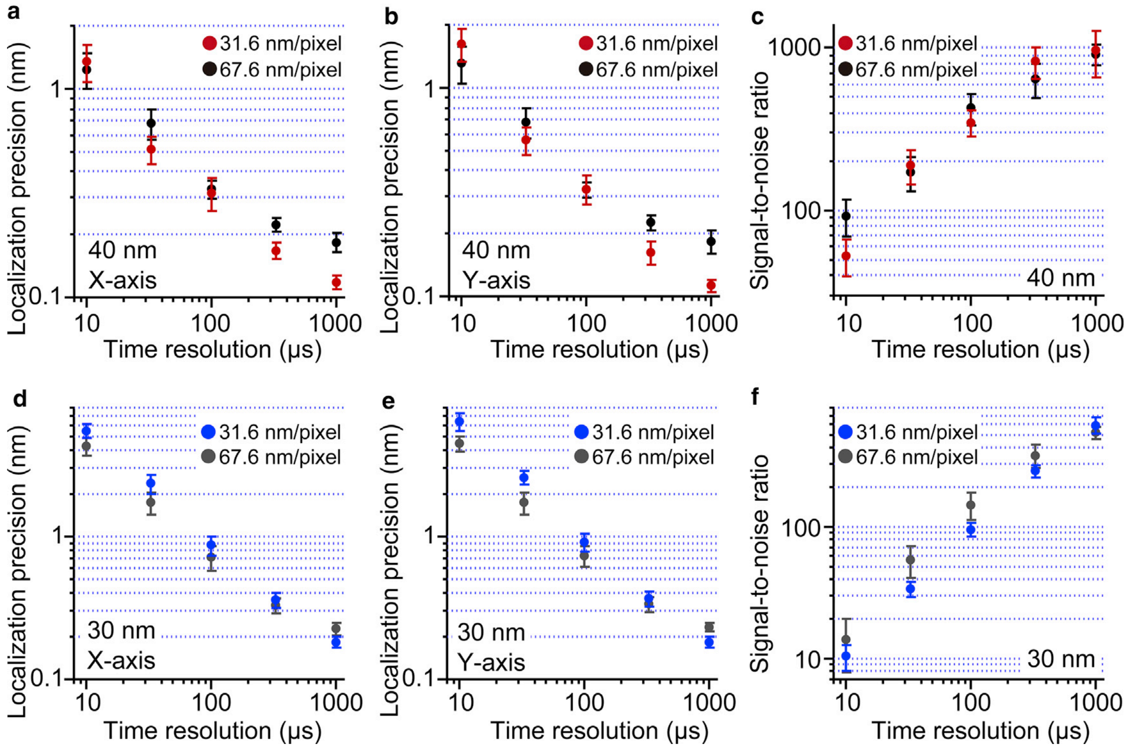


FIGURE 6 Time-resolution dependence of the localization precision of 40 and 30 nm AuNPs with annular illumination dark-field microscopy. (*a* and *b*) Time-resolution dependence of localization precision along the (*a*) X and (*b*) Y axes for 40 nm AuNPs ($n = 5$). The dark-field image was obtained with 40 $\mu$W/$\mu$m$^2$ laser intensity. The image pixel sizes were 31.6 nm/pixel (*red dots*) and 67.6 nm/pixel (*black dots*). (*c*) Time-resolution dependence of the S/N for 40 nm AuNPs ($n = 5$). The dark-field image was obtained with 40 $\mu$W/$\mu$m$^2$ laser intensity. The image pixel sizes were 31.6 nm/pixel (*red dots*) and 67.6 nm/pixel (*black dots*). (*d* and *e*) Time-resolution dependence of the localization precision along the (*d*) X and (*e*) Y axes for 30 nm AuNPs ($n = 5$). The dark-field image was obtained with 40 $\mu$W/$\mu$m$^2$ laser intensity. The image pixel sizes were 31.6 nm/pixel (*blue dots*) and 67.6 nm/pixel (*gray dots*), respectively. (*f*) Time-resolution dependence of the S/N for 30 nm AuNPs ($n = 5$). The dark-field image was obtained with 40 $\mu$W/$\mu$m$^2$ laser intensity. The image pixel sizes were 31.6 nm/pixel (*blue dots*), and 67.6 nm/pixel (*gray dots*). All error bars represent SD. To see this figure in color, go online.

and perpendicular to the microtubule long axis (off axis). Processive movement of kinesin-1 along the microtubule long axis was observed. The lower panel shows the SD of the on- and off-axis directions. In both of the axes, low fluctuation state (colored as *red*) and high fluctuation state (colored as *blue*) were observed, representing the microtubule-bound state and microtubule-unbound state of the head, respectively. When the bound state is changed to the unbound state, the head displaced rightward in the off axis, as reported previously (24). Figs. 7, *b* and *c* and S14 show enlarged views of the representative traces and SDs, including transitions from the bound to the unbound state. The traces during transition are shown by a rainbow-colored line. The periods with small (*light red*) and large (*light blue*) SD correspond to the bound and unbound states, respectively, whereas the period when the SD changed from small to large value (*rainbow color*) corresponds to the transition. Figs. 7 *d* and S14 shows two-dimensional plots of the center position of 40 nm AuNPs. During transitions, there were no apparent large leftward trails in the off axis because they did not move out to the left from the area of bound states shown as red. This result strongly suggests that the trailing head passes the microtubule-bound leading head from the right during state transition, which results in unidirectional rotation of kinesin-1 (38).

Although the inertial and viscosity forces of AuNPs in water are sufficiently small and low-load fast motion of biomolecular motors can be observed, reduction of the particle size is still important to minimize any possible hindrances due to the steric effect of AuNPs. Based on the Mie theory, the scattering cross section of the particle ($\sigma_{\text{scatt}}$) is given by

$$\sigma_{\text{scatt}} = \frac{128\pi^5}{3\lambda^4} R^6 \left| \frac{m^2 - 1}{m^2 + 2} \right|^2, \tag{5}$$

where $\lambda$, $R$, and $m$ are the wavelength of the light, the radius of the particle, and the ratio of the refractive index between the particle and the surrounding medium, respectively (39). Furthermore, the scattering efficiency ($E_{\text{scatt}}$) is given by the scattering cross section divided by the geometric cross section as $\pi R^2$ (40) and is expressed by the following:

$$E_{\text{scatt}} = \frac{\sigma_{\text{scatt}}}{\pi R^2} = \frac{128\pi^4}{3\lambda^4} R^4 \left| \frac{m^2 - 1}{m^2 + 2} \right|^2. \tag{6}$$

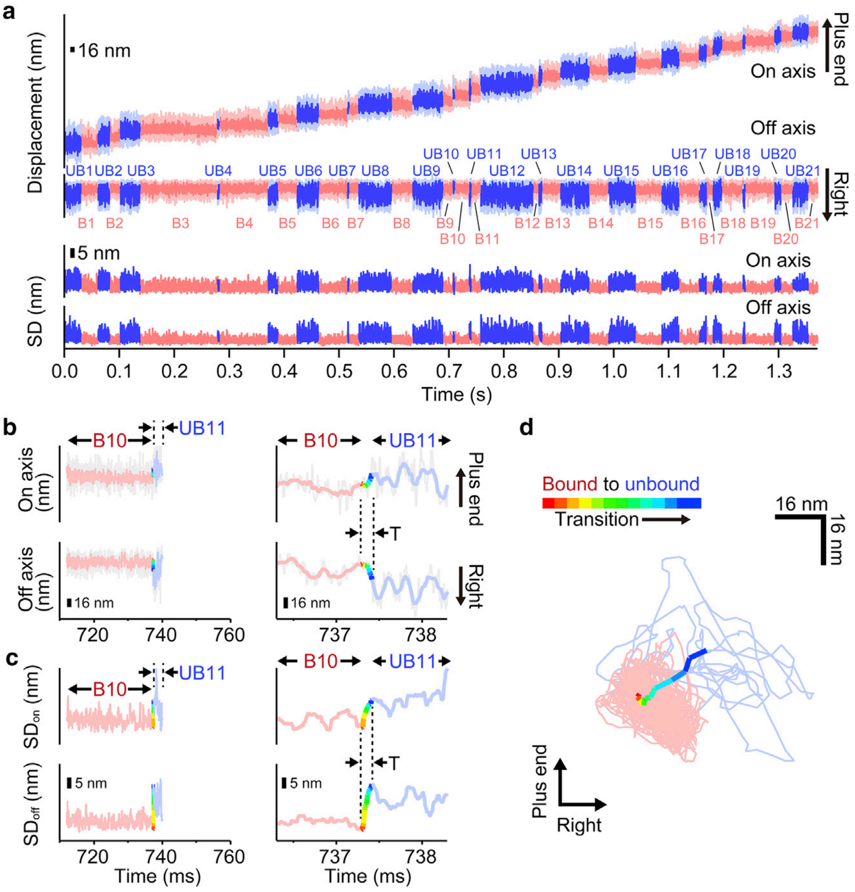


FIGURE 7 Transition from bound to unbound state of the kinesin-1 head labeled with 40 nm AuNP and observed at 10 $\mu$s time resolution. (*a*) Typical trace of the center position at 10 $\mu$M ATP. Light red and light blue lines represent the bound and the unbound states, respectively. Red and blue lines represent the filtered trace with moving average (window size of 10 frames) of the bound and the unbound states, respectively. Lower panel shows SD of the positions along the on and off axis for each time frame (calculated as (t − 10, t + 10)). (*b*) An enlarged view of the trace in (*a*), including the transition from the bound to the unbound state (*gray line*). Red, blue, and rainbow-colored lines represent the filtered trace with moving average (window size of 10 frames) for the bound state, the unbound state, and the transition from the bound to the unbound state, respectively. (*c*) An enlarged view of the SD of the positions along the on and off axis. Red, blue, and rainbow-colored lines represent the bound state, the unbound state, and the transition from the bound to the unbound state, respectively. (*d*) A two-dimensional plot of the center position of a 40 nm AuNP, shown in (*b*). Light red, light blue, and rainbow lines represent filtered trace with moving average (window size of 10 frames) for the bound state, the unbound state, and the transition from the bound to the unbound state, respectively. To see this figure in color, go online.

Next, we investigated the relationship between particle size and the signal intensity or S/N (Fig. S15). The slope of the linear fit to the log-log plot for the signal intensity was 4.4 at 4 $\mu$W/$\mu$m$^2$ laser intensity. Similarly, the slope of the linear fit to the log-log plot for the S/N was around 4 when the laser intensity was lower than 5 $\mu$W/$\mu$m$^2$. When the laser intensity was higher than 5 $\mu$W/$\mu$m$^2$, the slope became less steep, presumably because of S/N saturation. Both the signal intensity and the S/N were proportional to ~4th power of the particle diameter. Furthermore, we analyzed the dependence of the signal intensity or S/N on the particle diameter with vertical illumination dark-field imaging system (Fig. S16) (28). Slopes of the linear fit to the log-log plot for the signal intensity and the S/N were 4.2 and 3.9, respectively. Therefore, the fourth-power diameter dependency is independent of the illumination conditions. These results suggest that when scattering light is collected with microscope system, we may need to consider the geometrical cross section of AuNP. The infinitesimal point is projected on the image plane in the microscope system. However, in actual measurement, the nanoparticle has finite size. The scattering cross section divided by the geometrical cross section, which is equivalent to the scattering efficiency (Eq. 6, scales with $R^4$), is consistent with our observation, the fourth-power diameter dependency. This severe scaling law causes difficulty in observing small AuNPs with high localization precision. Thus, it is important to use much higher laser intensity than that used for 40 nm AuNPs. Our annular illumination system is suitable for this purpose because we achieved 1.9 Å, 7.5 Å, and 1.7 nm localization precisions at 1 ms, 100 $\mu$s, and 33 $\mu$s time resolutions, respectively, with 30 nm AuNP (Fig. 6).

To verify whether our system with smaller AuNPs works for the analysis of biomolecular motors, we used a 30 nm AuNP to observe the motion of the kinesin-1 head at 50 $\mu$s time resolution. We observed the same kinesin-1 construct described above at 10 $\mu$M ATP (Fig. 8; Video S2). Fig. 8 *a* shows the typical trace of the center position of a 30 nm AuNP attached to the kinesin-1 head. The processive movement of kinesin-1 along the microtubule long axis was observed with the distinct steps. The distribution of the step size shows a single peak at around 16.1 nm (seven molecules, 212 steps) (Fig. S17). The average velocity was 218.5 ± 41.6 nm/s (seven molecules, 12 traces). Both the step size and velocity were similar to the previous report using a 40 nm AuNP (24). The lower panel in Fig. 8 *a* shows the SD of the on- and off-axis directions. In both of the axes, low fluctuation state (colored as *red*) and high fluctuation state (colored as *blue*) were observed. Fig. 8 *b* shows

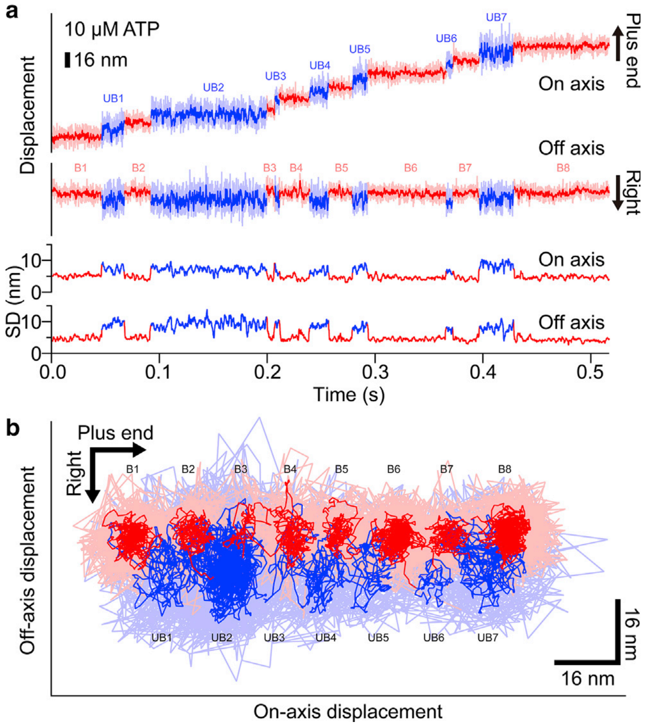


FIGURE 8 Observation of kinesin-1 head labeled with 30 nm AuNP at 50 $\mu$s time resolution and at 10 $\mu$M ATP. (*a*) A typical trace of the center position of a 30 nm AuNP attached to a kinesin-1 head. Light red and light blue lines represent the bound state and the unbound states, respectively. Red and blue lines represent a filtered trace with moving average (window size of 10 frames) of the bound and the unbound states, respectively. The lower panel shows SD of the positions along the on and off axis for each time t (calculated as (t − 20, t + 20)). (*b*) A two-dimensional plot of the center position of a 30 nm AuNP, shown in (*a*). Light red and light blue lines show the raw trace, and red and blue lines represent the filtered trace with moving average (window size of 10 frames) of the bound and the unbound states, respectively. Numbers correspond to the location of the bound and the unbound state, shown in (*a*). To see this figure in color, go online.

a two-dimensional plot of the center position of the 30 nm AuNP. When the bound state is changed to the unbound state, the kinesin-1 head displaced rightward in the off axis, as seen with the 40 nm AuNP (24). We confirmed that the bound state and the unbound state of the head were distinguished by using a 30 nm AuNP as a probe at 50 $\mu$s time resolution.

Recently, interferometric scattering microscopy (iSCAT) has been developed as a highly sensitive detection technique that visualizes small AuNPs and even unlabeled proteins (41–44). Dependence of the iSCAT signal on the radius of AuNP is $R^3$ because of interference between scattered light from the sample and the reference beam, such as light reflected from the glass surface. The localization precisions of 2.0 nm for 20 nm AuNPs at 2 $\mu$s time resolution (45) and 0.9 nm for 20 nm AuNPs at 10 ms time resolution (46) were obtained with 10–20 kW/cm$^2$ laser intensity (100–200 $\mu$W/$\mu$m$^2$). In the case of iSCAT, the detector can reach saturation earlier because of the high intensity reference beam, which can limit the localization precision, although detector saturation has been improved recently by using a partial reflector (47,48). Dark-field microscopy, on the other hand, selectively observes scattered light from the AuNP by eliminating illumination light. The photon number from AuNPs can be increased up to detector saturation, resulting in the angstrom-level precision we achieved.

In summary, we achieved angstrom localization precision and microsecond time resolution in dark-field imaging of AuNPs. We quantitatively analyzed the localization precision by changing various experimental parameters, and we confirmed that the photon number determined the localization precision, which is inversely proportional to the square root of the photon number. We also concluded that the major factor limiting the localization precision is detector saturation. To achieve localization precision at the angstrom level, we developed an axicon-based annular illumination dark-field microscope that can illuminate the sample with high laser intensity without damaging the objective lens. We achieved 1.3 Å localization precision for 40 nm AuNPs at 1 ms time resolution at high laser intensity and small image pixel size, which effectively suppresses detector saturation. We also investigated the localization precision with high time resolution. A localization precision of 5.4 Å was achieved at 33 $\mu$s time resolution with our optical system for 40 nm AuNPs. Furthermore, we examined the size dependence of the AuNPs on the localization precision. The signal intensity from AuNPs decreased with the fourth power of particle diameter, resulting in significantly decreased signal intensity from small AuNPs. Despite this drawback, we observed 30 nm AuNPs with 1.9 Å and 1.7 nm localization precisions at 1 ms and 33 $\mu$s time resolutions, respectively. We successfully applied our annular illumination system to the observation of the motion of kinesin-1. The bound and unbound states of the head of kinesin-1 on the microtubule during stepping motion were observed with a 30 nm AuNP at 50 $\mu$s time resolution, and the transition pathways from the bound to the unbound state were captured with a 40 nm AuNP at 10 $\mu$s time resolution. Our method enables the analysis of biomolecular motor dynamics with near-atomic localization precision and microsecond time resolution and will contribute to further understanding of the working principles of biomolecular motors.

## SUPPORTING MATERIAL

Seventeen figures and two videos are available at http://www.biophysj.org/biophysj/supplemental/S0006-3495(18)31280-3.

## AUTHOR CONTRIBUTIONS

R.I. conceived and supervised the project. J.A. and A.N. performed dark-field imaging of AuNPs with point-illumination total internal reflection dark-field microscopy and analyzed the localization precision data. J.A. constructed the axicon-based annular illumination dark-field microscopy

system, performed dark-field imaging of AuNPs, and analyzed the localization precision data. J.A. performed dark-field imaging of AuNPs with vertical illumination dark-field microscopy system and analyzed the localization precision data. A.N. performed fluorescence imaging of Cy3-labeled chitinase A with total internal reflection fluorescence microscopy. C.S. and K.M. performed electron microscope examinations of AuNPs. J.A. analyzed the electron microscope image of AuNPs. J.A., A.N., A.V., and M.Y. performed the purification of tubulin from pig brain. J.A. and M.Y. performed the purification and labeling of kinesin-1. J.A. and M.Y. performed surface coating of the AuNP. J.A. performed the dark-field imaging of kinesin-1 and analyzed the data. J.A., A.N., and R.I. wrote the manuscript.

## ACKNOWLEDGMENTS

We thank Dr. Shouichi Sakakihara for his support with fabricating optical components used in the dark-field imaging system. We thank Prof. Ryuji Yokokawa and Dr. Kazuya Fujimoto for advice on the preparation of tubulin samples. We thank Prof. Michio Tomishige for his providing plasmid for kinesin-1 heterodimer construct.

This study was supported by Grant-in-Aid for Scientific Research on Innovative Areas "Molecular Engine" (grant number JP18H05424 to R.I.), Grants-in-Aid for Scientific Research (grant numbers JP18H02418, JP18H04755, JP17K19213 to R.I. and JP18H01904 to J.A.) from the Ministry of Education, Culture, Sports, Science, and Technology of Japan, and the Imaging Science Project of the Center for Novel Science Initiatives, National Institutes of Natural Sciences, Japan (IS291003 to J.A.).

## REFERENCES

1. Funatsu, T., Y. Harada, …, T. Yanagida. 1995. Imaging of single fluorescent molecules and individual ATP turnovers by single myosin molecules in aqueous solution. *Nature.* 374:555–559.
2. Sase, I., H. Miyata, …, K. Kinosita, Jr. 1995. Real time imaging of single fluorophores on moving actin with an epifluorescence microscope. *Biophys. J.* 69:323–328.
3. Sako, Y., S. Minoghchi, and T. Yanagida. 2000. Single-molecule imaging of EGFR signalling on the surface of living cells. *Nat. Cell Biol.* 2:168–172.
4. Iino, R., I. Koyama, and A. Kusumi. 2001. Single molecule imaging of green fluorescent proteins in living cells: E-cadherin forms oligomers on the free cell surface. *Biophys. J.* 80:2667–2677.
5. Shen, H., L. J. Tauzin, …, C. F. Landes. 2017. Single particle tracking: from theory to biophysical applications. *Chem. Rev.* 117:7331–7376.
6. Iino, R., T. Iida, …, Y. Sako. 2018. Single-molecule imaging and manipulation of biomolecular machines and systems. *Biochim Biophys Acta Gen Subj.* 1862:241–252.
7. Gelles, J., B. J. Schnapp, and M. P. Sheetz. 1988. Tracking kinesin-driven movements with nanometre-scale precision. *Nature.* 331:450–453.
8. Dahan, M., S. Lévi, …, A. Triller. 2003. Diffusion dynamics of glycine receptors revealed by single-quantum dot tracking. *Science.* 302:442–445.
9. DeWitt, M. A., A. Y. Chang, …, A. Yildiz. 2012. Cytoplasmic dynein moves through uncoordinated stepping of the AAA+ ring domains. *Science.* 335:221–225.
10. Geerts, H., M. De Brabander, …, P. Hollenbeck. 1987. Nanovid tracking: a new automatic method for the study of mobility in living cells based on colloidal gold and video microscopy. *Biophys. J.* 52:775–782.
11. Yasuda, R., H. Noji, …, H. Itoh. 2001. Resolution of distinct rotational substeps by submillisecond kinetic analysis of F1-ATPase. *Nature.* 410:898–904.
12. Ober, R. J., S. Ram, and E. S. Ward. 2004. Localization accuracy in single-molecule microscopy. *Biophys. J.* 86:1185–1200.
13. Thompson, R. E., D. R. Larson, and W. W. Webb. 2002. Precise nanometer localization analysis for individual fluorescent probes. *Biophys. J.* 82:2775–2783.
14. Yildiz, A., J. N. Forkey, …, P. R. Selvin. 2003. Myosin V walks hand-over-hand: single fluorophore imaging with 1.5-nm localization. *Science.* 300:2061–2065.
15. Yildiz, A., M. Tomishige, …, P. R. Selvin. 2004. Kinesin walks hand-over-hand. *Science.* 303:676–678.
16. Kural, C., H. Kim, …, P. R. Selvin. 2005. Kinesin and dynein move a peroxisome in vivo: a tug-of-war or coordinated movement? *Science.* 308:1469–1472.
17. Levi, V., V. I. Gelfand, …, E. Gratton. 2006. Melanosomes transported by myosin-V in Xenopus melanophores perform slow 35 nm steps. *Biophys. J.* 90:L07–L09.
18. Nan, X., P. A. Sims, …, X. S. Xie. 2005. Observation of individual microtubule motor steps in living cells with endocytosed quantum dots. *J. Phys. Chem. B.* 109:24220–24224.
19. Abbondanzieri, E. A., W. J. Greenleaf, …, S. M. Block. 2005. Direct observation of base-pair stepping by RNA polymerase. *Nature.* 438:460–465.
20. Wen, J. D., L. Lancaster, …, I. Tinoco. 2008. Following translation by single ribosomes one codon at a time. *Nature.* 452:598–603.
21. Greenleaf, W. J., M. T. Woodside, and S. M. Block. 2007. High-resolution, single-molecule measurements of biomolecular motion. *Annu. Rev. Biophys. Biomol. Struct.* 36:171–190.
22. Moffitt, J. R., Y. R. Chemla, …, C. Bustamante. 2008. Recent advances in optical tweezers. *Annu. Rev. Biochem.* 77:205–228.
23. Minagawa, Y., H. Ueno, …, R. Iino. 2013. Basic properties of rotary dynamics of the molecular motor Enterococcus hirae V1-ATPase. *J. Biol. Chem.* 288:32700–32707.
24. Isojima, H., R. Iino, …, M. Tomishige. 2016. Direct observation of intermediate states during the stepping motion of kinesin-1. *Nat. Chem. Biol.* 12:290–297.
25. Ueno, H., Y. Minagawa, …, R. Iino. 2014. Torque generation of Enterococcus hirae V-ATPase. *J. Biol. Chem.* 289:31212–31223.
26. Suzuki, T., K. Tanaka, …, M. Yoshida. 2014. Chemomechanical coupling of human mitochondrial F1-ATPase motor. *Nat. Chem. Biol.* 10:930–936.
27. Nan, X., P. A. Sims, and X. S. Xie. 2008. Organelle tracking in a living cell with microsecond time resolution and nanometer spatial precision. *Chemphyschem.* 9:707–712.
28. Ueno, H., S. Nishikawa, …, H. Noji. 2010. Simple dark-field microscopy with nanometer spatial precision and microsecond temporal resolution. *Biophys. J.* 98:2014–2023.
29. Cheezum, M. K., W. F. Walker, and W. H. Guilford. 2001. Quantitative comparison of algorithms for tracking single fluorescent particles. *Biophys. J.* 81:2378–2388.
30. Nishizaka, T., Y. Hasimoto, and T. Masaike. 2011. Simultaneous observation of chemomechanical coupling of a molecular motor. *Methods Mol. Biol.* 778:259–271.
31. Nakamura, A., T. Tasaki, …, R. Iino. 2018. Rate constants, processivity, and productive binding ratio of chitinase A revealed by single-molecule analysis. *Phys. Chem. Chem. Phys.* 20:3010–3018.
32. Kerssemakers, J. W., E. L. Munteanu, …, M. Dogterom. 2006. Assembly dynamics of microtubules at molecular resolution. *Nature.* 442:709–712.
33. Kubitscheck, U., O. Kückmann, …, R. Peters. 2000. Imaging and tracking of single GFP molecules in solution. *Biophys. J.* 78:2170–2179.
34. Koyama-Honda, I., K. Ritchie, …, A. Kusumi. 2005. Fluorescence imaging for monitoring the colocalization of two single molecules in living cells. *Biophys. J.* 88:2126–2136.

35. Nakamura, A., K. I. Okazaki, …, R. Iino. 2018. Processive chitinase is Brownian monorail operated by fast catalysis after peeling rail from crystalline chitin. *Nat. Commun.* 9:3814.

36. Makyio, H., R. Iino, …, S. Iwata. 2005. Structure of a central stalk subunit F of prokaryotic V-type ATPase/synthase from Thermus thermophilus. *EMBO J.* 24:3974–3983.

37. Axelrod, D., T. P. Burghardt, and N. L. Thompson. 1984. Total internal reflection fluorescence. *Annu. Rev. Biophys. Bioeng.* 13:247–268.

38. Ramaiya, A., B. Roy, …, E. Schäffer. 2017. Kinesin rotates unidirectionally and generates torque while walking on microtubules. *Proc. Natl. Acad. Sci. USA.* 114:10894–10899.

39. van Dijk, M. A., A. L. Tchebotareva, …, B. Lounis. 2006. Absorption and scattering microscopy of single metal nanoparticles. *Phys. Chem. Chem. Phys.* 8:3486–3495.

40. van de Hulst, H. C. 1957. Light Scattering by Small Particles. John Wiley & Sons, Inc., New York.

41. Ortega Arroyo, J., D. Cole, and P. Kukura. 2016. Interferometric scattering microscopy and its combination with single-molecule fluorescence imaging. *Nat. Protoc.* 11:617–633.

42. Jacobsen, V., P. Stoller, …, V. Sandoghdar. 2006. Interferometric optical detection and tracking of very small gold nanoparticles at a water-glass interface. *Opt. Express.* 14:405–414.

43. Wu, H. M., Y. H. Lin, …, C. L. Hsieh. 2016. Nanoscopic substructures of raft-mimetic liquid-ordered membrane domains revealed by high-speed single-particle tracking. *Sci. Rep.* 6:20542.

44. Andrecka, J., J. Ortega Arroyo, …, P. Kukura. 2016. Label-free imaging of microtubules with sub-nm precision using interferometric scattering microscopy. *Biophys. J.* 110:214–217.

45. Lin, Y. H., W. L. Chang, and C. L. Hsieh. 2014. Shot-noise limited localization of single 20 nm gold particles with nanometer spatial precision within microseconds. *Opt. Express.* 22:9159–9170.

46. Andrecka, J., J. Ortega Arroyo, …, P. Kukura. 2015. Structural dynamics of myosin 5 during processive motion revealed by interferometric scattering microscopy. *eLife.* 4:e05413.

47. Cole, D., G. Young, …, P. Kukura. 2017. Label-free single-molecule imaging with numerical-aperture-shaped interferometric scattering microscopy. *ACS Photonics.* 4:211–216.

48. Liebel, M., J. T. Hugall, and N. F. van Hulst. 2017. Ultrasensitive label-free nanosensing and high-speed tracking of single proteins. *Nano Lett.* 17:1277–1281.

Biophysical Journal, Volume *115*

## Supplemental Information

# Single-Nanoparticle Tracking with Angstrom Localization Precision and Microsecond Time Resolution

**Jun Ando, Akihiko Nakamura, Akasit Visootsat, Mayuko Yamamoto, Chihong Song, Kazuyoshi Murata, and Ryota Iino**

**Supplemental Information**

# Single-nanoparticle tracking with angstrom localization precision and microsecond time resolution

Jun Ando,[1,2] Akihiko Nakamura,[1,2] Akasit Visootsat,[1,2] Mayuko Yamamoto,[1] Chihong Song,[3] Kazuyoshi Murata,[3] and Ryota Iino[1,2]

[1]Institute for Molecular Science, National Institutes of Natural Sciences, Okazaki, Aichi, Japan; [2]The Graduate University for Advanced Studies (SOKENDAI), Hayama, Kanagawa, Japan; [3]National Institute for Physiological Sciences, National Institutes of Natural Sciences, Okazaki, Aichi, Japan

**Contents**

**Fig. S1-S17**

**Video S1, S2**

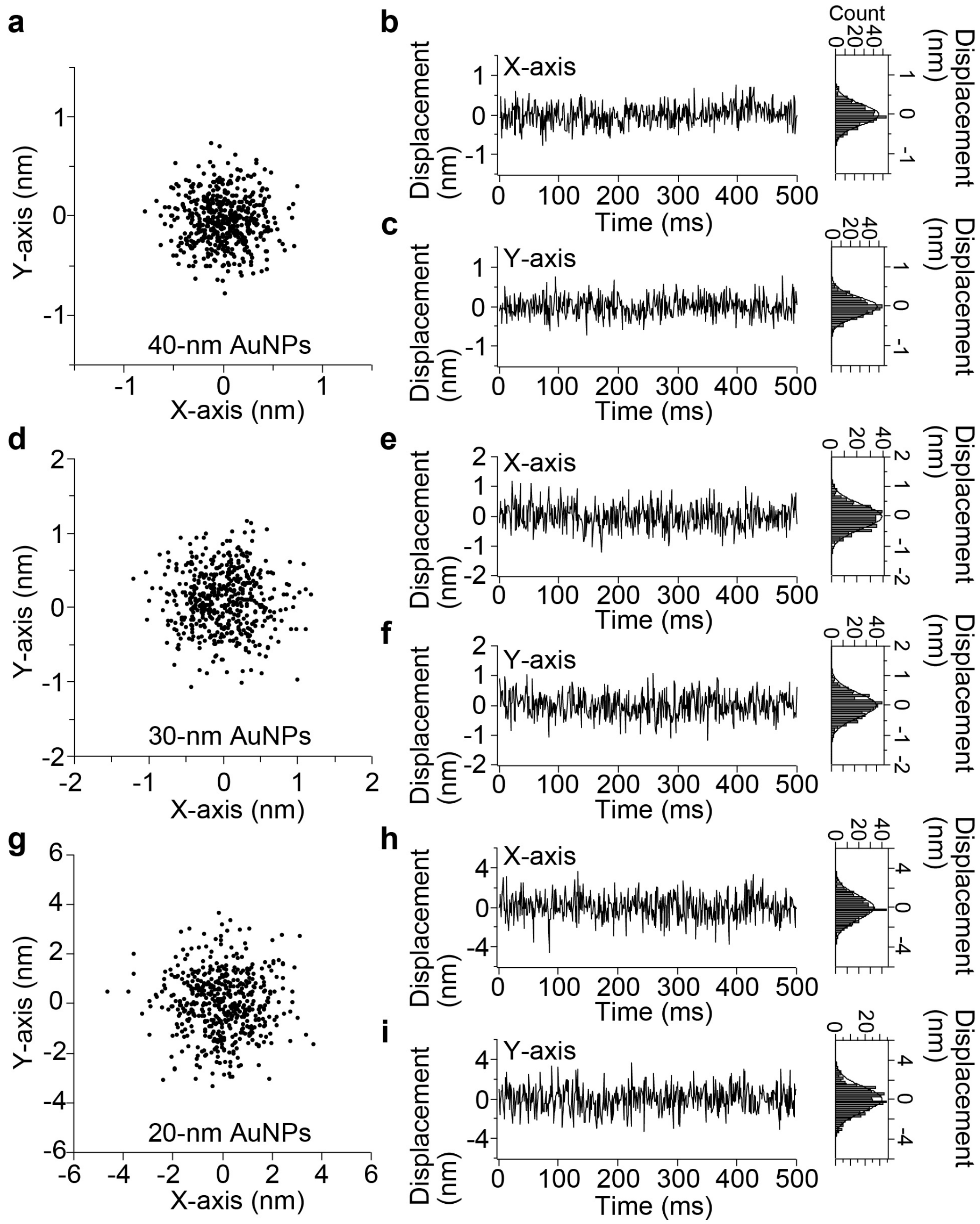


**Fig. S1.** Examples of two-dimensional plots, time courses, and histograms with Gaussian fits at the center for the dark-field images of (*a-c*) 40 nm, (*d-f*) 30 nm and (*g-i*) 20 nm AuNPs. The image was taken at 1 ms time resolution for 0.5 s with 10 μW/μm$^2$ laser intensity.

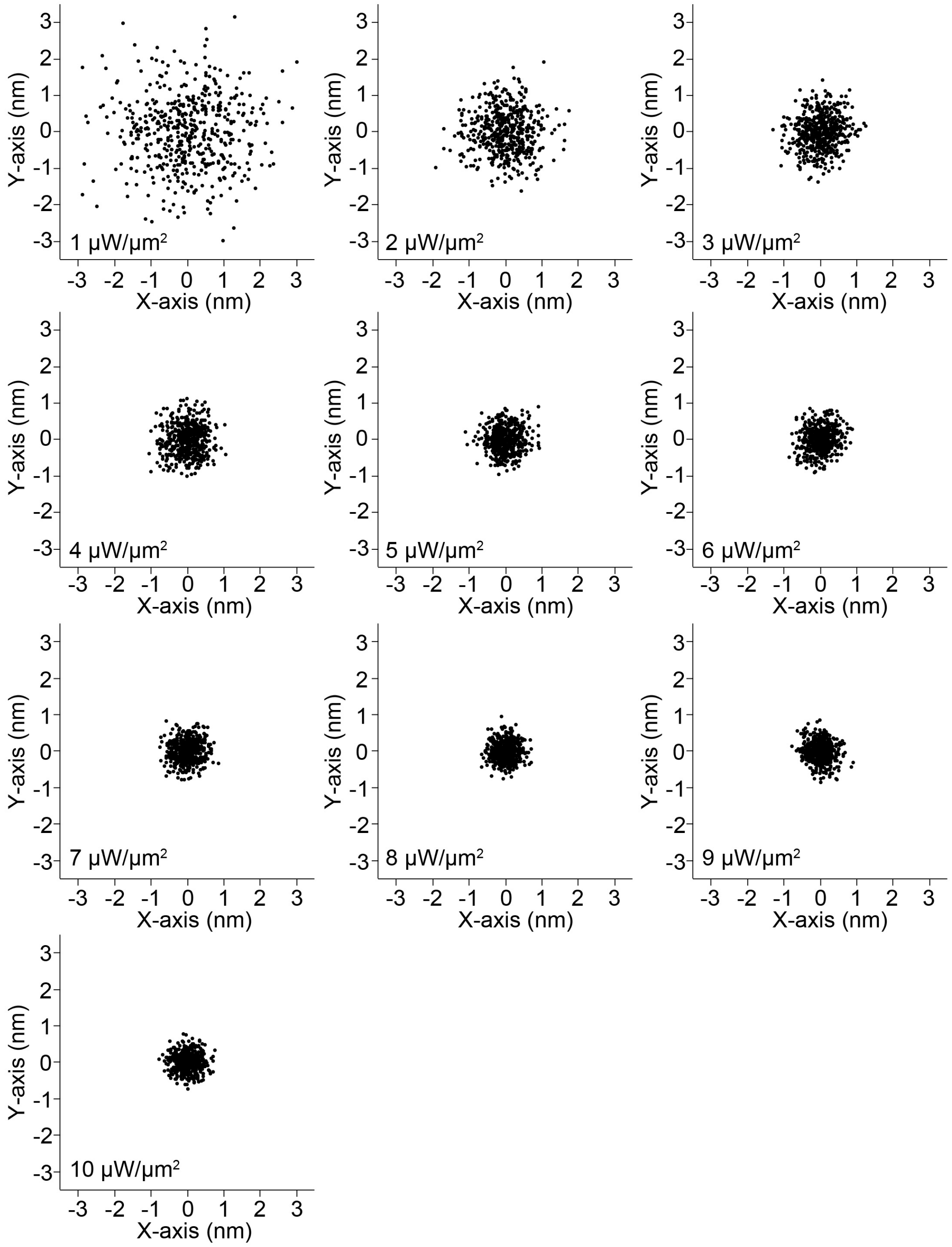


**Fig. S2.** Examples of two-dimensional plots in the center of the dark-field image of 40 nm AuNPs with laser intensities ranging from 1 to 10 μW/μm$^2$.

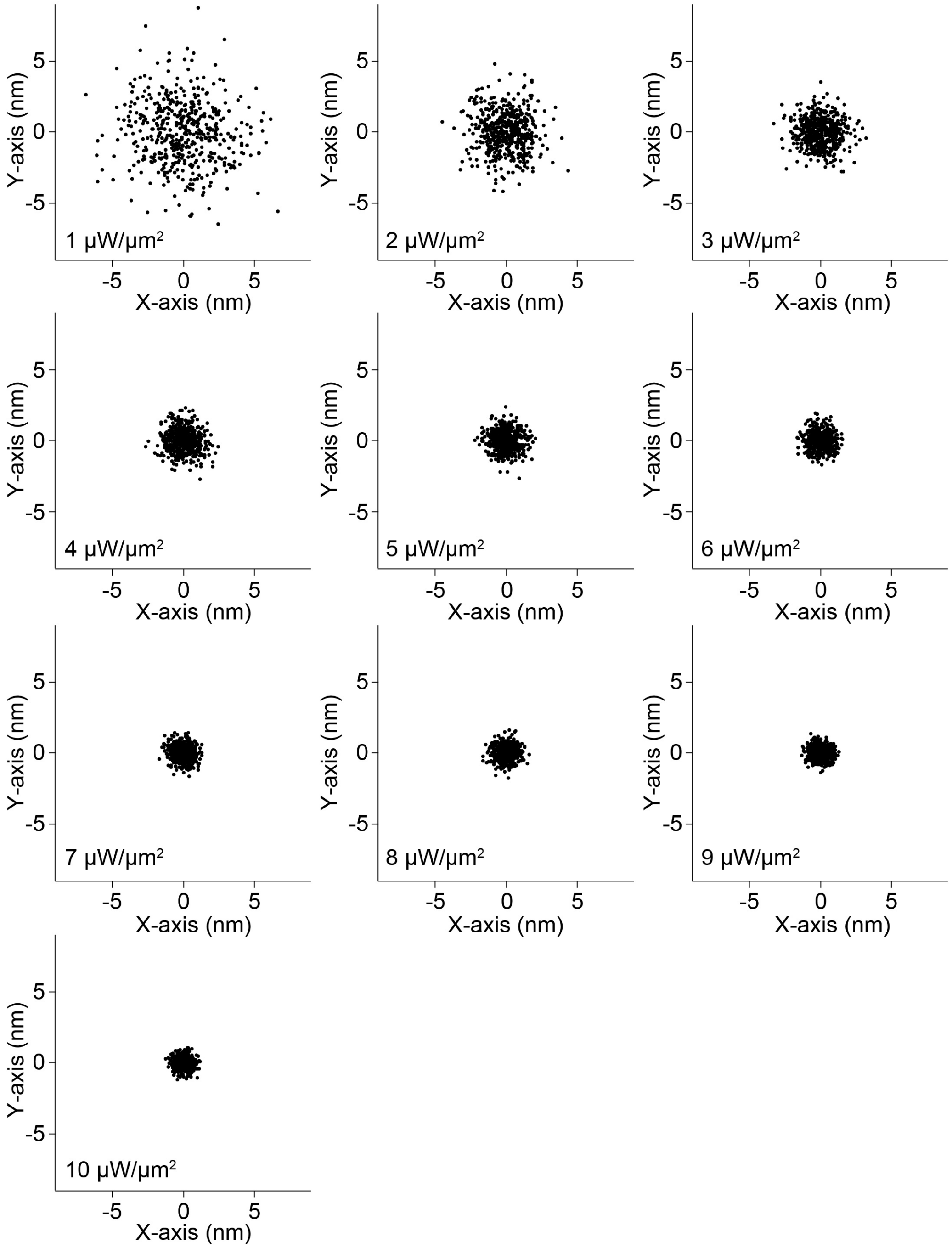


**Fig. S3.** Examples of two-dimensional plots in the center of the dark-field image of 30 nm AuNPs with laser intensities ranging from 1 to 10 μW/μm$^2$.

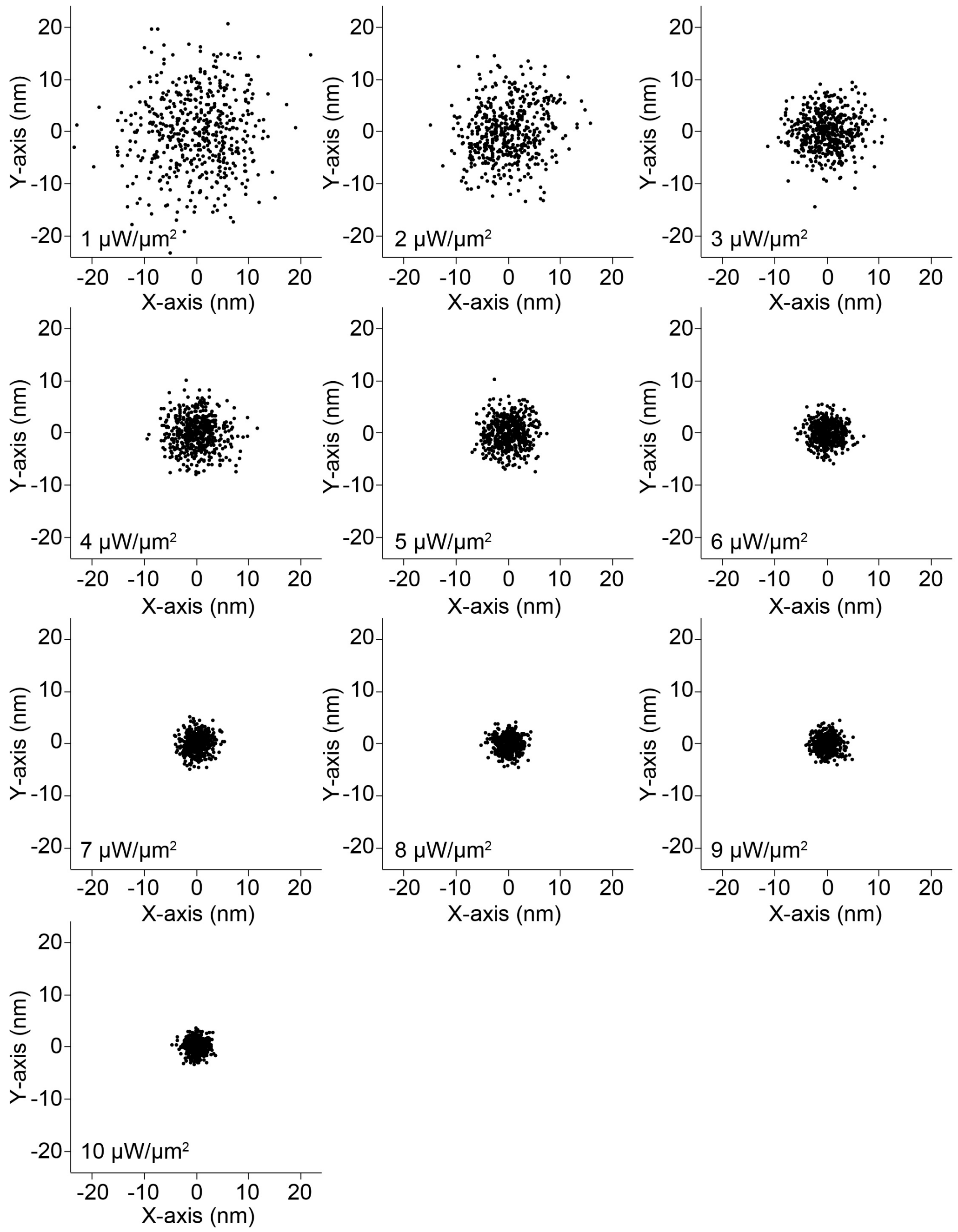


**Fig. S4.** Examples of two-dimensional plots in the center of the dark-field image of 20 nm AuNPs with laser intensities ranging from 1 to 10 μW/μm$^2$.

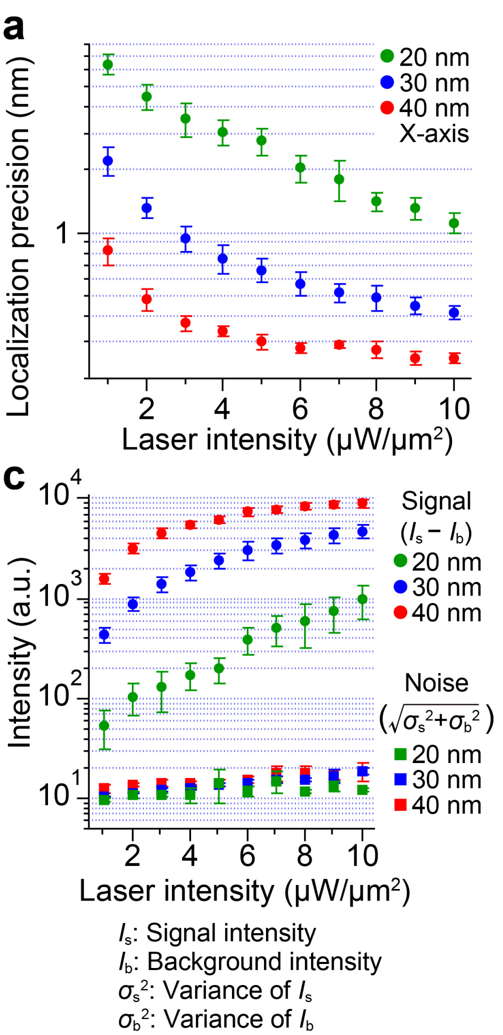


**Fig. S5.** Laser intensity dependence of the localization precision. (*a,b*) Localization precision of the dark-field image of 20 nm, 30 nm, and 40 nm AuNPs with different laser intensities along the (a) X- and (b) Y-axis ($n = 5$). The image was captured at 1 ms time resolution. Green, blue, and red circles represent 20 nm, 30 nm, and 40 nm AuNPs, respectively. (*c*) Signal and noise of the dark-field images of 20 nm, 30 nm, and 40 nm AuNPs with different laser intensities. Circles and rectangles with green, blue, and red colors represent signal and noise of 20 nm, 30 nm, and 40 nm AuNPs, respectively. (*d*) Signal-to-noise ratio of 20 nm, 30 nm, and 40 nm AuNPs at different laser intensities. Green, blue, and red circles represent 20 nm, 30 nm, and 40 nm AuNPs, respectively.

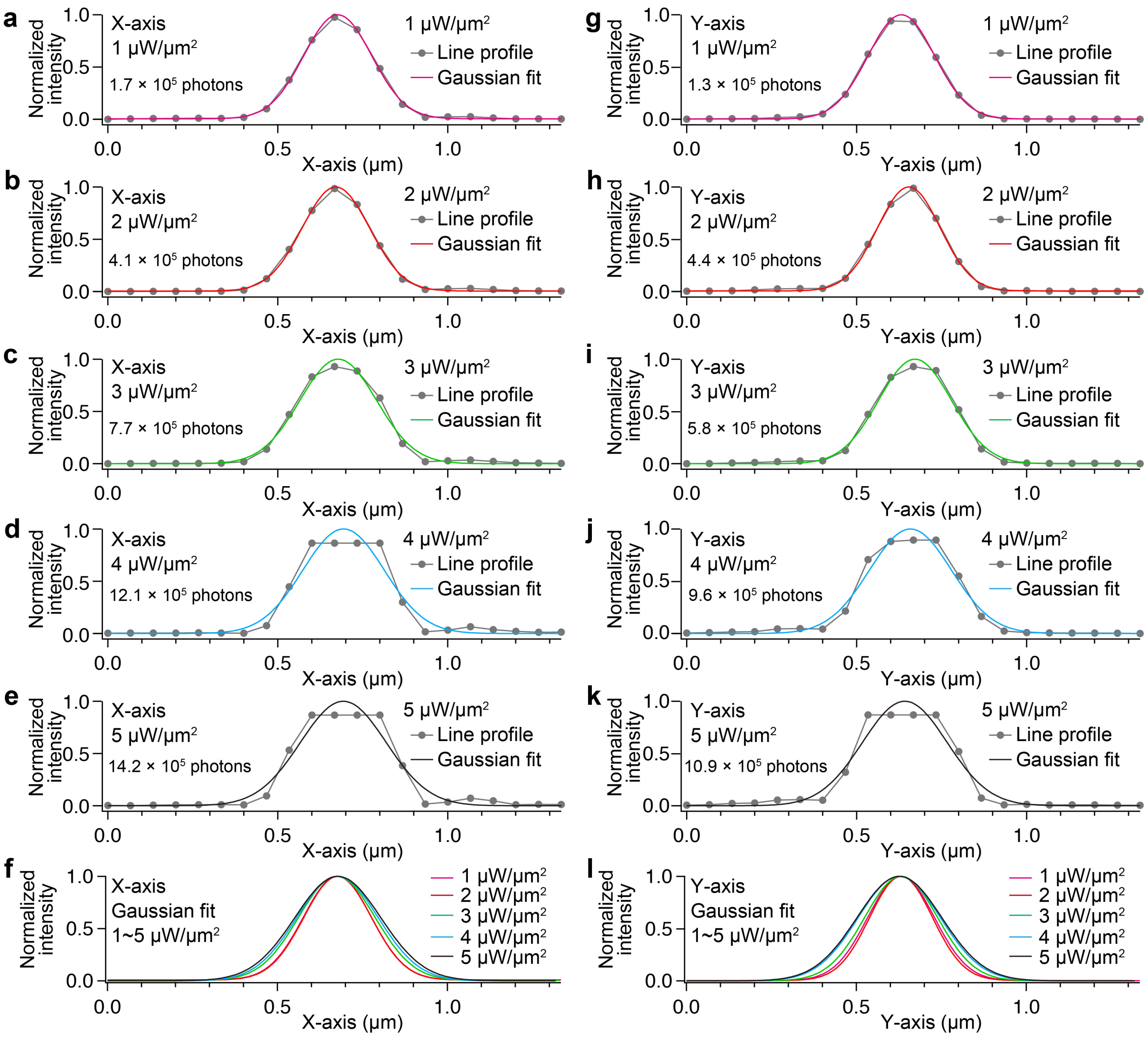


**Fig. S6.** Examples of line profile and Gaussian fit along X- (*a-e*) and Y-axis (*g-k*) for the dark-field image of 40 nm AuNPs. Images were taken at 1 ms time resolution with laser intensities of (*a*, *g*) 1 μW/μm$^2$, (*b*, *h*) 2 μW/μm$^2$, (*c*, *i*) 3 μW/μm$^2$, (*d*, *j*) 4 μW/μm$^2$, and (*e*, *k*) 5 μW/μm$^2$, respectively. (*f* and *l*) Overlay of the Gaussian fits in *a-e* (*f*) and *g-k* (*l*). Estimated photon numbers from AuNPs are also shown.

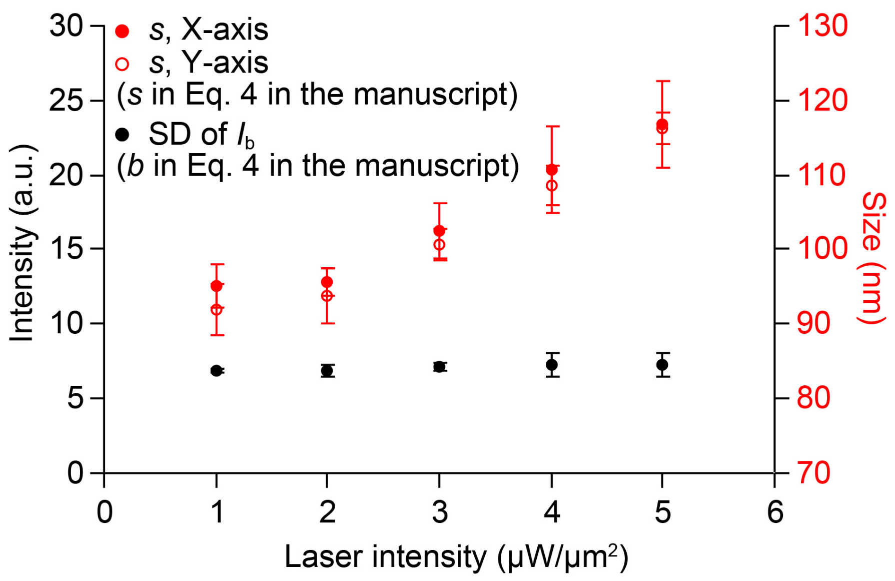


**Fig. S7.** Laser intensity dependences of the width of dark-field images of 40 nm AuNPs along X- and Y-axis (*s* in Eq. 4 in the manuscript) and standard deviation of background intensity (SD of $I_b$, *b* in Eq. 4 in the manuscript). Images were taken at 1 ms time resolution with different laser intensities of 1–5 μW/μm$^2$. Background intensity was measured at neighboring area of the AuNP.

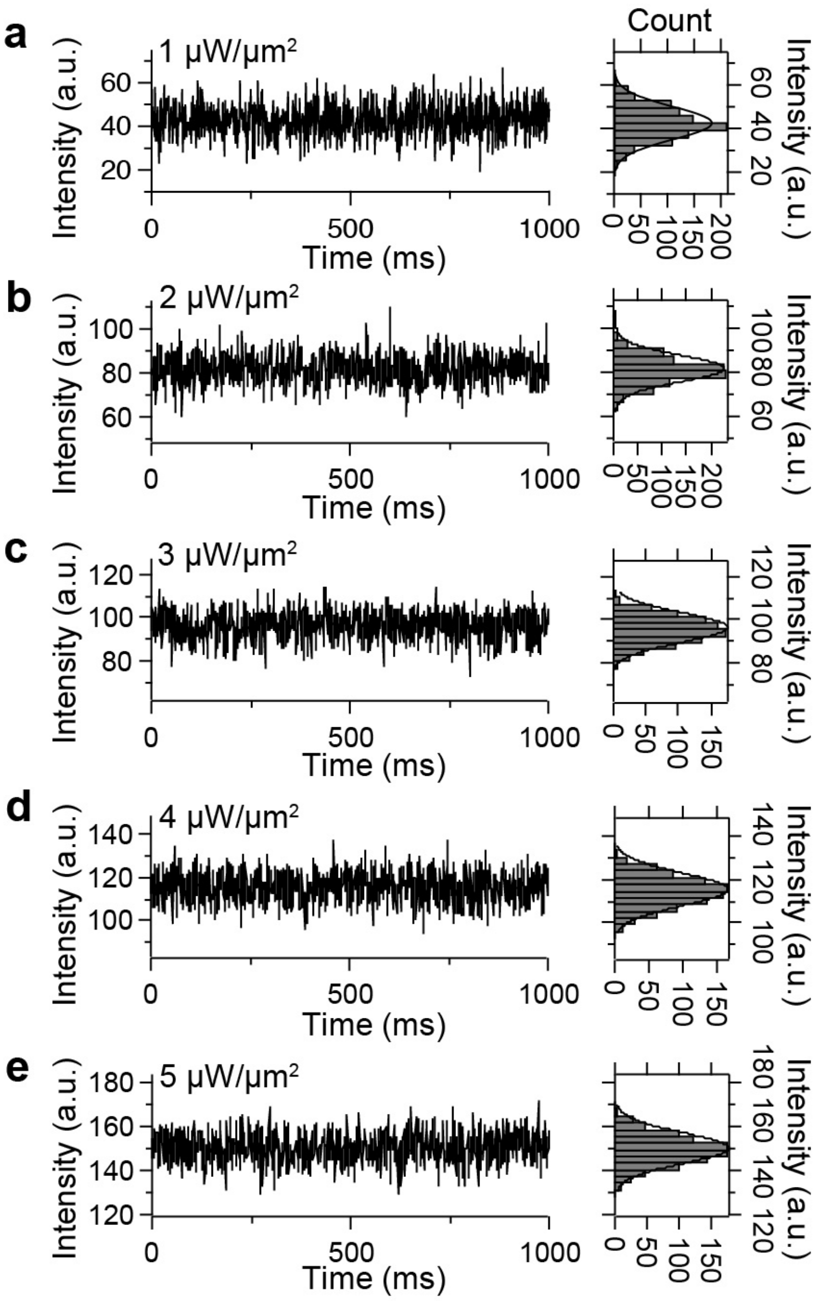


**Fig. S8.** Examples of the time courses and histograms with Gaussian fits of the background intensity in dark-field images of 40 nm AuNPs with laser intensities at (*a*) 1 μW/μm$^2$, (*b*) 2 μW/μm$^2$, (*c*) 3 μW/μm$^2$, (*d*) 4 μW/μm$^2$, and (*e*) 5 μW/μm$^2$. Background intensity was measured at neighboring area of the AuNP. Images were taken at 1 ms time resolution for 1.0 s.

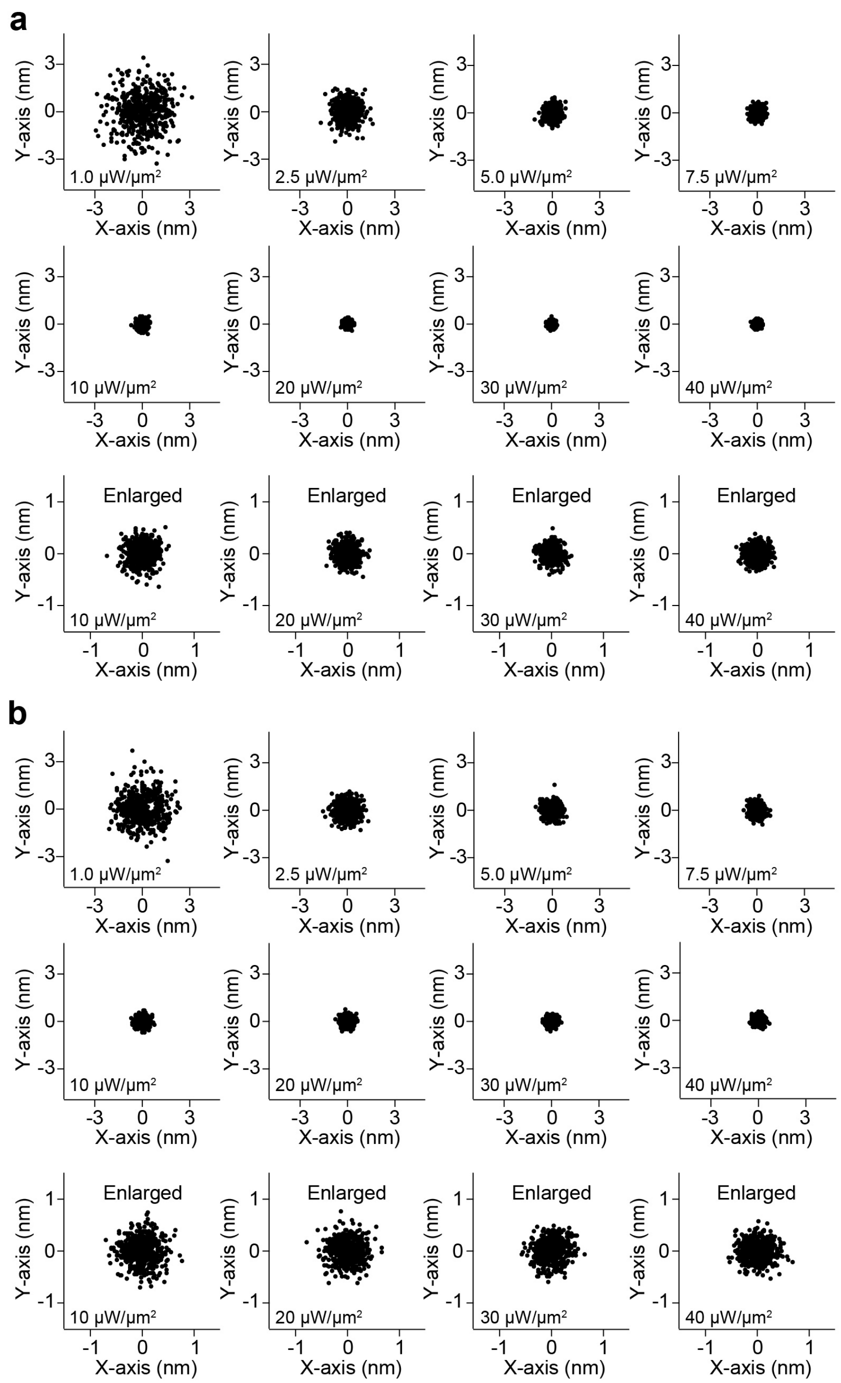


**Fig. S9.** Examples of two-dimensional plots at the center of the dark-field image of 40 nm AuNPs with laser intensities ranging from 1 to 40 μW/μm$^2$. The images were taken at 1 ms time resolution for 0.5 s and at image pixel sizes of (*a*) 31.6 nm/pixel and (*b*) 67.6 nm/pixel, respectively.

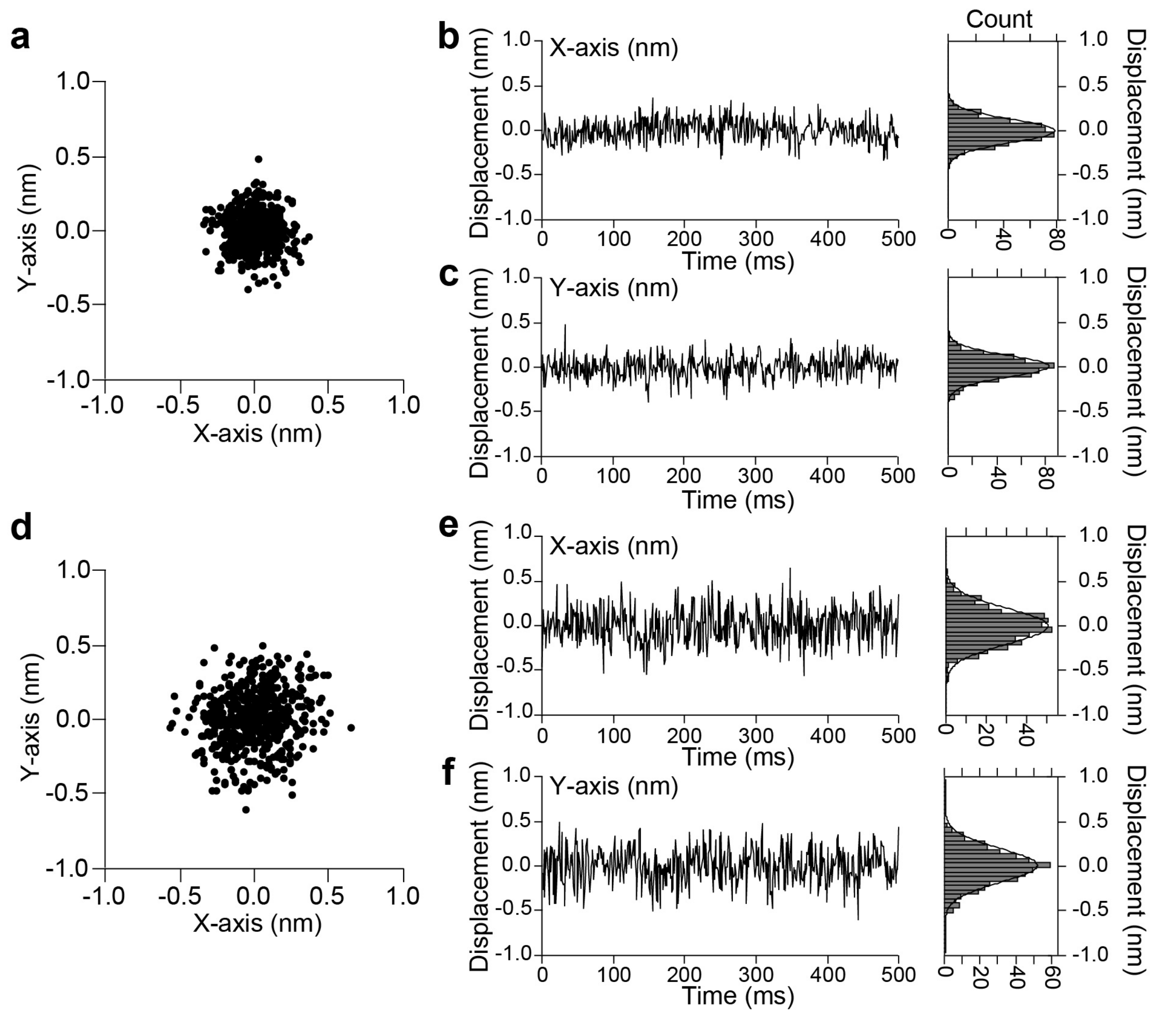


**Fig. S10.** Examples of two-dimensional plots, time courses, and histograms with Gaussian fits at the center of the dark-field image of 40 nm AuNPs, taken with image pixel sizes of (*a-c*) 31.6 nm/pixel and (*d-f*) 67.6 nm/pixel. The images were taken at 1 ms time resolution for 0.5 s and 30 μW/μm$^2$ laser intensity.

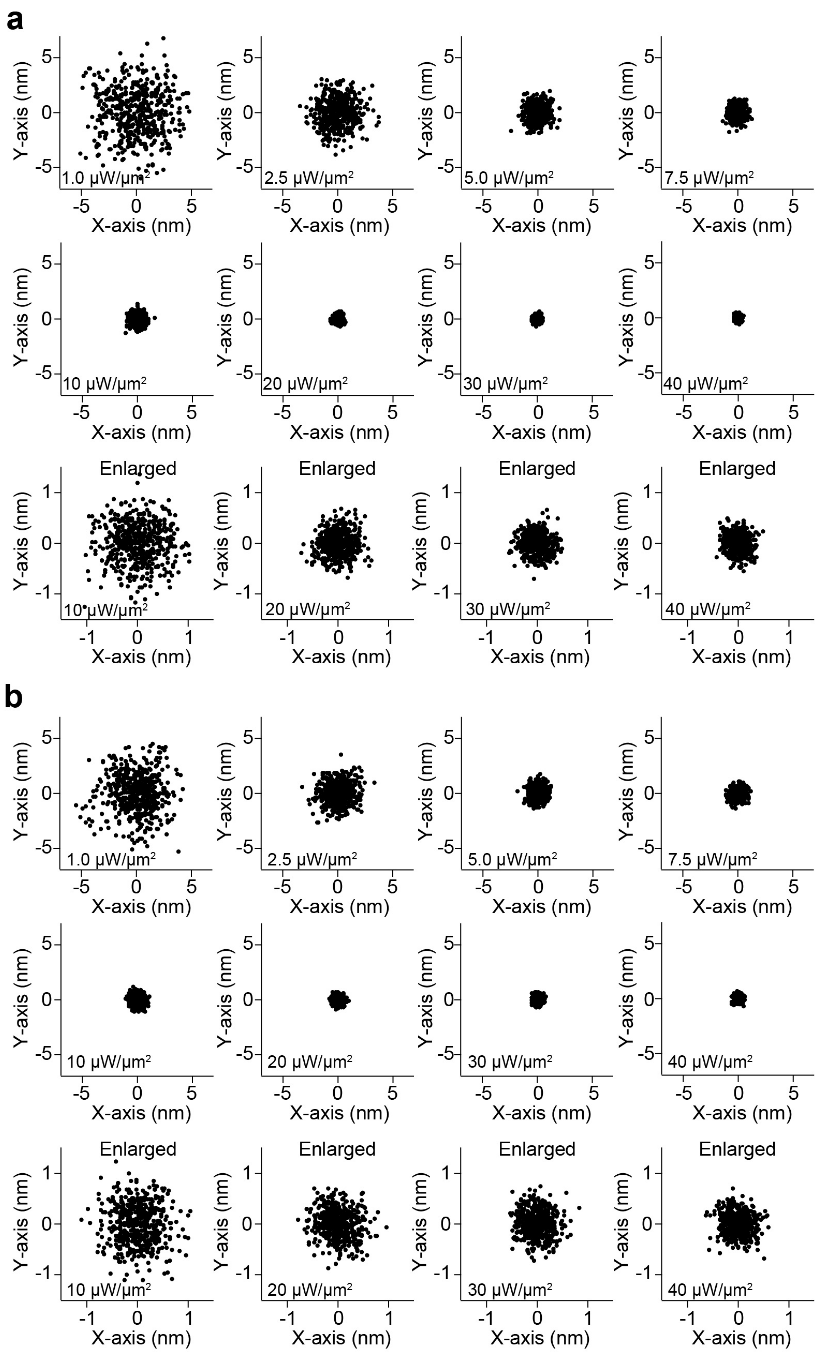


**Fig. S11.** Examples of two-dimensional plots in the center of the dark-field image of 30 nm AuNPs with laser intensities ranging from 1 to 40 μW/μm$^2$. The images were taken at 1 ms time resolution for 0.5 s. The images were taken at image pixel sizes of (*a*) 31.6 nm/pixel and (*b*) 67.6 nm/pixel.

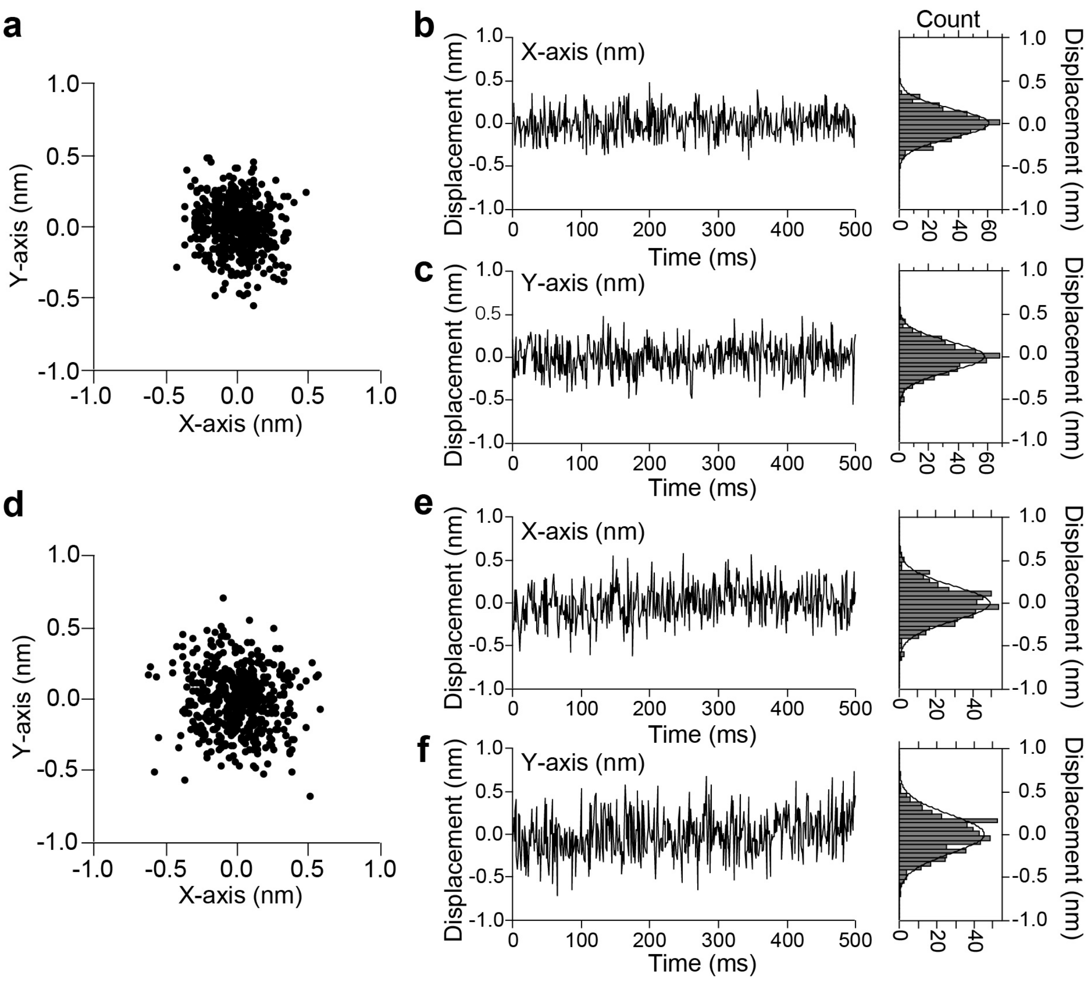


**Fig. S12.** Examples of two-dimensional plots, time courses, and histograms with Gaussian fits at the center of the dark-field image of 30 nm AuNPs, taken with image pixel sizes of (*a-c*) 31.6 nm/pixel and (*d-f*) 67.6 nm/pixel. The images were taken at 1 ms time resolution for 0.5 s and 40 μW/μm$^2$ laser intensity.

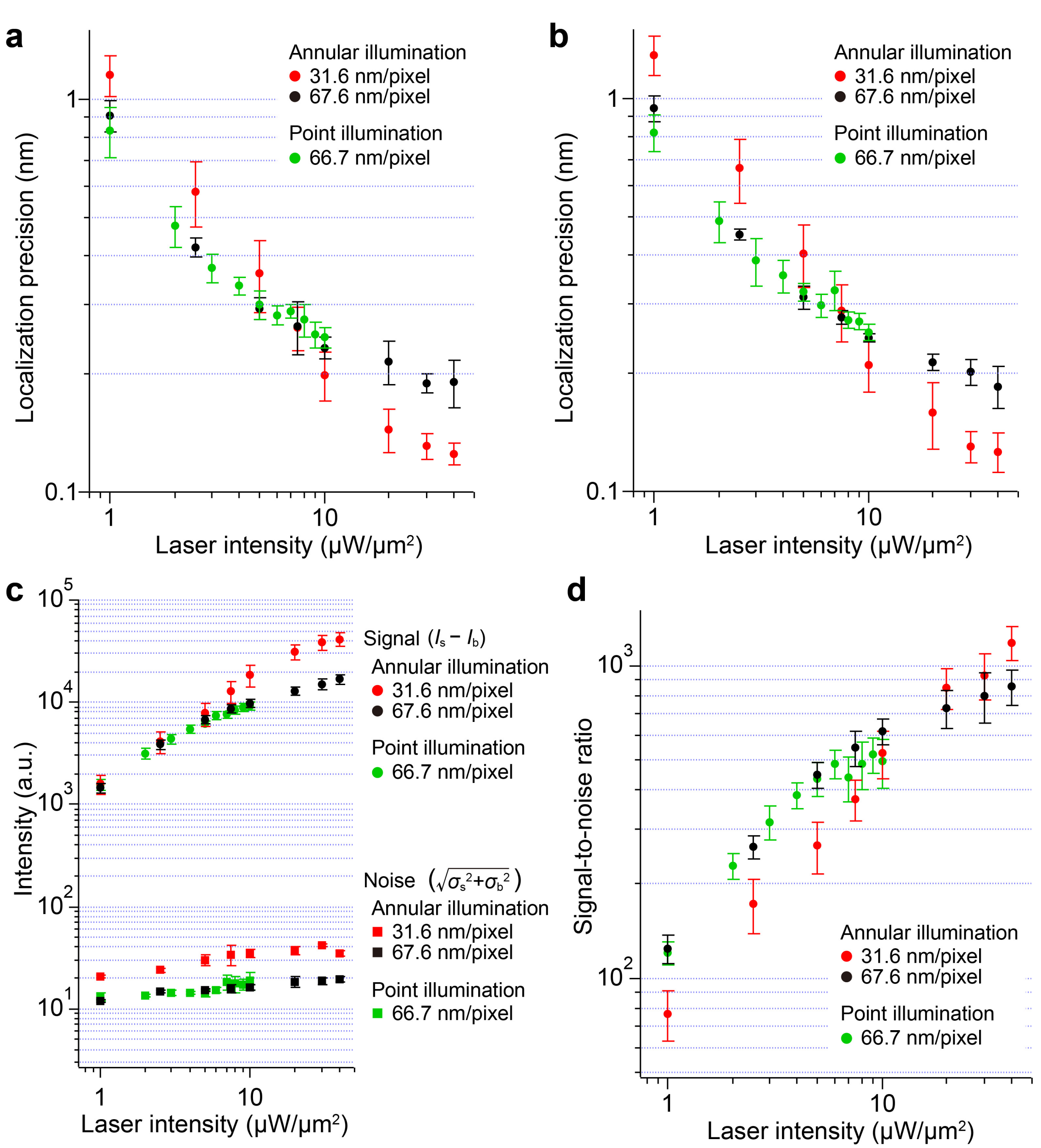


**Fig. S13.** Comparison of localization precision, signal, noise, and signal-to-noise ratio for 40 nm AuNP images between annular and point illumination total internal reflection dark-field microscopy. Images were taken at 1 ms time resolution. (*a, b*) Log-log plots between laser intensity and localization precision along the (*a*) X- and (*b*) Y-axis ($n$ = 5). Red and black circles represent annular illumination with 31.6 nm/pixel and 67.6 nm/pixel, respectively. Green circles

represent point illumination with 66.7 nm/pixel. (*c*) Log-log plots between laser intensity and signal (circles) or noise (squares) ($n$ = 5). Red and black symbols represent annular illumination with 31.6 nm/pixel and 67.6 nm/pixel, respectively. Green symbols represent point illumination with 66.7 nm/pixel. (*d*) Log-log plots between laser intensity and signal-to-noise ratio ($n$ = 5). Red and black circles represent annular illumination with 31.6 nm/pixel and 67.6 nm/pixel, respectively. Green circles represent point illumination with 66.7 nm/pixel.

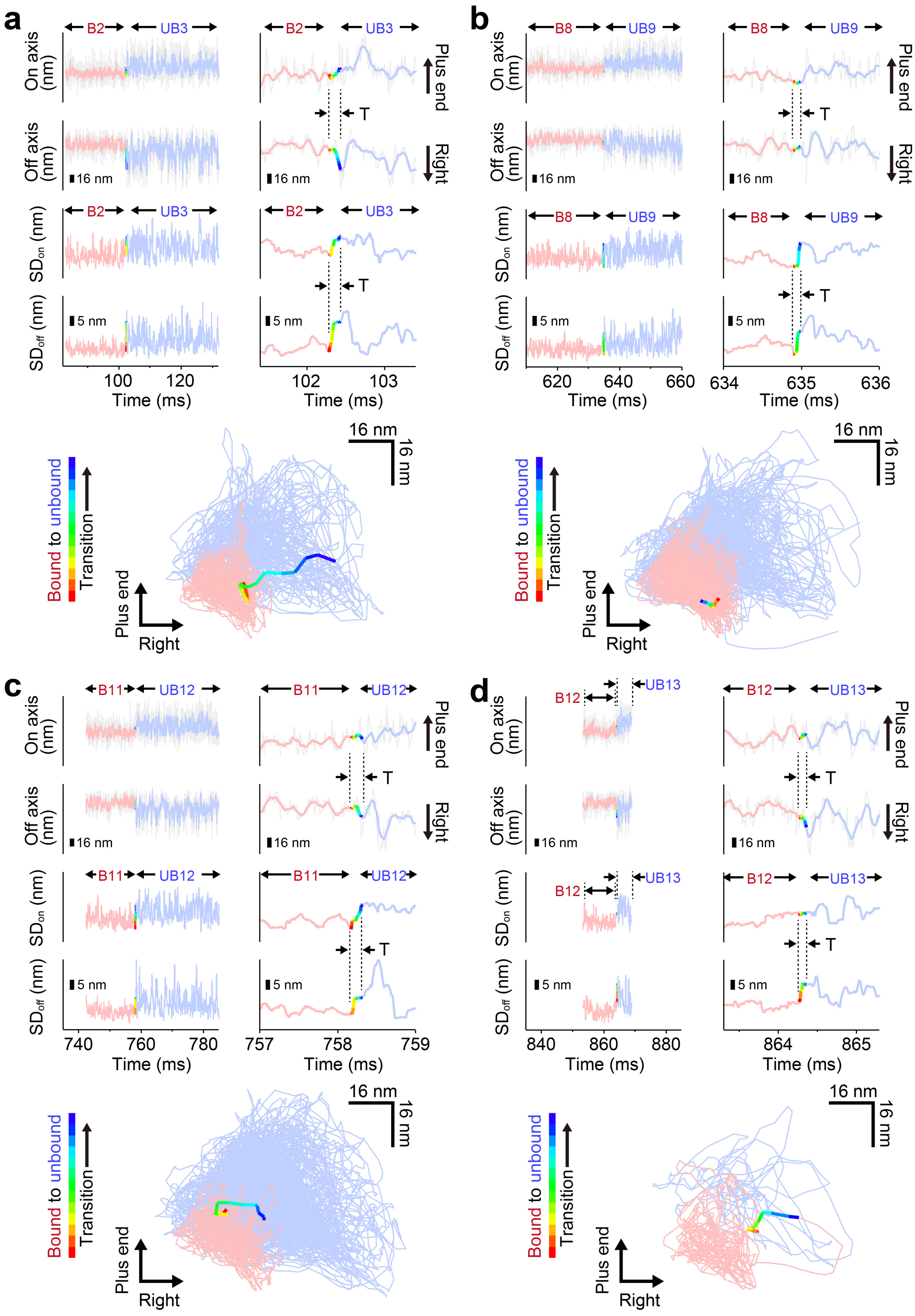
a
b
c
d
B2
UB3
B8
UB9
B11
UB12
B12
UB13
On axis (nm)
Off axis (nm)
SDon (nm)
SDoff (nm)
16 nm
5 nm
T
Plus end
Right
Time (ms)
100
120
102
103
620
640
660
634
635
636
740
760
780
757
758
759
840
860
880
864
865
Bound to unbound
Transition

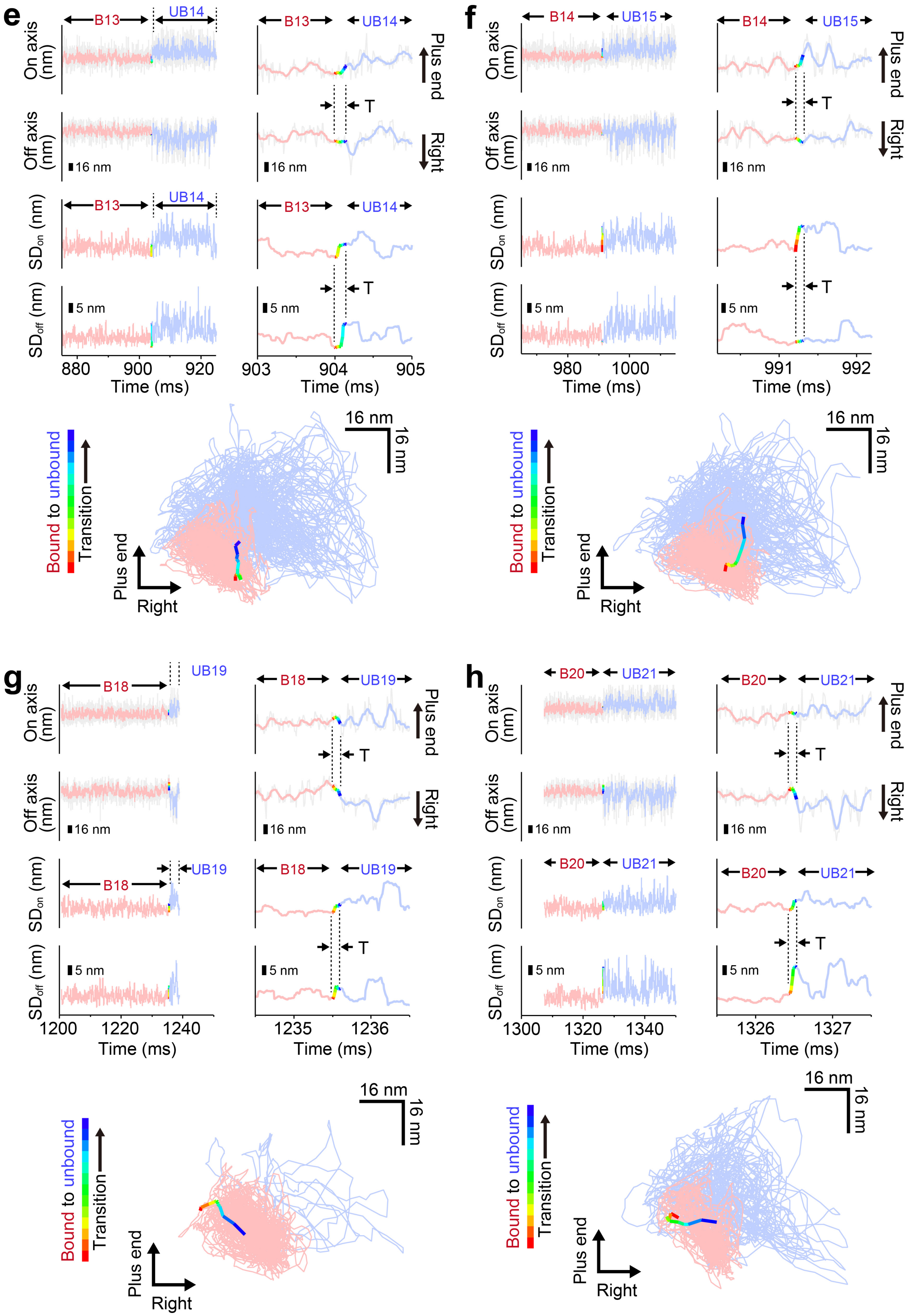
e
f
g
h
B13
UB14
B14
UB15
B18
UB19
B20
UB21
On axis (nm)
Off axis (nm)
SD$_{on}$ (nm)
SD$_{off}$ (nm)
16 nm
5 nm
T
Plus end
Right
Time (ms)
880
900
920
903
904
905
980
1000
991
992
1200
1220
1240
1235
1236
1300
1320
1340
1326
1327
Bound to unbound
Transition

**Fig. S14.** Examples of the transition from bound to unbound state of kinesin-1 head labeled with 40 nm AuNP at 10 μs time resolution. (*a-f*) Upper panels show enlarged traces of Fig. 7*a*, showing the center position of AuNP (gray lines) in on- and off-axis, and SD of the position of AuNP for each time (calculated as [t - 10, t + 10]) in on- and off-axis. Numbers correspond to the location of bound and unbound state, shown in Fig. 7*a*. Light red, light blue and rainbow-colored lines represent the filtered trace with moving average (window size of 10 frames) for bound state, unbound state and transition from bound to unbound state, respectively. Lower panel shows two-dimensional plot of the center position of AuNP attached to kinesin-1 head during transition, shown in upper panel. Light red, light blue and rainbow lines represent filtered trace with moving average (window size of 10 frames) for bound state, unbound state and transition from bound to unbound state, respectively.

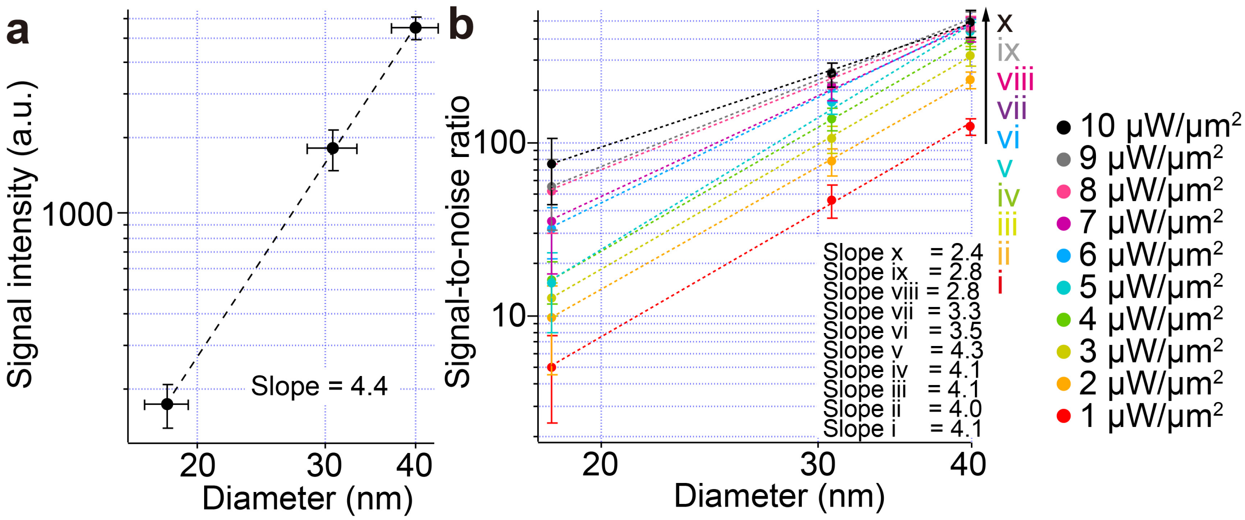


**Fig. S15.** Dependence of signal intensity and signal-to-noise ratio on particle size. (*a*) A log-log plot between AuNP diameter and scattering signal intensity in the dark-field image ($n$ = 5). Dark-field images were taken at 1 ms time resolution and 4 μW/μm$^2$ laser intensity. The dotted line shows a linear fit. The slope of the plot was 4.4. (*b*) Log-log plots between AuNP diameter and signal-to-noise-ratio of the dark-field image ($n$ = 5). Dark-field images were taken at 1 ms time resolution and 1–10 μW/μm$^2$ laser intensity. Dotted lines show linear fits for different laser intensities. The slopes of the dotted lines were 4.1 (i), 4.0 (ii), 4.1 (iii), 4.1 (iv), 4.3 (v), 3.5 (vi), 3.3 (vii), 2.8 (viii), 2.8 (ix), and 2.4 (x), respectively. Note that the error bars for particle diameters were omitted as they are the same size as in (*a*).

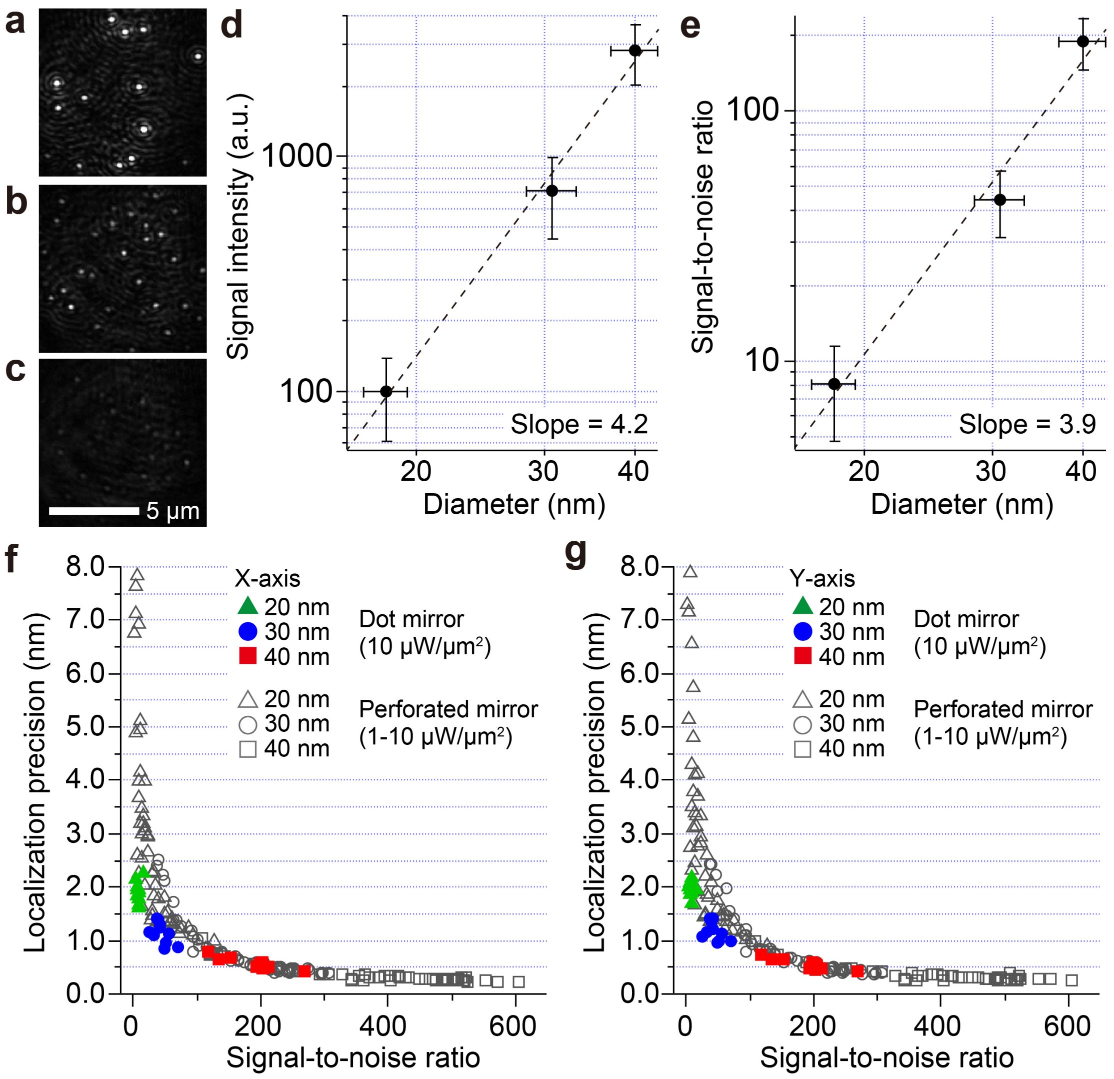


**Fig. S16.** Dark-field images of AuNPs with vertical illumination dark-field imaging system using dot-mirror, dependence of signal intensity and signal-to-noise ratio on particle size, and relationship between localization precision and signal-to-noise ratio. (*a-c*) Dark-field images of (*a*) 40 nm, (*b*) 30 nm, and (*c*) 20 nm AuNPs, taken at 1 ms time resolution with 10 μW/μm$^2$ laser intensity. Scale bar is 5 μm. (*d* and *e*) Log-log plots between AuNP diameter and scattering signal intensity (*d*) or signal-to-noise-ratio (*e*) ($n$ = 10). The dotted lines show a linear fit. The slopes of the fit were 4.2 (*d*) and 3.9 (*e*). (*f* and *g*) Relationship between signal-to-noise ratio and localization precision along the (*f*) X- and (*g*) Y-axis. Green triangles, blue circles and red squares represent 20 nm, 30 nm, and 40 nm AuNPs, respectively, observed by vertical

illumination dark-field imaging system using dot-mirror with 10 $\mu W/\mu m^2$ laser intensity. Gray-colored triangles, circles, and squares represent 20 nm, 30 nm, and 40 nm AuNPs observed by total internal reflection dark-field imaging system using perforated mirror with 1- 10 $\mu W/\mu m^2$ laser intensity.

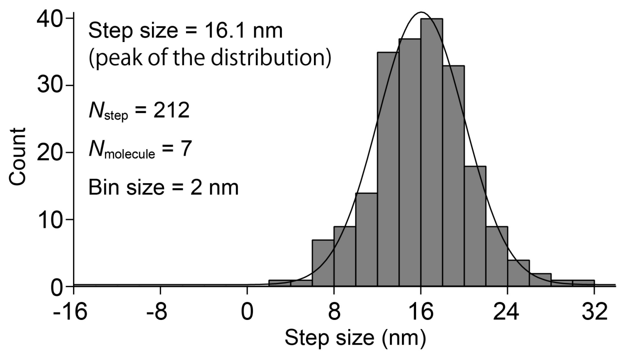


**Fig. S17.** Distribution of step size of kinesin-1 head labeled with 30 nm AuNP at 50 μs time resolution and at 10 μM ATP. Peak position of the distribution was 16.1 nm, obtained by the Gaussian fit shown as solid line (7 molecules, 212 steps).

**Video S1.** Video of the Dark-Field Image of 40 nm AuNP Attached to a Kinesin-1 Head at 10 μs Time Resolution and at 10 μM ATP. The movie was replayed at 100 frames per second. Upper side of the movie directs to the plus end of the microtubule. Image size is 0.81 x 0.95 μm.

**Video S2.** Video of the Dark-Field Image of 30 nm AuNP Attached to a Kinesin-1 Head at 50 μs Time Resolution and at 10 μM ATP. The movie was replayed at 100 frames per second. Upper side of the movie directs to the plus end of the microtubule. Image size is 0.81 x 0.95 μm.
